# Mining the Flavoproteome of Brucella ovis, the Brucellosis Causing Agent in Ovis aries

**DOI:** 10.1128/spectrum.02294-21

**Published:** 2022-03-22

**Authors:** Martha Minjárez-Sáenz, Marta Martínez-Júlvez, Inmaculada Yruela, Milagros Medina

**Affiliations:** a Departamento de Bioquímica y Biología Molecular y Celular, Facultad de Ciencias, Universidad de Zaragoza, Zaragoza, Spain; b Instituto de Biocomputación y Física de Sistemas Complejos (BIFI), Universidad de Zaragoza, Zaragoza, Spain; c Estación Experimental de Aula Dei, CSIC, Zaragoza, Spain; d Group of Biochemistry, Biophysics and Computational Biology “GBsC” (BIFI, Unizar) Joint Unit to CSIC, Zaragoza, Spain; University of California, Davis

**Keywords:** *Brucella ovis*, flavoproteome, alpha-proteobacteria, flavoenzymes, metabolic function

## Abstract

Flavoproteins are a diverse class of proteins that are mostly enzymes and contain as cofactors flavin mononucleotide (FMN) and/or flavin adenine dinucleotide (FAD), which enable them to participate in a wide range of physiological reactions. We have compiled 78 potential proteins building the flavoproteome of Brucella ovis (B. ovis), the causative agent of ovine brucellosis. The curated list of flavoproteins here reported is based on (i) the analysis of sequence, structure and function of homologous proteins, and their classification according to their structural domains, clans, and expected enzymatic functions; (ii) the constructed phylogenetic trees of enzyme functional classes using 19 Brucella strains and 26 pathogenic and/or biotechnological relevant alphaproteobacteria together with B. ovis; and (iii) the evaluation of the genetic context for each entry. Candidates account for ∼2.7% of the B. ovis proteome, and 75% of them use FAD as cofactor. Only 55% of these flavoproteins belong to the core proteome of Brucella and contribute to B. ovis processes involved in maintenance activities, survival and response to stress, virulence, and/or infectivity. Several of the predicted flavoproteins are highly divergent in Brucella genus from revised proteins and for them it is difficult to envisage a clear function. This might indicate modified catalytic activities or even divergent processes and mechanisms still not identified. We have also detected the lack of some functional flavoenzymes in B. ovis, which might contribute to it being nonzoonotic. Finally, potentiality of B. ovis flavoproteome as the source of antimicrobial targets or biocatalyst is discussed.

**IMPORTANCE** Some microorganisms depend heavily on flavin-dependent activities, but others maintain them at a minimum. Knowledge about flavoprotein content and functions in different microorganisms will help to identify their metabolic requirements, as well as to benefit either industry or health. Currently, most flavoproteins from the sheep pathogen Brucella ovis are only automatically annotated in databases, and only two have been experimentally studied. Indeed, certain homologues with unknown function are not characterized, and they might relate to still not identified mechanisms or processes. Our research has identified 78 members that comprise its flavoproteome, 76 of them flavoenzymes, which mainly relate to bacteria survival, virulence, and/or infectivity. The list of flavoproteins here presented allows us to better understand the peculiarities of Brucella ovis and can be applied as a tool to search for candidates as new biocatalyst or antimicrobial targets.

## INTRODUCTION

Flavoproteins contain as cofactors the riboflavin (RF) derivatives flavin mononucleotide (FMN) and/or flavin adenine dinucleotide (FAD), and participate in a wide range of physiological reactions and metabolic pathways (1–4). Most of them are essential mediators in oxido-reduction processes, where they can either exchange one or two electrons, whereas other cofactors or coenzymes exclusively catalyze one- (iron-sulfur clusters, haem groups, etc.) or two- (nicotinamide adenine nucleotides) electron transfer processes. This makes flavoenzymes exhibit their redox versatility in a large number of metabolic redox processes. Moreover, among the around 500 different flavin-dependent proteins so far identified ∼10% catalyze nonredox reactions or act as signaling and sensing molecules (5–7). Examples would include signal transduction in programmed cell death, embryonic development, chromatin remodeling, nucleotide synthesis, tRNA methylation, protein folding, and defense against oxidative stress (8–15), among others. Some flavoproteins are also involved in the xenobiotic metabolism of aromatic compounds, in pathogens virulence, and in light-dependent processes in which flavin receives photons: as luciferase light-emission, DNA reparation, plant phototropism, and cellular clocks (16–22). Many flavoenzymes are also suitable biocatalysts due to their selectivity, control, and efficiency of the reactions they catalyze (3, 23), as well as therapeutic targets both in the treatment of infectious diseases and in mammalian pathological situations (24–32). Despite their potential, only a few flavoproteins are widely exploited (33). To expand their applicability, it is essential to investigate the flavoprotein content and diversity in different organisms. So far, detailed information of flavoproteomes has only been reported in Homo sapiens, Saccharomyces cerevisiae, and Arabidopsis thaliana (5, 6, 34, 35), to which has to be added a comparative analysis in a bunch of archaeal, eubacterial, protozoan, and eukaryotic genomes (7). These studies indicate that whereas some organisms depend heavily on flavin-dependent activities, others maintain a minimum of flavoproteins. A better knowledge about content of flavoproteomes would surely help in the understanding of metabolic requirements in different organisms, and benefit either industry or health.

In this context, we aimed to trace flavin-binding proteins in Brucella ovis (B. ovis), a Gram-negative bacteria that provokes placentitis in sheep and genital lesions in rams that affect the quality of the semen and the fertility, and causes major economic impacts in countries and regions with sheep (Ovis aries) breeding activity (36). The virulence of each particular Brucella species depends on enzymes and cell envelope proteins that act as virulence factors, and on the ability to fight against the host response (37). Nonetheless, which genes and proteins are essential in these processes, as well as how they interact during intracellular virulence, still remain unclear. Here, we have identified 78 candidates to constitute the B. ovis flavoproteome. We envisage a metabolic function for many of them upon evaluation of their presence in Brucella and pathogenic and biotechnological relevant alphaproteobacteria (Table SP1 in the supplemental material), as well as of their evolutionary fingerprint and genetic context. Our studies point to a list of flavoproteins with high probability to contribute to the B. ovis survival, virulence, and/or infectivity, some of which have not yet been characterized in any homologue. This list is also discussed as a tool in the search for candidates as new biocatalyst or antimicrobial targets.

## RESULTS

### Overall features of the Brucella ovis flavoproteome.

The Brucella ovis ATCC 25840 genome contains 2,890 genes organized in two chromosomes, CI (2.10 Mb, 1,928 genes) and CII (1.15 Mb, 962 genes) (18). Among them, we identified 78 flavoproteins encoded by 49 genes of CI (2.4%) and 37 genes of CII (3.5%). They constitute the curated flavoproteome of B. ovis (Tables 1 and 2) and represent ∼2.7% of the B. ovis proteome. This percentage agrees with average estimations from the study of other flavoproteomes (5–7, 34).

**TABLE 1:** Predicted flavoproteins encoded in the chromosome I of Brucella ovis ATCC 25840

Entry	EC	Protein	Pfam domain^*a*^	Locus tag	Protein code	Flavin	Coenzyme or ligand	More similar PDB (% identity)
1	1.1.5.3	Glycerol-3-phosphate dehydrogenase	DAO (PF01266) 19-347	BOV_RS00950 (glpD)	ABQ60174.1	FAD	Quinone	2QCU (50)
			DAO_C (PF16901) 399-508					
2	1.1.99.1	Choline dehydrogenase (Glucose-methanol-choline GMC family)	GMC_oxred_N (PF00732) 4-292	BOV_RS02765 (betA)	ABQ61350.1	FAD		2JBV (35)
			GMC_oxred_C (PF05199) 384-521					
3	1.1.99.1	Choline dehydrogenase (GMC family)	GMC_oxred_N (PF00732) 4-295	BOV_RS07775	ABQ60630.1	FAD		4HA6 (31)
			GMC_oxred_C (PF05199) 388-524					
4	1.1.-.-^*b*^	***Potential FAD-binding oxygen oxidoreductase (glcE?)*** ^*c*^	FAD_binding_4 (PF01565) 54-187	BOV_RS06750	ABQ60928.1	FAD		3PM9 (29)
			FAD-oxidase_C (PF02913) 223-463					
5	1.1.-.-^*b*^	***Potential FAD-binding oxygen oxidoreductase (glcE?)***	FAD_binding_4 (PF01565) 43-180	BOV_RS02095	ABQ61939.1	FAD		3PM9 (60)
			FAD-oxidase_C (PF02913) 219-469					
6	1.3.1.1	NADH dependent Dihydropyrimidine dehydrogenase subunit PreA	DHO_dh (PF01180) 4-307	BOV_RS01510 (preA)	ABQ60560.1	FMN		2B4G (24) 1GTE (37)
			Fer4_21 (PF14697) 340-400				2x(4Fe-4S)	
		NADH dependent Dihydropyrimidine dehydrogenase subunit PreT	Fer4_20 (PF14691) 31-139	BOV_RS01515 (preT)	ABQ61103.1		4Fe-4S	5JCA_L (32) 5VJ7_A (35)
			Pyr_redox_2 (PF07992) 153-441			FAD	NAD(P)H	
7	1.3.1.88	tRNA dihydrouridine synthase B	Dus (PF01207) 22-316	BOV_RS05355 (DusB)	ABQ61416.1	FMN	NAD(P)H	6EI9 (41)
8	1.3.1.91	tRNA dihydrouridine20/20a synthase	Dus (PF01207) 21-320	BOV_RS04255 (dusA)	ABQ61966.1	FMN	NAD(P)H	3B0P (46)
9	1.3.1.98	UDP-N-acetylmuramate dehydrogenase	FAD_binding_4 (PF01565) 42-172	BOV_RS06850 (MurB)	ABQ61769.1	FAD	NADPH	3TX1 (35)
			murB_C (PF02873) 206-304					
10	1.3.5.1	Succinate dehydrogenase flavoprotein subunit	FAD_binding_2 (PF00890) 24-419	BOV_RS08985 (sdhA)	ABQ61077.1	FAD		2H88_A (63)
			Succ_DH_flav_C (PF02910) 474-613					
11	1.3.5.2	PyrD dihydroorotate dehydrogenase 2 (quinone)	DHO_dh (PF01180) 44-336	BOV_RS01655 (pyrD)	ABQ61413.1	FMN	Quinone	4ORI (48)
12	1.3.8.1	Short Chain Acyl-CoA dehydrogenase	Acyl-CoA_dh_N (PF02771) 37-155	BOV_RS02120	ABQ60180.1	FAD		1BUC (29)
			Acyl-CoA_dh_M (PF02770) 160-268					
			Acyl-CoA_dh_1 (PF00441) 288-457					
			Acyl-CoA_dh_C (PF12806) 470-585					
13	1.3.8.4	Isovaleryl-CoA dehydrogenase	Acyl-CoA_dh_N (PF02771) 7-118	BOV_RS00090 (ivd)	ABQ60382.1	FAD		4KTO (83) 4O5M (100)
			Acyl-CoA_dh_M (PF02770) 122-217					
			Acyl-CoA_dh_1 (PF00441) 229-377					
14	1.3.8.-^*b*^	Acyl-CoA dehydrogenase	Acyl-CoA_dh_N (PF02771) 12-123	BOV_RS06310	ABQ61585.1	FAD		1RX0 (57)
			Acyl-CoA_dh_M (PF02770) 127-221					
			Acyl-CoA_dh_1 (PF00441) 235-382					
15	1.4.3.5	Pyridoxamine 5′-phosphate oxidase	Putative_PNPOx (PF01243) 29-113	BOV_RS02140 (pdxH)	ABQ60142.1	FMN		1NDL (45)
			PNP_phzG_C (PF10590) 166-208					
16	1.4.3.19	Glycine oxidase ThiO	DAO (PF01266) 3-313	BOV_RS01020 (thiO)	ABQ60316.1	FAD		4YSH (27)
17	1.4.3.-^*b*^	***Potential Aminoacetone oxidase family FAD-binding enzyme/ NAD(P)/FAD-dependent dehydrogenase***	HI0933_like (PF03486) 5-391	BOV_RS06670	ABQ60616.1	FAD		3V76 (67)
18	1.4.3.-^*b*^	***Potential Aminoacetone oxidase family FAD-binding enzyme/ NAD(P)/FAD-dependent dehydrogenase***	HI0933_like (PF03486) 5-391	BOV_RS04985	ABQ60524.1	FAD		2I0Z (24)
19	1.4.99.-^*b*^	Predicted D-amino acid dehydrogenase small subunit	DAO (PF01266) 10-400	BOV_RS08480	ABQ61937.1	FAD		6J38 (23)
20	1.4.-.-^*b*^	***Pyridoxamine 5′-phosphate oxidase family protein***	Pyridox_ox_2 (PF12900) 10-141	BOV_RS06575	ABQ61684.1	FMN		3U0I (99) 2HQ9 (30)
21	1.5.1.20	Methylenetetra-hydrofolate reductase	MTHFR (PF02219) 14-291	BOV_RS06945 (metF)	ABQ60279.1	FAD	NAD(P)H	3FST (48)
22	1.5.1.-^*b*^	***Flavin reductase domain containing protein***	Flavin_Reduct (PF01613) 22-172	BOV_RS05125	ABQ60228.1	FMN	NAD(P)H	1EJE (29)
23	1.5.3.1	Sarcosine oxidase beta subunit	DAO (PF01266) 36-161	BOV_RS01075 (soxB_1)	ABQ60177.1	FAD & FMN		2GAG_B (64)
			DAO (PF01266) 8-222	BOV_RS01090 (soxB_2)	ABQ61310.1			2GAG_B (58)
		Sarcosine oxidase alpha subunit	Fer2_4 (PF13510) 16-102	BOV_RS01100	ABQ61036.1	FMN	NADH 4Fe-4S	2GAG_A (47)
			FAD_oxidored (PF12831) 171-218					
			GCV_T (PF01571) 526-790					
			GCV_T_C (PF08669) 815-901					
24	1.5.5.1	Electron transferring flavoprotein-ubiquinone oxidoreductase (ETF-QO)	Thi4 (PF01946) 12-52	BOV_RS03100	ABQ61337.1	FAD		2GMH (49)
			ETF_QO (PF05187) 451-560				4Fe-4S quinone	
25	1.6.5.2	WrpA type FMN-dependent NADH:quinone oxidoreductase	FMN_red (PF03358) 13-145	BOV_RS05025	ABQ60884.1	FMN	NAD(P)H quinone	5F4B (98)
26	1.7.-.-^*b*^	***Predicted NAD(P)H nitroreductase***	Nitroreductase (PF00881) 23-167	BOV_RS05130	ABQ60834.1	FMN	NAD(P)H	3K6H (43)
27	1.8.1.4	Dihydrolipoyl dehydrogenase (IpdA-2)	Pyr_redox_2 (PF07992) 57-399	BOV_RS05390 (lpdA-2)	ABQ60398.1	FAD	NADH	2A8X (43)
			Pyr_redox_dim (PF02852) 420-528					
28	1.8.1.4	Dihydrolipoyl dehydrogenase (IpdA-3)	Pyr_redox_2 (PF07992) 3-329	BOV_RS09065 (lpdA-3)	ABQ61458.1	FAD	NADH	3URH (78)
			Pyr_redox_dim (PF02852) 348-456					
29	1.8.1.7	Glutathione-disulphide reductase	Pyr_redox_2 (PF07992) 6-321	BOV_RS04850 (gor)	ABQ61016.1	FAD	NADPH	4DNA (69)
			Pyr_redox_dim (PF02852) 341-449					
30	1.8.1.9	Thioredoxin-disulphide reductase	Pyr_redox_2 (PF07992) 8-302	BOV_RS07155 (trxB)	ABQ60123.1	FAD	NAD(P)H	4JNQ (100)
31	1.8.1.9	Predicted thioredoxin-disulphide reductase	Pyr_redox_2 (PF07992) 9-171	BOV_RS04925	ABQ61134.1	FAD	NADP(H)	5YGQ (49) ^*d*^ 1NHS (30) 5VJ7 (34)
32	1.14.13.1	Predicted Salicylate hydroxylase	FAD_binding_3 (PF01494) 6-349	BOV_RS03690	ABQ60137.1	FAD	NAD(P)H	4BJZ (32)
33	1.14.13.1	Predicted Salicylate hydroxylase	FAD_binding_3 (PF01494) 2-328	BOV_RS04715	ABQ60978.1	FAD	NAD(P)H	3RP8 (27)
34	1.14.13.-^*b*^	Predicted UbiH/COQ6 monooxygenase family	FAD_binding_3 (PF01494) 14-322	BOV_RS08970	ABQ60166.1	FAD	NAD(P)H	4K22 (37)
35	1.14.14.3	Bacterial luciferase	Bac_luciferase (PF00296) 8-255	BOV_RS09695	ABQ60348.1	FMN		3FGC (20)
36	1.16.1.4	Cob(II)alamin reductase	Flavin_Reduct (PF01613) 60-208	BOV_RS06210	ABQ60249.1	FMN		3CB0 (99)
37	1.17.1.4	Xanthine dehydrogenase, small subunit	Fer2 (PF00111) 13-59	BOV_RS01845 (xdhA)	ABQ61298.1		NADH 2(2Fe-2S)	2W3S_A (48)
			Fer2_2 (PF01799) 88-161					
			FAD_binding_5 (PF00941) 204-367			FAD		
			CO_deh_flav_C (PF03450) 376-476					
38	1.18.1.2	Ferredoxin-NADP^+^ reductase	FAD_binding_6 (PF00970) 19-102	BOV_RS01770 (fpr)	ABQ61707.1	FAD		6RRA (100)
			NAD_binding_1 (PF00175) 113-230				NADPH	
39	2.1.1.74	Methylenetetrahydrofolate-tRNA-(uracil54-C5-)-methyltransferase NAD(P)H oxidase	GIDA (PF01134) 10-378	BOV_RS04425 (trmFO)	ABQ61275.1	FAD	NADH	3G5S (46)
40	2.1.1.229	tRNA (carboxymethyluridine34-5-O)-methyltransferase	GIDA (PF01134) 10-399	BOV_RS09735 (mnmG)	ABQ60378.1	FAD	NADH	2ZXI (50)
			GIDA_assoc (PF13932) 402-612					
41	2.2.1.6	Acetolactate synthase 3 catalytic subunit	TPP_enzyme_N (PF02776) 1-165	BOV_RS06655 (ilvB)	ABQ60081.1	FAD	Thiamine diPP	6DEN (46)
			TPP_enzyme_M (PF00205) 191-327					
			TPP_enzyme_C (PF02775) 393-540					
42	2.5.1.9	Riboflavin synthase alpha subunit	Lum_binding (PF00677) 20-258	BOV_RS03790 (ribE)	ABQ60518.1	RF		4E0F (98)
43	4.2.3.5	Chorismate synthase	Chorismate_synt (PF01264) 10-355	BOV_RS02190 (aroC)	ABQ60200.1	FMN		1UM0 (42)
44	7.1.1.2	NADH-quinone oxidoreductase subunit F (H^+^ translocating)	Complex1_51K (PF01512) 47-216	BOV_RS04000 (nuoF)	ABQ60521.1			6Q9C_D (46)
			SLBB (PF10531) 242-292			FMN	NADH 4Fe-4S	
			NADH_4Fe-4S (PF10589) 332-414					
45		Electron transferring flavoprotein alpha subunit (ETFa)	ETF (PF01012) 44-210	BOV_RS09295 (etfA)	ABQ61011.1			1EFP_A (69)
			ETF_alpha (PF00766) 229-312			FAD		
		Electron transferring flavoprotein beta subunit (ETFb)	ETF (PF01012) 29-206	BOV_RS09300 (etfB)	ABQ60428.1		AMP	1EFP_B (72)

a Pfam domains include name, code, and residues in the B. ovis flavoprotein making the domain.

b Identified as flavoenzyme, but available information does not allow to fully predict its activity.

c Shown in bold and italics are those candidates for which a clear function cannot be depicted.

d More than one structure to represent the different regions of the protein.

**TABLE 2:** Predicted flavoproteins encoded in the chromosome II of Brucella ovis ATCC 25840

Entry	EC	Protein	Pfam domains^*a*^	Locus tag	Protein code	Flavin	Coenzyme or Ligand	More similar PDB (% identity)
46	1.1.2.3	L-lactate dehydrogenase (cytochrome c o b2)	FMN_dh (PF01070) 14-377	BOV_RS14715 (lldD)	ABQ62635.1	FMN		5ZBM (40)
47	1.1.-.-^*b*^	***Potential L-gulonolactone oxidase FAD-binding oxygen oxidoreductase*** ^*c*^	FAD_binding_4 (PF01565) 22-149 nothing up to 444	BOV_RS14405	ABQ62001.1	FAD		4AUT (34)
48	1.1.1.402	D-erythritol 1-phosphate dehydrogenase	DAO (PF01266) 8-333	BOV_RS14450 (eryB)	ABQ62056.1	FAD	Quinone	2QCU (51)
			DAO_C (PF16901) 386-482					
49	1.1.99.1	Choline dehydrogenase (GMC family, membrane bound)	GMC_oxred_N (PF00732) 5-295	BOV_RS14905	ABQ62100.1	FAD		4HA6 (34)
			GMC_oxred_C (PF05199) 387-523					
50	1.1.99.2	Predicted L-2-hydroxyglutarate dehydrogenase	DAO (PF01266) 5-392	BOV_RS15155 (lhgO)	ABQ62911.1	FAD	NADH	3DME (37)
51	1.1.99.14	Glycolate dehydrogenase GlcD subunit	FAD_binding_4 (PF01565) 55-193	BOV_RS11160 (glcD)	ABQ62237.1	FAD		3PM9 (29)
			FAD-oxidase_C (PF02913) 229-470					
52	1.3.1.-^*b*^ /1.7.1.B1	Predicted alkene reductase: N-ethylmaleimide reductase, glycerol trinitrate reductase or xenobiotic reductase B	Oxidored_FMN (PF00724) 3-349	BOV_RS14625	ABQ62490.1	FMN	NAD(P)H	5N6G (69)
53	1.3.8.-^*b*^	Acyl-CoA dehydrogenase	AidB_N (PF18158) 17-173	BOV_RS13205	ABQ62576.1	FAD		5EZ3 (100)
			Acyl-CoA_dh_M (PF02770) 188-282					
			Acyl-CoA_dh_1 (PF00441) 292-447					
54	1.3.8.-^*b*^	Acyl-CoA dehydrogenase	Acyl-CoA_dh_N (PF02771) 4-116	BOV_RS12330	ABQ62784.1	FAD		4N5F (63)
			Acyl-CoA_dh_M (PF02770) 120-214					
			Acyl-CoA_dh_1 (PF00441) 227-375					
55	1.3.8.-^*b*^	Acyl-CoA dehydrogenase	Acyl-CoA_dh_N (PF02771) 9-115	BOV_RS14135	ABQ62889.1	FAD		5LNX (36)
			Acyl-CoA_dh_M (PF02770) 120-218					
			Acyl-CoA_dh_1 (PF00441) 231-378					
56	1.3.8.-^*b*^	Acyl-CoA dehydrogenase	Acyl-CoA_dh_N (PF02771) 3-157	BOV_RS14115	ABQ62082.1	FAD		6IJC (61)
			Acyl-CoA_dh_M (PF02770) 163-271					
			Acyl-CoA_dh_1 (PF00441) 282-451					
			Acyl-CoA_dh_C (PF12806) 467-593					
57	1.3.99.-^*b*^	Predicted KsdD-like steroid dehydrogenase	FAD_binding_2 (PF00890) 5-533	BOV_RS13530	ABQ62061.1	FAD		1D4D (25)
58	1.4.1.13	Glutamate synthase large subunit (alpha subunit)	GATase_2 (PF00310) 56-480	BOV_RS10585 (gltB)	ABQ61996.1	FMN	3Fe-4S	1EA0 (45)
			Glu_syn_central (PF04898) 508-794					
			Glu_synthase (PF01645) 856-1230					
			GXGXG (PF01493) 1309-1498					
		Glutamate synthase small subunit (beta subunit)	Fer4_20 (PF14691) 24-131	BOV_RS10590 (gltD)	ABQ62546.1	FAD	NADPH 4Fe-4S	6S6U_G (37)
			Pyr_redox_2 (PF07992) 148-465					
59	1.4.99.-^*b*^	D-amino acid dehydrogenase	DAO (PF01266) 3-397	BOV_RS14735 (dadA)	ABQ62278.1	FAD		4YSH (26)
60	1.4.-.-^*b*^	Predicted D-amino acid dehydrogenase	DAO (PF01266) 7-397	BOV_RS13345	ABQ62519.1	FAD		4YSH (26)
61	1.4.-.-^*b*^	Predicted D-amino acid dehydrogenase	DAO (PF01266) 34-384	BOV_RS13970	ABQ62405.1	FAD		4YSH (24)
62	1.5.3.1	Predicted monomeric Sarcosine oxidase	DAO (PF01266) 31-383	BOV_RS13970	ABQ62932.1	FAD		1ZOV (22)
63	1.6.99.1	***NADPH dehydrogenase (Old yellow enzyme)***	Oxidored_FMN (PF00724) 2-340	BOV_RS11390	ABQ62422.1	FMN	NADPH	3GR7 (34)
64	1.6.-.-^*b*^	***NADH dehydrogenase***	Pyr_redox_2 (PF07992) 8-325	BOV_RS12460	ABQ62704.1	FAD	NADH	4NWZ (31)
65	1.8.1.2	Assimilatory sulphite reductase (NADPH) alpha component cluster	PepSY_TM (PF03929) (3-149)	BOV_RS11420	WP_006015252.1			TM helices
			Hypothetical protein (81aa)	BOV_RS11425	WP_006015255.1			
			PepSY_TM (PF03929) (2-128)	BOV_RS11430	WP_006015257.1			
			Flavodoxin_1 (PF00258) (74-147)			FMN		6EFV (∼30 B. melitensis)
			FAD_binding_6 (PF00970) (80-171)	BOV_RS11435	WP_006015259.1	FAD		
			NAD_binding_1 (PF00175) (189-291)				NADPH	
66	1.8.1.4	Dihydrolipoyl dehydrogenase (lpdA-1)	Pyr_redox_2 (PF07992) 7-326	BOV_RS12670 (lpdA-1)	ABQ62466.1	FAD	NAD(P)H	6CMZ (60)
			Pyr_redox_dim (PF02852) 345-453					
67	1.8.5.B1	Peptide-methionine (S)-S-oxide reductase (quinone) (Msr). MsrP catalytic subunit. MsrQ heme-binding subunit	Ferric_reduct (PF01794) 55-166	BOV_RS15075 (msrQ)	ABQ62365.1	FMN	heme b	6HCY_A (17)
			Oxidored_molyb (PF00174) 98-252	BOV_RS15070 (msrP)	ABQ62343.1	Molybdopterin	Quinone	1XDY (54)
68	1.13.11.32	Nitronate monooxygenase (formerly 2-nitropropane dioxygenase NPD)	NMO (PF03060) 124-342 (formerly NPD)	BOV_RS14290	ABQ62537.1	FMN		3BW2 (26)
69	1.13.11.79	Predicted aerobic 5,6-dimethylbenzimidazole synthase (BluB)	Nitroreductase (PF00881) 74-240	BOV_RS15390 (bluB)	ABQ62404.1	FMN	NADH	2ISK (36)
70	1.14.13.-^*b*^	UbiH/UbiF family hydroxylase	FAD_binding_3 (PF01494) 49-377	BOV_RS13080	ABQ62553.1	FAD	NAD(P)	5KOX_A (20)
71	1.14.13.2	4-hydroxybenzoate 3-monooxygenase	FAD_binding_3 (PF01494) 2-342	BOV_RS13400 (pobA)	ABQ62030.1	FAD	NAD(P)H	1PBE (63)
72	1.18.1.3/5	Predicted Ferredoxin/rubredoxin/ putidaredoxin NAD^+^ Reductase	1: Pyr_redox_2 (PF07992) 4-301	BOV_RS13795	ABQ62051.1	FAD	NADH	3FG2 (45)
			2: Reductase_C (PF14759) 320-404					
73	1.-.-.- ^*b*^	***Predicted nitroreductase family protein***	Nitroreductase (PF00881) 48-216	BOV_RS12545	ABQ62091.1	FMN		2IFA (55)
74	2.7.7.2	Bifunctional riboflavin kinase/FAD synthase	FAD_syn (PF06574) 18-172	BOV_RS11255 (ribF)	ABQ62831.1	FMN & FAD		2X0K (36)
	2.7.1.26		Flavokinase (PF01687) 190-313			RF & FMN		
75	2.7.1.180	FAD:protein FMN transferase	ApbE (PF02424) 18-296	BOV_RS11440	ABQ62066.1	FAD		5MGY (38) 6NXI (36)
76	2.7.13.3	Blue-light-activated histidine kinase	PAS_9 (PF13426) 34-136	BOV_RS13160	ABQ62113.1	FMN		6PPS (100)
			PAS_3 (PF08447) 184-259					
			HWE_HK (PF07536) 285-367					
77	4.1.1.36	Coenzyme A biosynthesis bifunctional protein: Phosphopantothenoyl-cysteine decarboxylase/Phosphopantothenate–cysteine ligase (CTP)	Flavoprotein (PF02441) 7-180	BOV_RS15430 (coaBC)	ABQ62036.1	FMN		1E20 (34)
	6.3.2.5		DFP (PF04127) 189-375				CTP	4QJI (42)
78		Protein NrdI	Flavodoxin_NdrI (PF07972) 5-122	BOV_RS11810 (nrdI)	ABQ62891.1	FMN		2XOD (36)

a Pfam domains include name, code, and residues in the B. ovis flavoprotein making the domain.

b Identified as flavoenzyme, but available information does not allow to fully predict its activity.

c Shown in bold and italics are those candidates for which a clear function cannot be depicted.

Fifty (64%) and 22 (28%) of these flavoproteins are predicted to bind, respectively, FAD and FMN as cofactor (Tables 1 and 2). Four (5%) would bind both: the NADH dependent dihydropyrimidine dehydrogenase, binding FMN and FAD respectively at its PreA and PreT subunits; the sarcosine oxidase beta subunit; the glutamate synthase, binding FMN and FAD respectively at the large and short subunits; and the assimilatory sulfite reductase (NADPH) alpha component cluster where FMN and FAD bind respectively at two of its subunits. The bifunctional riboflavin kinase/FAD synthase would bind RF, FMN, and FAD, and the riboflavin synthase alpha subunit would bind RF as product. Despite the lower content of proteins binding FMN, the B. ovis flavoproteome is slightly biased toward FMN when considering overall kingdoms, where the majority of flavoenzymes bind FAD (75%) (34), or some eukaryotic flavoproteomes, as the Homo sapiens one (with 84% FAD-dependent proteins) (6, 38). Thirty-three of the identified flavoproteins in B. ovis are expected to use NAD(P)^+^/H as coenzyme, whereas a few would bind haem, iron-sulfur clusters, quinones, CTP, or thiamine phosphate. It is worth mentioning that currently many of these proteins are only computationally annotated and, in many cases, with vague confidence regarding ligands (searching motifs for FAD also find NAD(P)H binding proteins), nature of the protein, and metabolic functions.

### The structure conformational space in the B. ovis flavoproteome.

3D structures of proteins from B. ovis are scarce: 10 different proteins in 14 Protein Data Bank (PDB) entries (Table SP2). Only one corresponds to a flavoprotein that is also NADP^+^/H dependent, Ferredoxin-NADP^+^ reductase (FPR) (39), and three more correspond to structures, or subunits, of NAD(P)^+^/H dependent proteins (Table SP2). The PDB contains also 11 structures of potential flavoproteins from other Brucella (Table SP3 and Fig. SP2). Ten correspond to nine B. ovis homologues sharing more than 98.5% identity, and two are also NAD(P)^+^/H dependent. For the remaining structure, the best match in B. ovis ATCC 25840 only shares 33% identity, but an identical sequence is found in B. ovis IntaBari-2002-82-58. Five of these flavoproteins are FAD-dependent (only two show FAD in the structure), three are FMN-dependent, and one might bind either RF, FMN, or FAD (Table SP3). Regarding function, seven are oxidoreductases with NAD(P)^+^/H (quinone) dehydrogenase, acyl-CoA dehydrogenase, thioredoxin reductase, or monooxygenase activities. Three are transferases; one is riboflavin synthase and two relate to the LOV domain of sensory histidine kinase. Notably, the PDB entry 3U0I from Brucella melitensis, identified as a pyridoxamine 5′-phosphate oxidase family protein of unknown function, shares nearly 100% identity with B. ovis ABQ61684.1. Most of these structures come from structural genomic projects on B. melitensis, Brucella abortus, and Brucella suis, and their functions are not experimentally curated. Tables 1 and 2 show that in 66 (85%) and 39 (50%) of the candidates there are structures of homologues with more than 30% and 45% sequence identity, respectively, providing good structural models for at least ∼50% of the flavoproteins.

**FIG 1:**
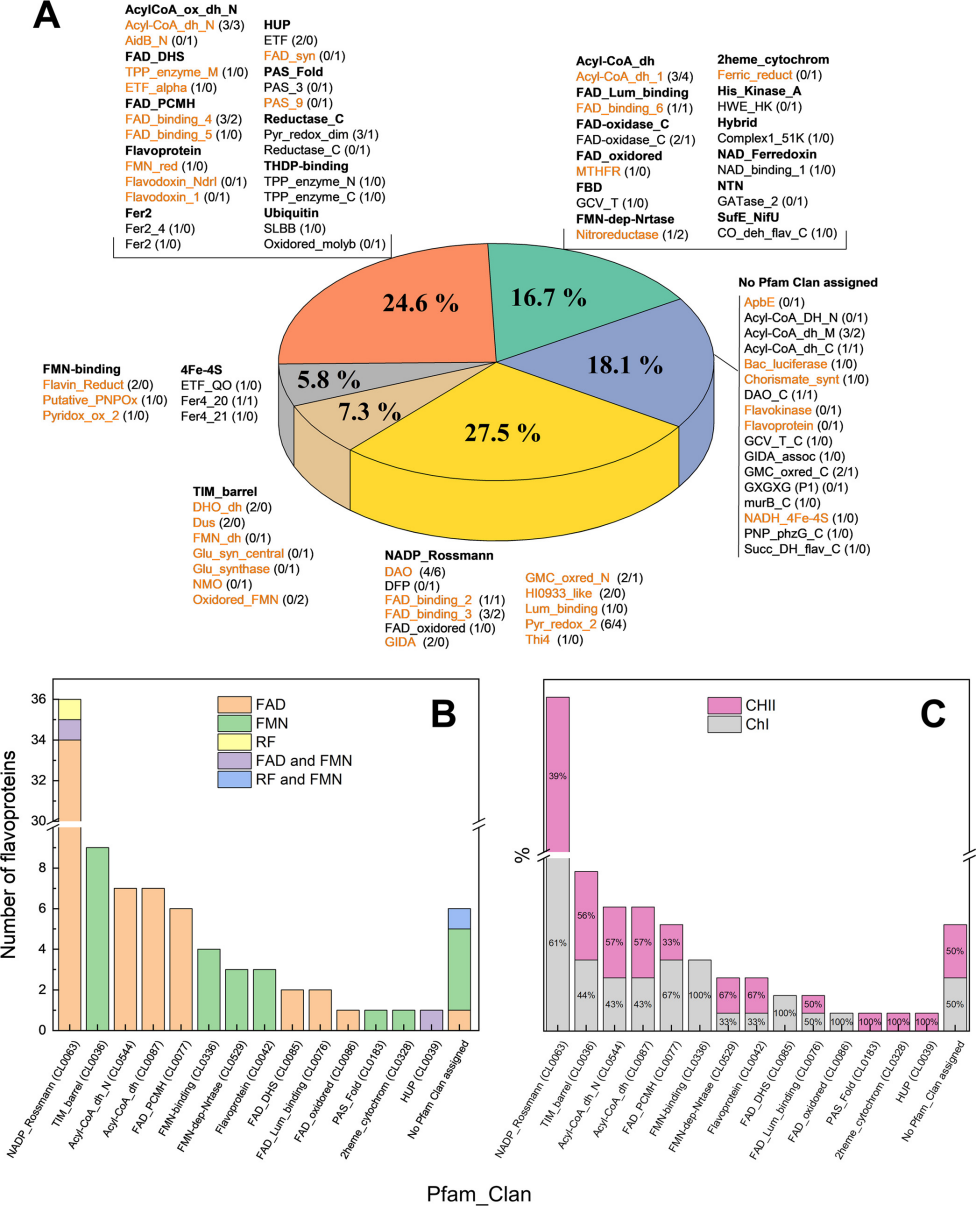
Distribution of B. ovis ATCC 25840 flavoproteins in structural Pfam clans and domain families. (A) Pie chart of the clans and domains found within the identified flavoproteins. Clan names are highlighted in bold. Names of domain families directly involved in flavin binding are colored in orange, whereas domains not involved in flavin binding but present in the flavoproteins are in black. Overall percentages are based on the number of domain families in each clan (including domains involved and not involved in flavin binding). Most populated clans concerning structural folding, NADP_Rossman and TIM_barrel, are shown individually, while the rest are grouped according to the number of families found in each: three (gray), two (orange), or one (green). The blue portion includes domain families with no clan assigned. The number for a particular domain presented in each chromosome is denoted in brackets, as *N* in CI/*N* and CII/*N*. Details for only flavin binding domains are shown in Figure SP1. (B) Clans involved in flavin cofactor binding according to the flavin type. (C) Distribution of flavin binding clans by chromosomal location.

**FIG 2:**
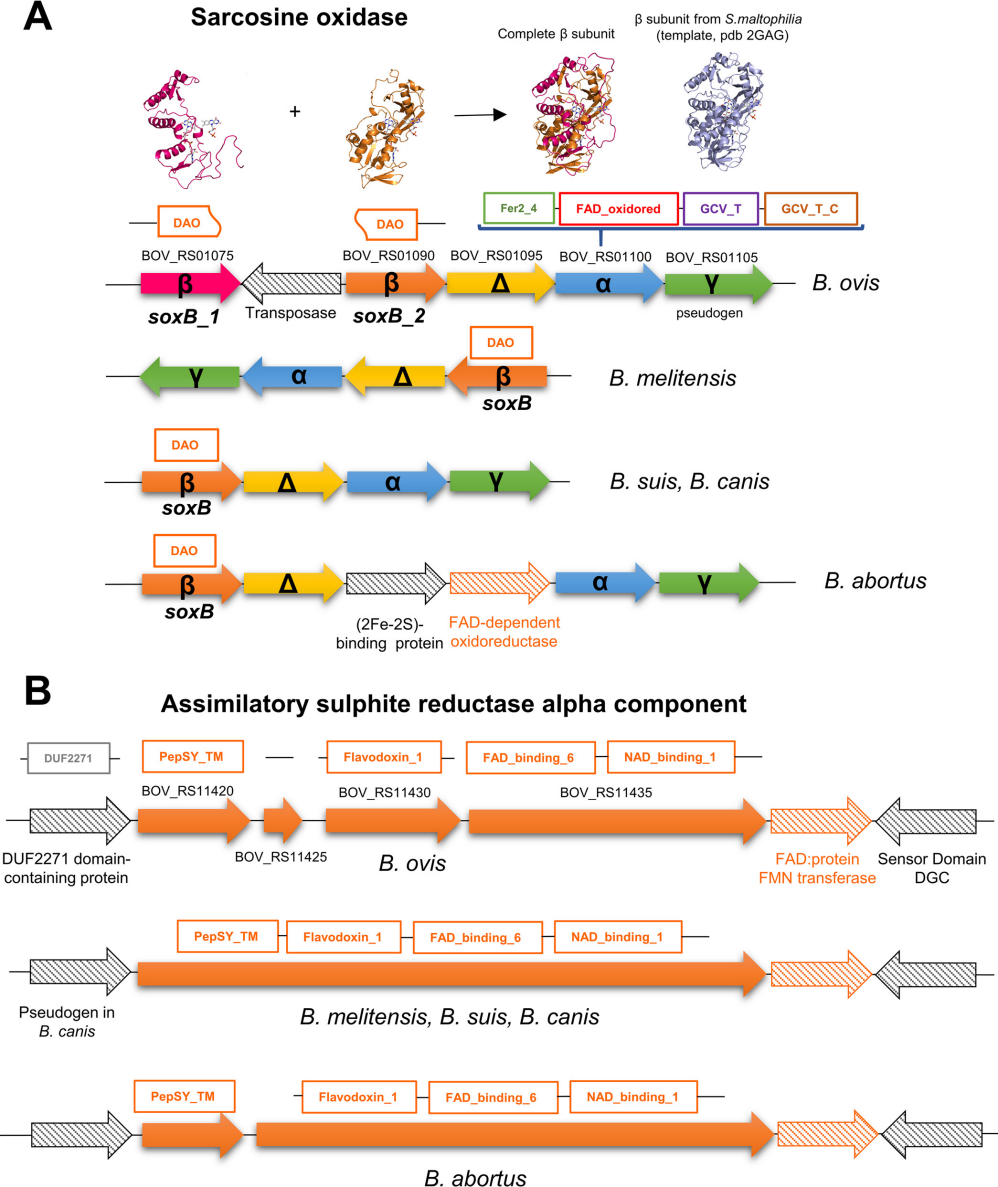
Genomic context for *SoxB* and sulfite reductase component genes in selected Brucella species. (A) Organization of genes encoding different subunits of the membrane bound sarcosine oxidase subunit B (SoxB). In B. ovis, two genes, *soxB_1* and *soxB_2*, separated by a IS5 transposase gene, encode together the full length of the SoxB protein. On the top, the homology structural models of SoxB_1 and SoxB_2 proteins and their superposition on the SoxB from Stenotrophomonas maltophilia (2GAG_B) are shown. (B) Organization of genes encoding for the assimilatory sulfite reductase alpha component. In both panels, gene senses are denoted by arrows and B. ovis gene codes are written next to the corresponding arrow. Structural Pfam domain families for subunits contributing to flavin binding are shown on the top of genes.

B. ovis flavoproteins use up to 26 Pfam clans and 73 domain families (Tables 1 and 2, Fig. 1, and Fig. SP1), and in 80% of the cases they fold using more than one domain. Nonetheless, only 13 clans, plus six domains not assigned to any clan, are implicated in flavin binding (Fig. SP1), in agreement with the diversity in structural topologies for the interaction of these cofactors. The NADP_Rossmann and TIM-barrel clans are the most widely represented and preferred respectively for FAD and FMN binding in B. ovis flavoproteins, being highly spread in proteins and particularly within flavoproteins (5, 7). The NADP_Rossmann fold appears in 38 flavoproteins in B. ovis, but presumably it is not involved in flavin binding in domains FAD_oxidored of sarcosine oxidase alpha subunit and DFP of coenzyme A biosynthesis bifunctional protein, where it respectively binds NADH and CTP. From the 36 remaining flavoproteins, 35 bind FAD and riboflavin synthase binds RF (Lum_binding domain). NADP_Rossmann flavoproteins use mostly domains DAO (some completed by DAO_C domain caps) and Pyr_redox_2 (might contain also Pyr_redox_dim or Fer4_20 domains). Domain families FAD_binding_3, GMC_oxired_N (also having GMC_oxred_C), FAD_binding_2 (some completed with Succ_DH_flav_C), GIDA (holding also GIDA_assoc), HI0933_like, and Thi4 are also represented. In this clan we want to notice a particular case: two proteins with DAO domains shorter than regular ones, ABQ60177.1 (BOV_RS01075, *soxB_1*) and ABQ61310.1 (BOV_RS01090, *soxB_2*), cover the primary sequence of the sarcosine oxidase beta subunit binding FAD in other Brucella orthologues (Table 1, Fig. 2A). On its side, the TIM_barrel clan is represented by nine flavoproteins that use up to seven different domains: Oxidored_FMN, Dus, DHO_dh, FMN_dh, Glu_syn_central, Glu-synthase, and NMO.

Clans AcylCoA_ox_dh_N and AcylCoA_dh appear in seven FAD dependent acyl-CoA dehydrogenases. Normally, they fold in Acyl-CoA_dh_N (in one case replaced by Aidb_N) and Acyl-CoA_dh_1 domains and use connecting Acyl-Coa_dh_M and/or ending Acyl-CoA_dh_C domains. The six flavoproteins of the FAD_PCMH clan bind FAD; five and one use, respectively, FAD_binding_4 and FAD_binding_5 domains (completed with FAD-oxidase_C, CO_deh_flav_C, or murB_C domains).

The FMN-binding clan is predicted in four flavoproteins that bind FMN with Putative_PNPOx (with PNP_phzG_C domain), Flavin_Reduct, and Pyridox_ox_2 domains. Three also FMN binding flavoproteins fall in each of the FMN-dep-Nrtase and Flavoprotein clans. The first have nitroreductase domains, presumably involved in reduction of nitrogen-containing compounds, whereas those in the second clan belong each to a different family: FMN_red (NAD(P)H-quinone dehydrogenase), Flavodoxin_NdrI (electron transport NrdI protein), and Flavodoxin_1 (assimilatory sulfite reductase alpha component). This subunit of assimilatory sulfite reductase contains also a FAD_binding_6 domain, of the FAD_Lum_binding clan, that binds FAD. A FAD_binding_6 domain is also present in ferredoxin-NADP^+^ reductase. The assimilatory sulfite reductase alpha component in B. ovis shows noticeable features (Fig. 2B). In the current genome assembly, it is annotated as codified by four sequential genes (BOV_RS11420, BOV_RS11425, BOV_RS11430, and BOV_RS11435), which would made up four protein subunits (WP_006015252.1, WP_006015255.1, WP_006015257.1, and WP_006015259.1), whereas in other Brucella all these components are encoded by either one or two genes (Fig. 2B). Its central Flavodoxin_1 and FAD_binding_6 domains allocate FMN and FAD, respectively, whereas the additional domains will attach the protein to the membrane and bind the NADPH coenzyme. The functional protein will be complemented with a sulfite reductase (NADPH) haemoprotein beta-component (ABQ61351) codified in CI.

Only two B. ovis flavoproteins are members of the FADS_DHS clan. They adopt a Rossmann fold similar to clan NADP_Rossmann, but are distinguished since the FAD cofactor binds in the opposite direction. It is represented by the TPP_enzyme_M domain in the catalytic subunit of a synthase and ETF_alpha domain in the electron transferring flavoprotein alpha subunit (ETFa). Four clans are only found once in the B. ovis flavoproteome: the FAD_oxidored in the FAD dependent MTHFR domain of methylenetetrahydrofolate reductase; the PAS_Fold with the PAS_9 domain that binds FMN in the blue-light-activated histidine kinase; the HUP that binds FMN/FAD in the FAD_syn domain of bifunctional FAD synthase/flavokinase (FADS); and the 2heme_cytochrom binding FMN at the Ferric_reduct domain of MsrQ subunit of peptide-methionine (S)-S-oxide reductase (Msr).

Six domains not assigned to any clan also bind flavins in B. ovis flavoproteins: the Flavokinase domain binds RF/FMN in bifunctional FADS; the Bac_luciferase domain binds FMN in bacterial luciferase; the Chorismate_synt domain binds FMN in chorismate synthase; the NADH_4Fe_4S domain binds FMN at the NADH-quinone oxidoreductase subunit F; the ApbE domain binds FAD in the FAD:protein FMN transferase; and the Flavoprotein domain binds FMN in Coenzyme A biosynthesis bifunctional protein.

In agreement with the use of FAD-dependent enzymes for novel or unusual functions requiring the adaptation of already existing topologies or new structural designs (7), the less populated clans and domains in the B. ovis flavoproteome mainly contribute to FMN binding.

**FIG 3:**
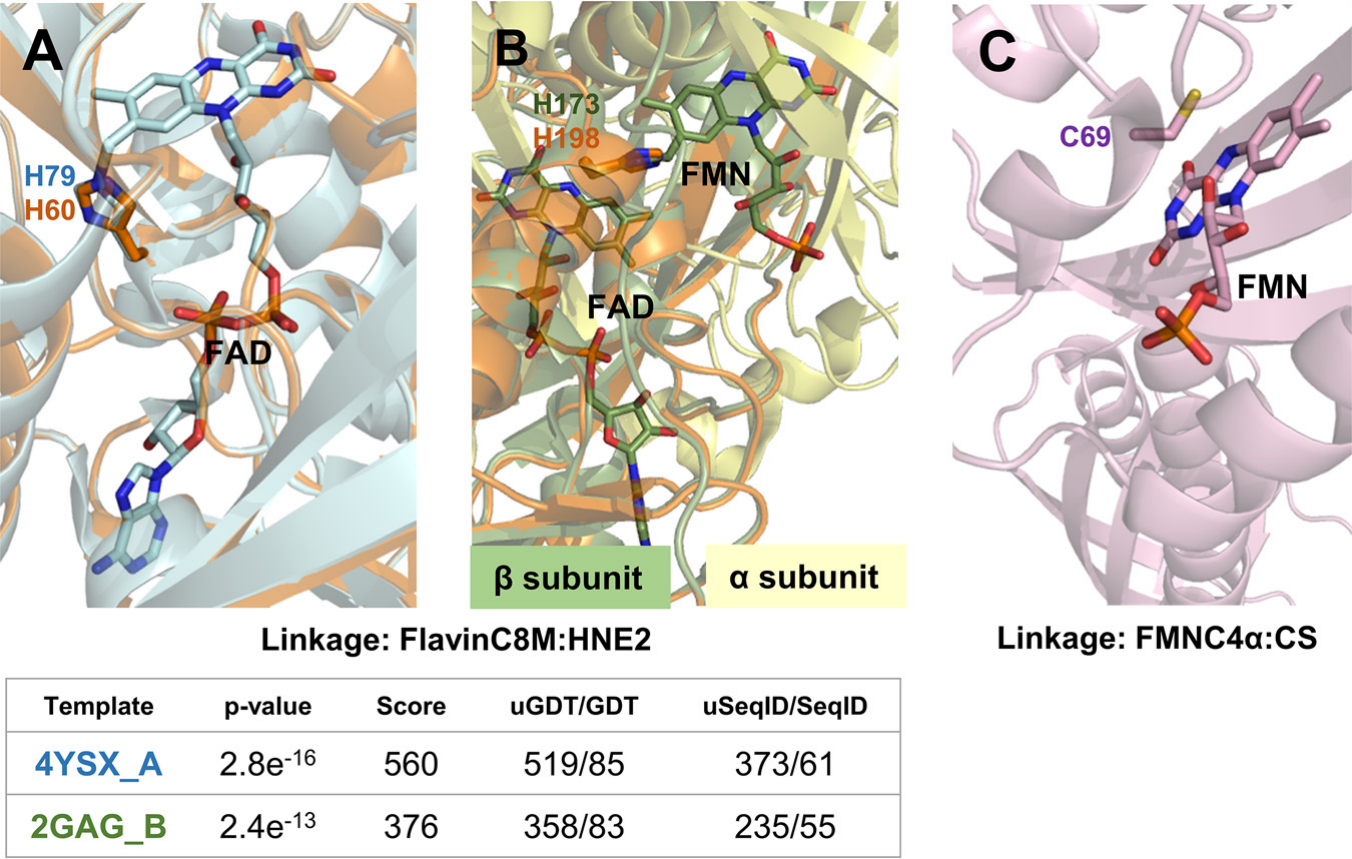
Structural models for B. ovis enzymes predicted to covalently bind the flavin cofactor. (A) succinate dehydrogenase flavoprotein subunit and (B) sarcosine oxidase beta subunit models (orange) for the covalent linking to C8M of flavins in B. ovis. Homology models were built using as templates the structures of *Ascaris suum* (PDB 4YSX_A, light blue in A) and S. maltophilia (2GAG, green in B), respectively, and the RaptorX server. Parameters predicting models’ quality are summarized below the figure. (C) Structure of the blue-light-activated histidine kinase from Brucella abortus *2308* (6PPS), with 100% identity to the B. ovis enzyme. Relevant residues as well as cofactors belonging to the templates are shown in sticks. Images generated with PyMol (134).

From the 78 identified candidates, only two flavoproteins are envisaged to bind flavins covalently. One is the succinate dehydrogenase flavoprotein subunit (SdhA), where covalent binding through H60 (in the conserved FPTRS**H**TV motif) to the FAD isoalloxazine (C8M) is predicted, as observed for H45, H56, or H79 in E. coli (PDB 2WDQ), Gallus Sudha (PDB 2H88), or *Ascaris suum* (PDB 4YSX) proteins (Fig. 3A). Covalent attachment of FAD to SdhA is essential for Sdh function in other bacteria (19). Covalent linking to FMN (C8M) is also predicted in the sarcosine oxidase beta subunit through H198 (H173 in the Stenotrophomonas maltophilia protein, PDB 2GAG), placing FMN at the alpha and beta subunits interface (Fig. 3B). Sequence and structural modeling also point to blue-light-activated histidine kinase as undergoing photochemistry with cysteinyl-flavin adduct formation between C69 and the isoalloxazine C4a of FMN (Fig. 3C), altogether these observations point out that the cofactor is noncovalently bound in most B. ovis flavoproteins.

### Enzymatic classification and metabolic functions of Brucella ovis flavoproteins.

**FIG 4:**
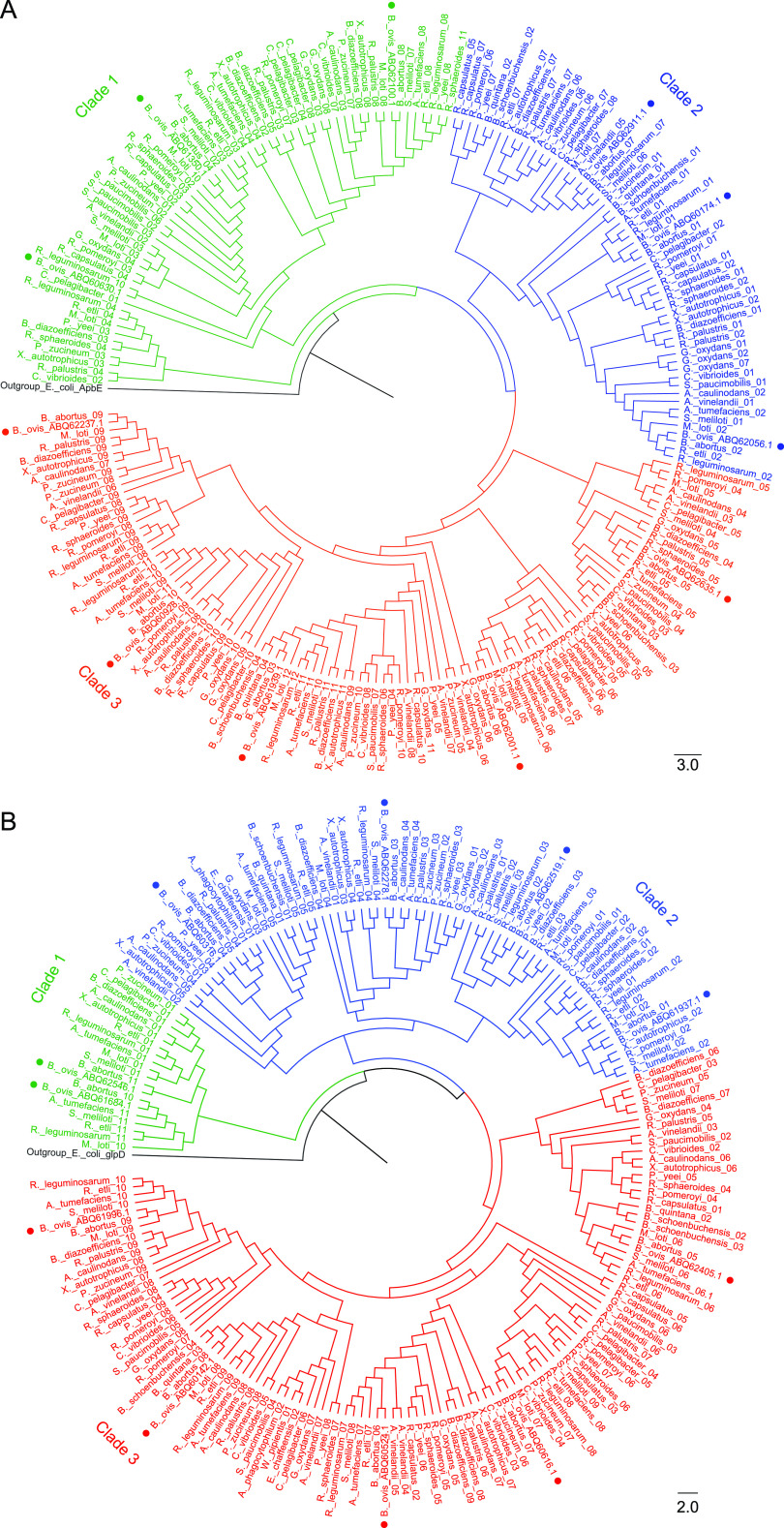
Phylogeny of B. ovis flavoproteins of subclasses (A) EC 1.1. and (B) EC 1.4. within alphaproteobacteria. (A) Subclass EC 1.1. distributes in three phylogenetic clades (bootstrap >92). Clade 1 clusters (bootstrap >90) three proteins of the GMC family, ABQ61350.1, ABQ60630.1, and ABQ62100.1 (EC. 1.1.99.1). Clade 2 groups (bootstrap > 95) three proteins folding in DAO domains, ABQ60174.1, ABQ62056.1, and ABQ62911.1. Clade 3 clusters five proteins distributed in monophyletic branches ABQ61939.1, ABQ62001.1, ABQ62237.1, ABQ60928.1, and ABQ62635.1 with diverse functions (bootstrap >85). (B) Flavoenzymes of subclass EC 1.4. distribute in three clades (bootstrap >90). Clade 1 includes ABQ61684.1 and ABQ62546.1, present in a reduced number of alphaproteobacteria. Clade 2 comprises four proteins distributed in two subclades (bootstrap >88). ABQ60316.1 clusters separately (bootstrap >92), while ABQ62278.1, ABQ62519.1, and ABQ61937.1 are in the same subclade (bootstrap > 77). Clade 3 clusters ABQ62405.1 in a divergent subclade (bootstrap > 80), and ABQ60142.1, ABQ60524.1, ABQ60616.1, and ABQ61996.1, separately in four branches of other subclades (bootstrap > 80). Phylogenetic cladograms include 222 sequences of subclass EC 1.1. and 198 sequences of subclass EC 1.4. from B. ovis ATCC 25840, B. abortus 2308, and 26 alphaproteobacteria related species. E. coli ApbE and E. coli GlpD were selected as outgroup in A and B, respectively, to highlight the clear evolutionary separation between clusters. The likelihood aLRT (approximate likelihood-ratio test) statistical test and a bootstrap value of 100 were used.

**FIG 5:**
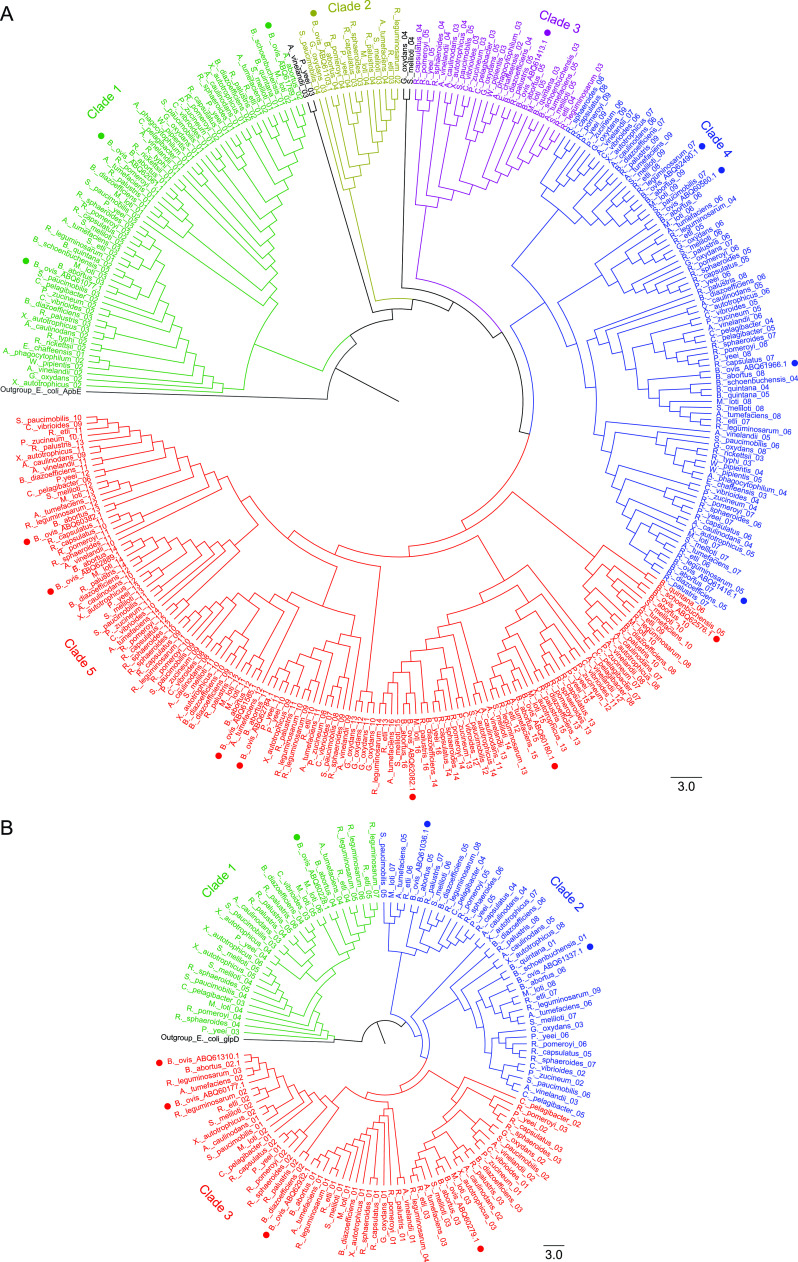
Phylogeny of B. ovis flavoproteins from subclasses (A) EC 1.3 and (B) EC 1.5. within alphaproteobacteria. (A) Flavoenzymes of subclass EC 1.3. separate in five clades and are relatively conserved regarding evolution. Clade 1 groups ABQ62061.1 and ABQ61769.1 together and ABQ61077.1 separately (bootstrap >98). Clade 2 (bootstrap >73) and clade 3 (bootstrap >70) contain, respectively, ABQ61103.1 and ABQ61413.1, given that these proteins are less conserved within studied species. Clade 4 distributes in four subgroups (bootstrap >95) including ABQ62490.1, ABQ60560.1, ABQ61416.1, and ABQ61966.1. Clade 5 includes seven ACAD homologues distributed in four subclades (bootstrap >90). The most divergent contains the entry ABQ62576.1 (bootstrap >98). (B) Flavoreductases of the subclass E.C 1.5 distribute in three clades (bootstrap >90). Clade 1 is the most divergent (bootstrap >98) and contains ABQ60228.1. Clade 2 separates in two branches (bootstrap >90) ABQ61036.1 and ABQ61337.1 homologues. Clade 3 distributes in two subclades (bootstrap >85). One subclade includes ABQ61310.1 and ABQ60177.1 grouped in the same branch and ABQ62932.1 in a separate branch, whereas the protein ABQ60279.1 clusters separately. Phylogenetic cladograms include 319 sequences of subclass EC 1.3. and 108 sequences of subclass EC 1.5. from B. ovis ATCC 25480, B. abortus 2308, and 26 alphaproteobacteria related species. E. coli ApbE and E. coli GlpD were selected as outgroup in A and B, respectively, to highlight the clear evolutionary separation between clusters. The likelihood aLRT (approximate likelihood-ratio test) statistical test and a bootstrap value of 100 were used.

**FIG 6:**
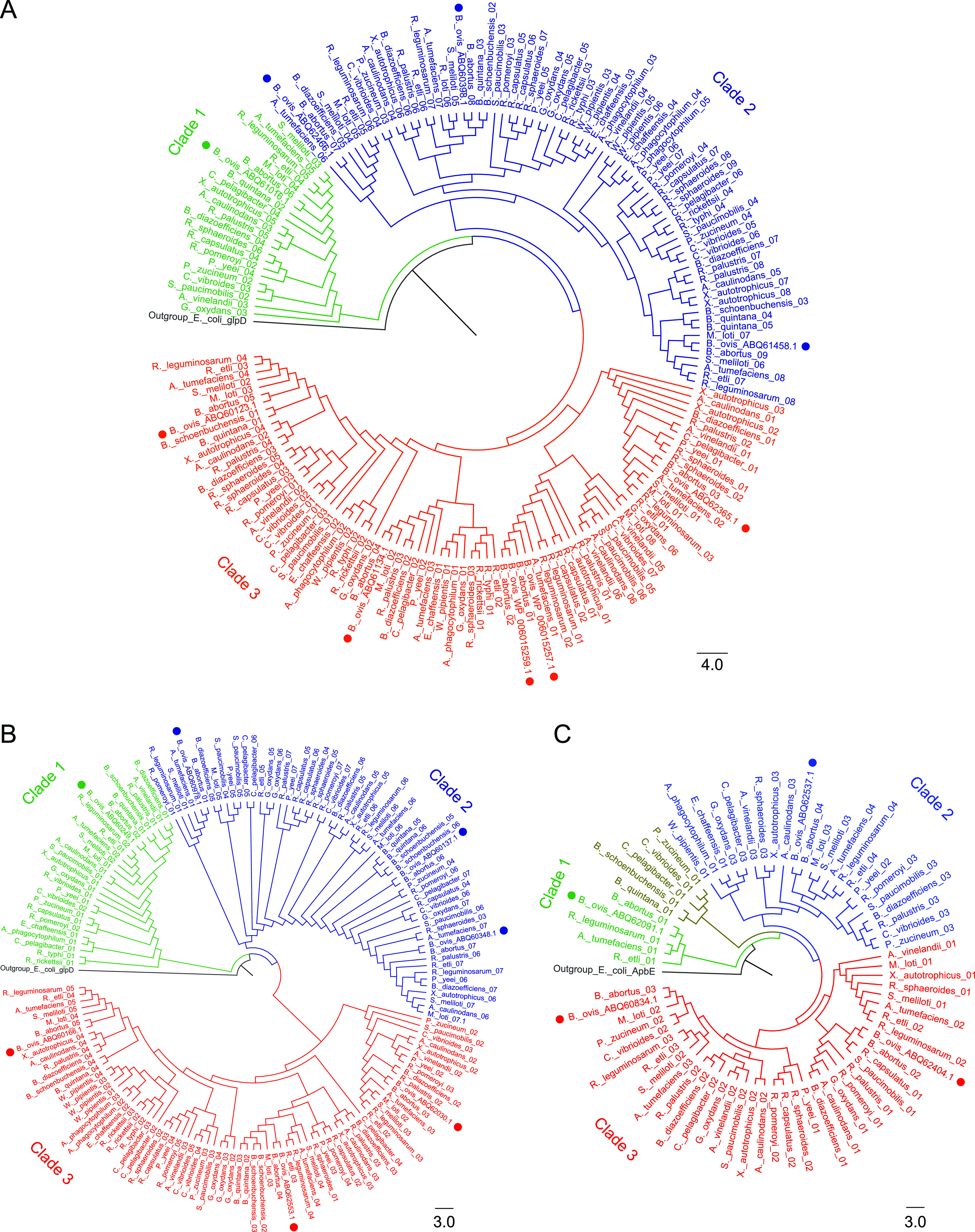
Phylogeny of B. ovis flavoproteins of subclasses (A) EC 1.8, (B) EC 1.14, and (C) EC 1.16 related to nitrogen metabolism within alphaproteobacteria. (A) Flavoenzymes of subclass EC 1.8. cluster in three clades (bootstrap >90). Clade 1 contains ABQ61016.1. Clade 2 distributes in two subclades organized in different subgroups that contain ABQ62466.1, ABQ60398.1, and ABQ61458.1. Clade 3 clusters in two subclades (bootstrap >91). The first subclade separates in two branches, WP_006015257.1 and WP_006015259.1 together and ABQ62365.1 separately. The second subclade clusters ABQ60123.1 and ABQ61134.1. (B) Flavoenzymes of subclass EC 1.14. and 1.16 distribute in three clades (bootstrap >80). Clade 1 includes the most divergent member, ABQ60249.1, which is the single member of the subclass 1.16. Clade 2 clusters ABQ60137.1, ABQ60978.1, and ABQ60348.1 (bootstrap >90). Clade 3 has three subclades and is more diverse. The most divergent subgroup includes ABQ62030.1 (bootstrap > 80), while ABQ60166.1 and ABQ62553.1 cluster in two branches of the other subclade (bootstrap >96). (C) FMN dependent flavoreductases predicted to act on nitrogenous compounds separately in three clades. Clade 1 has ABQ62091.1, which is barely conserved in alphaproteobacteria. Clade 2 contains ABQ62537.1, and clade 3 separates in two branches, ABQ62404.1 and ABQ60834.1 (bootstrap >75). Phylogenetic cladograms include 177 sequences of class EC 1.8., 160 sequences of class EC 1.14./1.16. and 71 sequences of nitrogen metabolism from B. ovis ATCC 25840, B. abortus 2308, and 26 alphaproteobacteria related species. E. coli GlpD and E. coli ApbE were selected as outgroup in A, B, and C, respectively, to highlight the clear evolutionary separation between clusters. The likelihood aLRT (approximate likelihood-ratio test) statistical test and a bootstrap value of 100 were used.

**FIG 7:**
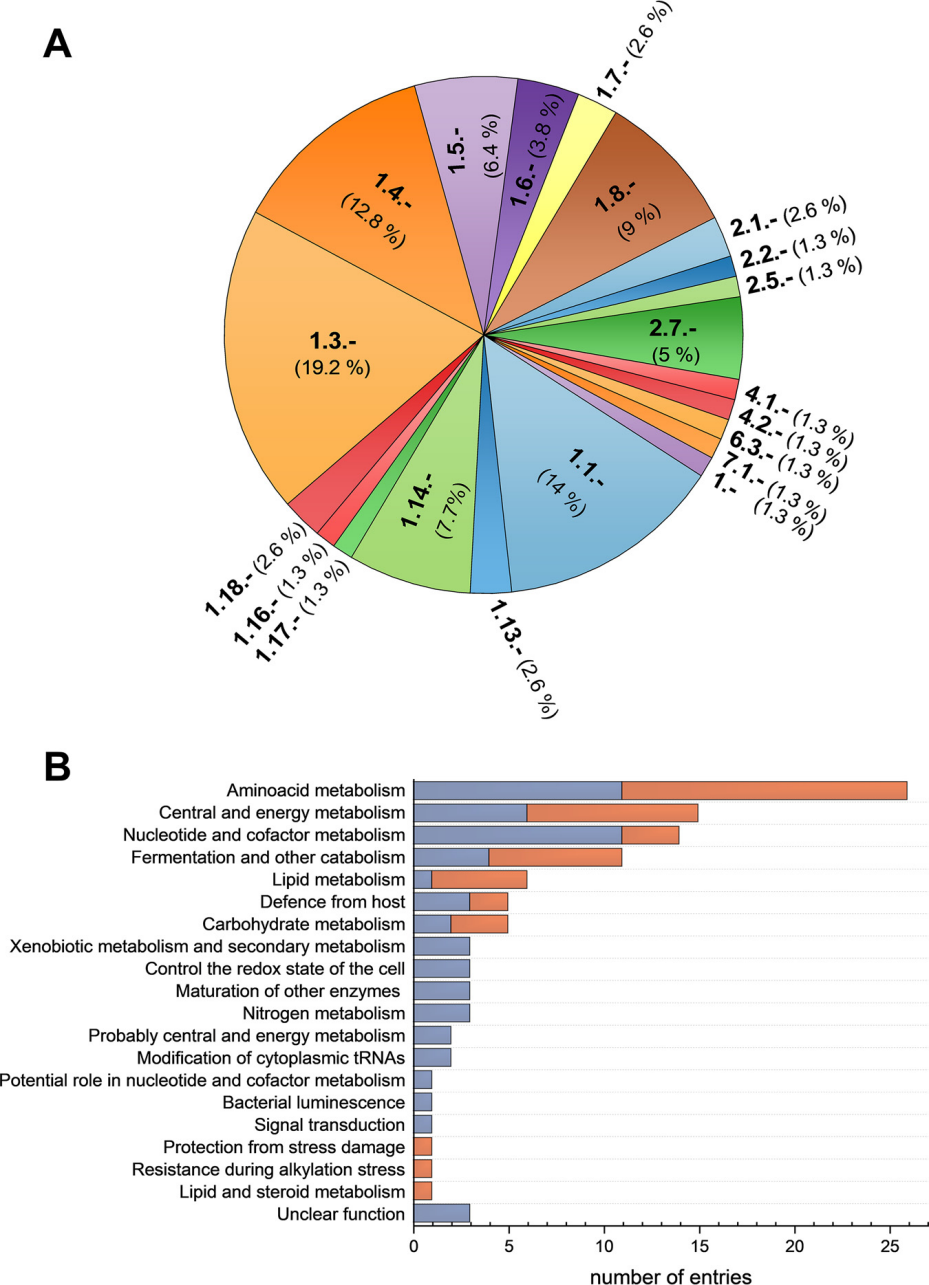
Metabolic functions for the B. ovis flavoproteins. (A) Pie chart distribution of ECs. (B) Number of flavoproteins involved in different metabolic pathways. Blue portions of bars relate to entries predicted to act in a single pathway, whereas orange ones represent entries acting in several pathways.

Most proteins of the B. ovis flavoproteome (76 out of 78) are flavoenzymes. We assigned enzymatic classes to all of them and full Enzyme Commission (EC) codes to nearly 70% (Table 3, Fig. 4, 5 and 6). The rest (30%) might have either divergent mechanisms from known flavoproteins or still not reported full functions. Flavoenzymes fall into the oxidoreductases (EC 1.) (86%, 66 out of 76), transferases (EC 2.) (9.2%, 7 out of 76, one of them with two transferase activities), lyases (EC 4.) (2.6%, 2 out of 76), and translocases (EC 7.) (1.3%, 1 out of 76) classes (Tables 1 and 2, and Table SP6; Fig. 7A). Therefore, most of B. ovis flavoenzymes participate in redox processes, in agreement with previous reported classifications (5, 7).

**TABLE 3:** Metabolic functions and virulence potential envisaged for flavoproteins and flavoenzymes from B. ovis ATCC 25840. Search for metabolic functions and virulence prediction carried out as indicated in Materials and Methods

EC	Protein	Protein code	Metabolic pathway	Pathway category	VirulentPred ^*b*^	VICMpred function^*c*^
1.1.5.3	Glycerol-3-phosphate dehydrogenase	ABQ60174.1	Degradation of sugar alcohols	Carbohydrate metabolism	Non-virulent	Metabolism
1.1.1.402	D-erythritol 1-phosphate dehydrogenase	ABQ62056.1	Degradation of sugar alcohols	Carbohydrate metabolism. **Virulence factor.**	Non-virulent	Metabolism
1.1.2.3	L-lactate dehydrogenase (cytochrome c or b2)	ABQ62635.1	Lactate fermentation	Fermentation and other catabolism	Non-virulent	Metabolism
1.1.3.8	***Potential L-gulonolactone oxidase FAD-binding oxygen oxidoreductase* ^*a*^**	ABQ62001.1	Unclear function. Potentially involved in ascorbate metabolism	Potential role in nucleotide and cofactor metabolism	Non-virulent	Cellular Process
1.1.99.1	Choline dehydrogenase (Glucose-methanol-choline GMC family)	ABQ61350.1	Glycine betaine biosynthesis. Metabolism of disaccharides	Amino acid Metabolism. Carbohydrate metabolism	Non-virulent	**Virulence Factor**
1.1.99.1	Choline dehydrogenase (GMC family)	ABQ60630.1			**Virulent**	Cellular Process
1.1.99.1	Choline dehydrogenase (GMC family, membrane bound)	ABQ62100.1			Non-virulent	Metabolism / **Potential Virulence Factor**
1.1.99.2	Predicted L-2-hydroxyglutarate dehydrogenase	ABQ62911.1	Glutamate and glutamine metabolism	Amino acid metabolism	**Virulent**	Metabolism
1.1.99.14	Glycolate dehydrogenase GlcD subunit	ABQ62237.1	Glycolate and Glyoxylate degradation	Central and energy metabolism	Non-virulent	Metabolism
1.1.-.-	***Potential FAD-binding oxygen oxidoreductase (glcE?)***	ABQ60928.1	It might work with GlcD	Probably central and energy metabolism	Non-virulent	Metabolism
1.1-.-	***Potential FAD-binding oxygen oxidoreductase (glcE?)***	ABQ61939.1	It might work with GlcD	Probably central and energy metabolism	Non-virulent	Metabolism
1.3.1.1/2	NADH dependent Dihydropyrimidine dehydrogenase subunit PreA	ABQ60560.1	Pyrimidine and alanine metabolism	Nucleotide and cofactor metabolism. Amino acid metabolism	Non-virulent	Cellular Process / Metabolism
	NADH dependent Dihydropyrimidine dehydrogenase subunit PreT	ABQ61103.1			Non-virulent	Cellular Process / Metabolism
1.3.5.2	PyrD dihydroorotate dehydrogenase 2 (quinone)	ABQ61413.1	Pyrimidine Metabolism	Nucleotide and cofactor metabolism	Non-virulent	Metabolism
1.3.1.88	tRNA dihydrouridine synthase B	ABQ61416.1	Dihydrouridine modification of tRNA	Modification of cytoplasmic tRNAs	Non-virulent	Metabolism
1.3.1.91	tRNA dihydrouridine20/20a synthase	ABQ61966.1			Non-virulent	Metabolism
1.3.1.98	UDP-N-acetylmuramate dehydrogenase	ABQ61769.1	Peptidoglycan Biosynthesis. Cell wall biogenesis	Amino acid metabolism	**Virulent**	**Virulence Factor**
1.3.1.- /1.7.1.B1	Predicted alkene reductase: N-ethylmaleimide reductase, Glycerol trinitrate reductase or xenobiotic reductase B	ABQ62490.1	Degradation of toxic compounds	Xenobiotic metabolism and secondary metabolism	Non-virulent	Metabolism
1.3.5.1	Succinate dehydrogenase flavoprotein subunit	ABQ61077.1	Citric acid and methylaspartate cycles. Propionate fermentation	Fermentation and other catabolism. Central and energy metabolism. **Virulence factor**	Non-virulent	**Virulence Factor**
1.3.8.4	Isovaleryl-CoA dehydrogenase	ABQ60382.1	Leucine metabolism	Amino acid metabolism	**Virulent**	Cellular Process
1.3.8.-	Short Chain Acyl-CoA dehydrogenase	ABQ60180.1	Lipid metabolism. Butanone fermentation. Alanine, glutamate and glutamine metabolism. Ethylmalonyl-CoA pathway	Lipid metabolism. Fermentation and other catabolism. Amino acid metabolism. Central and energy metabolism	Non-virulent	Cellular Process
1.3.8.1	Acyl-CoA dehydrogenase	ABQ62576.1	Bacterial resistance during alkylation stress/Cell division/ AidB domains	Lipid and steroid metabolism. Resistance during alkylation stress. Defence from Host	**Virulent**	Metabolism
1.3.8.-	Acyl-CoA dehydrogenase	ABQ61585.1	Lipid metabolism. Butanone fermentation. Valine, alanine, tryptophan, glutamate and glutamine metabolisms. Ethylmalonyl-CoA pathway. Phenyl acetate degradation (aerobic). Cyclohexanol degradation. Adipate degradation	Lipid metabolism, Fermentation and other catabolism, Amino acid metabolism, Central and energy metabolism.	Non-virulent	Metabolism
1.3.8.-	Acyl-CoA dehydrogenase	ABQ62784.1			Non-virulent	**Virulence Factor**
1.3.8.-	Acyl-CoA dehydrogenase	ABQ62889.1			Non-virulent	**Virulence Factor**
1.3.8.-	Acyl-CoA dehydrogenase	ABQ62082.1			Non-virulent	Metabolism
1.3.99.-	Predicted KsdD-like steroid dehydrogenase	ABQ62061.1	Androgen and steroid metabolism	Lipid metabolism	Non-virulent	Metabolism
1.4.1.13	Glutamate synthase large subunit (alpha subunit)	ABQ61996.1	Glutamate and glutamine metabolism	Amino acid metabolism	Non-virulent	Metabolism
	Glutamate synthase small subunit (beta subunit)	ABQ62546.1				
1.4.3.5	Pyridoxamine 5′-phosphate oxidase	ABQ60142.1	Vitamin B6 metabolism	Nucleotide and cofactor metabolism	Non-virulent	Cellular Process / Metabolism
1.4.-.-	***Pyridoxamine 5′-phosphate oxidase family protein***	ABQ61684.1	Unclear function	Unclear function	**Virulent**	Cellular Process / Metabolism
1.4.3.19	Glycine oxidase ThiO	ABQ60316.1	Thiamine metabolism	Amino acid metabolism	Non-virulent	Metabolism
1.4.3.-	***Potential Aminoacetone oxidase family FAD-binding enzyme/ NAD(P)/FAD-dependent dehydrogenase***	ABQ60616.1	Potential role in amino acids and NAD metabolism	Amino acid metabolism. Nucleotide and cofactor metabolism	Non-virulent	**Virulence Factor**
1.4.3.-	***Potential Aminoacetone oxidase family FAD-binding enzyme/ NAD(P)/FAD-dependent dehydrogenase***	ABQ60524.1	Potential role in amino acids and NAD metabolism	Amino acid metabolism. Nucleotide and cofactor metabolism	Non-virulent	Metabolism
1.4.99.-	Predicted D-amino acid dehydrogenase small subunit	ABQ61937.1	Potential role in oxidative deamination of D-amino acids	Amino acid metabolism	Non-virulent	**Virulence Factor**
1.4.99.-	D-amino acid dehydrogenase	ABQ62278.1			**Virulent**	Metabolism / **Potential Virulence Factor**
1.4.-.-	Predicted D-amino acid dehydrogenase	ABQ62519.1			**Virulent**	Cellular Process
1.4.-.-	Predicted D-amino acid dehydrogenase	ABQ62405.1			Non-virulent	Metabolism
1.5.1.20	Methylenetetrahydrofolate reductase	ABQ60279.1	Tetrahydrofolate metabolism	Nucleotide and cofactor metabolism	Non-virulent	Cellular Process / Metabolism
1.5.1.-	***Flavin reductase domain containing protein***	ABQ60228.1	Flavin metabolism	Nucleotide and cofactor metabolism	**Virulent**	Cellular Process / Metabolism
1.5.3.1	Sarcosine oxidase beta subunit	ABQ60177.1	Creatine degradation	Fermentation and other catabolism	**Virulent**	Cellular Process / **Virulence Factor**
		ABQ61310.1				
	Sarcosine oxidase alpha subunit	ABQ61036.1				
1.5.3.1	Predicted monomeric Sarcosine oxidase	ABQ62932.1	Creatine degradation	Fermentation and other catabolism	Non-virulent	Cellular Process / Metabolism
1.5.5.1	Electron transferring flavoprotein-ubiquinone oxidoreductase (ETF-QO)	ABQ61337.1	Oxidative phosphorylation	Central and energy metabolism	Non-virulent	Metabolism
1.6.5.2	WrpA type FMN-dependent NADH:quinone oxidoreductase	ABQ60884.1	Potential role in protection from stress damage	Defence from Host	Non-virulent	Metabolism
1.6.99.1	***NADPH dehydrogenase (Old yellow enzyme)***	ABQ62422.1	Unclear function	Unclear function	Non-virulent	Cellular Process
1.6.-.-	***NADH dehydrogenase***	ABQ62704.1	Unclear function	Unclear function	Non-virulent	Metabolism
1.7.-.-	***Predicted NAD(P)H nitroreductase***	ABQ60834.1	Oxidation-reduction of diverse nitrogen containing compounds	Nitrogen metabolism	Non-virulent	Metabolism
1.8.1.2	Assimilatory sulphite reductase (NADPH) alpha component cluster	WP_006015252.1	Sulphate reduction	Amino acid metabolism	**Virulent**	Cellular Process / Metabolism
		WP_006015255.1				
		WP_006015257.1				
		WP_006015259.1				
1.8.1.4	Dihydrolipoyl dehydrogenase (lpdA-1)	ABQ62466.1	Oxidative decarboxylation of pyruvate. Citric acid cycle. Glycine metabolism. Acetyl-CoA biosynthesis	Central and energy metabolism. Amino acid metabolism	Non-virulent	Cellular Process
1.8.1.4	Dihydrolipoyl dehydrogenase (IpdA-2)	ABQ60398.1			Non-virulent	Metabolism
1.8.1.4	Dihydrolipoyl dehydrogenase (IpdA-3)	ABQ61458.1			Non-virulent	Metabolism
1.8.1.7	Glutathione-disulphide reductase	ABQ61016.1	Thiol redox pathway. Glutathione metabolism	Control the redox state of the cell	Non-virulent	Cellular Process
1.8.1.9	Thioredoxin-disulphide reductase	ABQ60123.1	Thiol thioredoxin related pathway. Reduction of cytoplasmic enzymes	Control the redox state of the cell	Non-virulent	Metabolism
1.8.1.9	Predicted thioredoxin-disulphide reductase	ABQ61134.1		Control the redox state of the cell	Non-virulent	Metabolism
1.8.5.B1	Peptide-methionine (S)-S-oxide reductase (quinone) (Msr). MsrP catalytic subunit. MsrQ heme-binding subunit	ABQ62365.1	Methionine reparation of periplasmic proteins	Protection from stress damage. Defence from host	**Virulent**	Cellular Process / Metabolism
		ABQ62343.1				
1.13.11.32	Nitronate monooxygenase (formerly 2-nitropropane dioxygenase NPD)	ABQ62537.1	Alkylnitronates degradation	Nitrogen metabolism	Non-virulent	Metabolism
1.13.11.79	Predicted aerobic 5,6-dimethylbenzimidazole synthase (BluB)	ABQ62404.1	Vitamin B12 metabolism	Nucleotide and cofactor metabolism	**Virulent**	Metabolism
1.14.13.1	Predicted Salicylate hydroxylase	ABQ60137.1	Phenol degradation	Xenobiotic metabolism and secondary metabolism	Non-virulent	Metabolism
1.14.13.1	Predicted Salicylate hydroxylase	ABQ60978.1	Phenol degradation	Xenobiotic metabolism and secondary metabolism	**Virulent**	**Virulence Factor**
1.14.13.-	Predicted UbiH/COQ6 monooxygenase family	ABQ60166.1	Ubiquinone biosynthesis	Central and energy metabolism	Non-virulent	**Virulence Factor**
1.14.13. -	UbiH/UbiF family hydroxylase	ABQ62553.1	Ubiquinone biosynthesis	Central and energy metabolism	Non-virulent	**Virulence Factor**
1.14.13.2	4-hydroxybenzoate 3-monooxygenase	ABQ62030.1	4-hydroxymandelate degradation	Fermentation and other catabolism	Non-virulent	Metabolism
1.14.14.3	Bacterial luciferase	ABQ60348.1	Bioluminescence	Bacterial luminescence	Non-virulent	Cellular Process
1.16.1.4	Cob(II)alamin reductase	ABQ60249.1	Vitamin B12 metabolism	Nucleotide and cofactor metabolism	**Virulent**	Metabolism
1.17.1.4	Xanthine dehydrogenase, small subunit	ABQ61298.1	Purine metabolism	Nucleotide and cofactor metabolism	Non-virulent	Cellular Process / **Potential Virulence factor**
1.18.1.3-5	Predicted Ferredoxin/rubredoxin/ putidaredoxin NAD^+^ Reductase	ABQ62051.1	Protection from ROS stress damage	Defence from host	Non-virulent	**Virulence Factor**
1.18.1.2	Ferredoxin-NADP^+^ reductase	ABQ61707.1	Protection from ROS stress damage	Defence from host	Non-virulent	Metabolism
1.-.-.-	***Predicted nitroreductase family protein***	ABQ62091.1	Reduction of nitrogen containing compounds.	Nitrogen metabolism	Non-virulent	Metabolism
2.1.1.74	Methylenetetrahydrofolate-tRNA-(uracil54-C5-)-methyltransferase NAD(P)H oxidase	ABQ61275.1	Post-translationally modification of tRNA	Modification of cytoplasmic tRNAs	Non-virulent	**Virulence Factor**
2.1.1.229	tRNA (carboxymethyluridine34-5-O)-methyltransferase	ABQ60378.1	Post-translationally modification of tRNA	Modification of cytoplasmic tRNAs	Non-virulent	**Virulence Factor**
2.2.1.6	Acetolactate synthase 3 catalytic subunit	ABQ60081.1	Acetoin degradation, valine and isoleucine metabolism	Fermentation and other catabolism. Amino acid metabolism	Non-virulent	Metabolism
2.5.1.9	Riboflavin synthase alpha subunit	ABQ60518.1	Flavin biosynthesis	Nucleotide and cofactor metabolism	Non-virulent	Cellular Process / Metabolism
2.7.7.2	Bifunctional riboflavin kinase/FAD synthase	ABQ62831.1	Flavin biosynthesis	Nucleotide and cofactor metabolism	Non-virulent	Metabolism
2.7.1.26						
2.7.1.180	FAD:protein FMN transferase	ABQ62066.1	Flavin transfer	Maturation of other enzymes	Non-virulent	Metabolism
2.7.13.3	Blue-light-activated histidine kinase	ABQ62113.1	Light activated phosphorylation	Signal transduction. **Virulence factor**	Non-virulent	Cellular Process / **Potential Virulence Factor**
4.1.1.36	Coenzyme A biosynthesis bifunctional protein	ABQ62036.1	Coenzyme A metabolism	Nucleotide and cofactor metabolism	Non-virulent	Metabolism
6.3.2.5						
4.2.3.5	Chorismate synthase	ABQ60200.1	Chorismate metabolism	Amino acid metabolism	Non-virulent	Cellular Process
7.1.1.2	NADH-quinone oxidoreductase subunit F (H^+^ translocating)	ABQ60521.1	Oxidative phosphorylation	Central and energy metabolism	Non-virulent	Cellular Process
	Electron transferring flavoprotein alpha subunit (ETFa)	ABQ61011.1	Oxidative phosphorylation	Central and energy metabolism	Non-virulent	Cellular Process / Metabolism
	Electron transferring flavoprotein beta subunit (ETFb)	ABQ60428.1				
	Protein NrdI	ABQ62891.1	Electron exchange, nucleotide transport and metabolism	Nucleotide and cofactor metabolism	**Virulent**	Cellular Process

a Shown in bold and italics are those candidates for which a clear function cannot be depicted.

b VirulentPred was used to predict potential virulent proteins based on amino-acid compositions, similarity, position specific scoring matrix, dipeptide composition, higher order dipeptide composition, and the bi-layer cascade Support Vector Machine module. When one of these approaches produced a match, the protein was label as virulent. Positive virulence is highlighted in bold.

c The pattern based score of VICMpred was used to classify potential functions for proteins among cellular process, metabolism, signaling, or virulence factor. Positive virulence highlighted in bold.

Two flavoproteins are predicted not holding catalytic activity by themselves: ETFa and NrdI. The ETFa together with the beta subunit (ETFb) specifically transfer electrons from different dehydrogenases to the respiratory electron transfer chain (ETC) via the electron transferring flavoprotein-ubiquinone oxidoreductase (ETF-QO). Noticeably, *etfA* and *etfB* encoding genes in B. ovis sit next to a gene encoding for an NADP^+^ dependent butyryl-CoA dehydrogenase (Table SP4). This suggests that these three proteins might form a BCD bifurcating complex responsible for crotonyl-CoA reduction during butanol production. Homologues of Clostridium acetobutylicum have the same organization (40), and ETF proteins are also pointed to as potential targets for the treatment of some bacterial infections (41). NrdI is a flavodoxin-like electron-transport protein with potential analogous functions to ferredoxins. It belongs to the core proteome of Brucella but is hardly populated in the evaluated alphaproteobacteria (Table SP6).

### B. ovis flavo-oxidoreductases participate in a large variety of metabolic pathways.

Most B. ovis flavoenzymes are oxidoreductases and particularly belong to subclasses that use alcohols (EC 1.1.), CH-CH (EC 1.3.), CH-NH_2_ (EC 1.4.), CH-NH (EC 1.5.), or sulfur (EC 1.8.) groups as hydrogen or electron donors, as well as paired donors with incorporation or reduction of molecular oxygen (EC 1.14.). Some use as donors NAD(P)H (EC 1.6.), metals (EC 1.16), or nitrogenous compounds (various EC 1. subclasses) (Tables 1–3, Fig. 7A).

The subclass alcohol flavo-oxidoreductase (Fig. 4A) includes three enzymes of the GMC family (EC 1.1.99.1). This large and diverse protein family shares structural fold and reaction mechanisms, with substrates ranging from sugars and alcohols to cholesterol and choline, which are usually related to the metabolism of methyl groups through betaine (Tables 1–3). They are present in yeasts, bacteria, insects and filamentous fungi, being the latest use for biomass utilization, biosensors, or food industry (42). This subclass has also two alcohol oxidases that share 55% identity and phylogenetically cluster together (bootstrap >98) (Fig. 4A). One is a canonical glycerol-3-phosphate dehydrogenase (GlpD) (EC 1.1.5.3, ABQ60174.1), and the other is envisaged erythritol oxidation activity (EC 1.1.1.402, ABQ62056.1) from gene context (BOV_RS14450, *eryB*) and phylogenetic analysis (Tables 1–3, Fig. 4A). They likely supplement electrons for aerobic oxidative phosphorylation (OXPHOS) at the central junction of glycolysis, respiration, and phospholipid biosynthesis, being essential for aerobic growth on glycerol-like molecules (43). Noticeably, erythritol has a growth-promoting effect on intracellular Brucella pathogens (44, 45). GlpD is in the Brucella core proteome, but EryB lacks in some Brucella (Table SP6, Fig. 4A). These observations highlight the importance of the shuttle of electrons in the Brucella metabolism. Other enzymes in this subclass are oxygen oxidoreductases. One is the GlcD subunit of glycolate dehydrogenase that catalyzes oxidation of glycolate and d-lactate, respectively, to glyoxylate and pyruvate (EC 1.1.99.2), a key function in microbial redox oxidative metabolisms. Glycolate dehydrogenase is usually built by several subunits, including a GlcE one. In B. ovis the gene upstream of *glcD* is a *glcE* pseudogene (Table SP5). Nonetheless, the oxygen oxidoreductases ABQ60928.1 and ABQ61939.1 have similar features to GlcE and not precise function, so they might replace the nonfunctional GlcE protein (Table 2 and Table SP6). The protein ABQ62001.1 (EC 1.1.3.8) is also proposed as oxygen oxidoreductase. It has a nonclassified long C-terminal (Table 2) and its closer available structural homologue is decaprenyl-phosphoryl-β-d-ribofuranose-2-oxidoreductase from Mycobacterium smegmatis, an essential enzyme in cell wall biosynthesis (46). This subclass also includes the hydroxyglutarate dehydrogenase (EC 1.1.99.2) and the FMN-dependent L-lactate dehydrogenase (EC 1.1.2.3), both in the core proteome of Brucella (Table SP6). Altogether, alcohol flavo-oxidoreductases contribute to the energetic intake metabolism for B. ovis survival.

The subclass EC 1.3 includes seven acyl-CoA dehydrogenases (ACAD) (EC 1.3.8.-) widely represented in Brucella but not within alphaproteobacteria (Fig. 5A, Table SP6). The most divergent ACAD (ABQ62576.1) has an AidB domain instead of an Acyl-CoA_dh_N one, and is 100% identical to a protein from B. melitensis whose 3D structure is available (PDB 5EZ3, Table SP3, Fig. SP2). It has all features of B. abortus and E. coli homologues involved in the destruction of alkylating agents, suggesting it will provide resistance during alkylation stress as well as in cell division (47). The ACAD annotated as isovaleryl-CoA dehydrogenase (ABQ60382.1) in UniProtKB might participate in the catabolism of branched chain amino acids. The other five ACADs could participate in fatty acid β-oxidation (Tables 1–3), suggesting the use for B. ovis of lipids, probably recruited from host cells, as carbon sources (7). This subclass also includes the SdhA subunit of succinate dehydrogenase (Sdh) (EC 1.3.5.1) and the UDP-N-acetylmuramate dehydrogenase (MurB) (EC 1.3.1.98), two proteins that are in the core proteome of Brucella and conserved in all evaluated alphaproteobacteria (Fig. 5A, Table SP6). The Sdh complex is built by different subunits (Table SP4), localizes in the membrane of many bacteria, and catalyzes the oxidation of succinate to fumarate. It uses membrane quinone as electron acceptor and is the only enzyme linking the tricarboxylic acid cycle and the ETC (48). MurB catalyzes the NADPH dependent reduction of UDP-*N*-acetylglucosamine-enolpyruvate to UDP-N-acetylmuramic acid (EC 1.3.1.98) and participates in the biosynthesis of peptidoglycan building blocks (49). Subclass EC 1.3 also includes two subunits of the dihydropyrimidine dehydrogenase (EC 1.3.1.1) involved in the β-alanine metabolism, the biosynthesis of pantothenate and CoA, and the pyrimidine nucleotide metabolism; two enzymes involved in the modification of cytoplasmic tRNAs (EC 1.3.1.88, 1.3.1.91); one PyrD dihydroorotate dehydrogenase (EC 1.3.5.2) involved in nucleotide metabolism; and finally one alkene reductase (EC 1.3.1.-/1.7.1.B1) and one KsdD-like steroid dehydrogenase (EC 1.3.99.-) that might, respectively, contribute to toxic compounds degradation and oxidation/dehydrogenation of ketosteroids.

Flavoenzymes of subclass EC 1.4 are highly conserved within Brucella, but some are hardly present in alphaproteobacteria (Fig. 4B, Table SP6). They particularly include a pyridoxamine 5′-phosphate oxidase family protein of unclear function present in the Brucella core proteome, but only in five of the symbiotic alphaproteobacteria evaluated (Agrobacterium tumefaciens, Mesorhizobium loti, Rhizobium etli, Rhizobium leguminosarum, and Sinorhizobium meliloti). This suggests that it might be involved in nitrogen metabolism (Tables SP3 and SP6). Its crystal structure, solved for B. melitensis, predicts a dimer that might bind either FMN, FAD, or F420 (8-hidroxi-5-deazaflavina) (Fig. SP3). Its genomic context, next to a PhzF family phenazine biosynthesis protein gene (downstream), is pretty similar in homologues. Moreover, in three Brucella (B. melitensis, B. canis, and B. microti) a gene for a Nudix hydrolase follows the *phzF* gene, and in the alphaproteobacteria Ochrobactrum anthropi these genes are grouped together. Therefore, this protein might be somehow related to the hydrolysis of nucleoside diphosphates linked to other biomolecules. This subclass also includes glycine oxidase (ThiO, EC 1.4.3.19) involved in glycine oxidation to glyoxylate and in the thiamine metabolism; a potential D-amino acid oxidase (EC 1.4.99.-) with low similarity to other characterized enzymes of this type; pyridoxamine 5′-phosphate oxidase (EC 1.4.3.5) involved in the biosynthesis of pyridoxal 5′-phosphate (50); the two flavoprotein subunits of a glutamate synthase that through three distinct active centers (EC 1.4.1.13) uses of l-glutamine as carbon and nitrogen source during cell growth, particularly within the host as shown by B. abortus (51); and two proteins folding in HI0933_like domains that are potential NAD(P)/FAD-dependent dehydrogenases of the aminoacetone oxidase family (EC 1.4.3.-). We cannot unambiguously provide a clear function for these two last proteins, but they might have a particular role in Brucella because they are in its core proteome but poorly distributed in alphaproteobacteria (Table SP6).

The subclass EC 1.5 (Fig. 6B) includes the methylenetetrahydrofolate reductase (EC 1.5.1.20), a central enzyme in the carbon fixation and tetrahydrofolate metabolisms; the iron-sulfur flavoprotein ETF-QO (EC 1.5.5.1) that accepts electrons from ETF proteins and contributes to the respiratory chain and OXPHOS pathways (52); the flavin reductase domain containing protein (EC. 1.5.1.-) that is the most divergent member and might have flavin reductase activity; and two sarcosine oxidase proteins (EC 1.5.3.1). One sarcosine oxidase (ABQ62932.1) relates to the monomeric soluble protein, whereas the other is composed by two subunits that bind flavins, alpha (ABQ61036.1) and beta (ABQ60177.1+ABQ61310.1), and is the membrane bound form. Sarcosine oxidases would catalyze demethylation of sarcosine as a way for B. ovis to grow with sarcosine, ensuring creatinine degradation and glycine, serine, and threonine metabolism. All these flavoproteins of subclass EC 1.5 are in the core proteome of Brucella, but are not conserved in alphaproteobacteria (Table SP6).

The subclass EC 1.6 is represented by three flavoenzymes. The WrpA-type FMN-dependent NADH:quinone oxidoreductase (ABQ60884.1, EC 1.6.5.2) is present in all Brucella (Table 3 and Table SP6). The B. abortus homologue structure (98.5% identity) relates it to NADH:quinone oxidoreductases, but *in vitro* its ability to bind redox cofactors and its oxidoreductase activity have not been proven. However, it modulates B. abortus interaction with the mammalian host and is suggested as a new functional class of WrpA/flavodoxin family proteins likely involved in cell survival under acute oxidative stress (53). This subclass also includes the FMN-dependent NADPH dehydrogenase of the old yellow enzyme family (EC 1.6.99.1). It is not present in all Brucella and alphaproteobacteria, and despite being highly studied in different species, its acceptor and physiological function remain unclear. Finally, this subclass includes a FAD-dependent NADH dehydrogenase with unknown function (EC 1.6.-.-).

Up to eight flavoenzymes fall in the subclass EC 1.8. The sequence identity of the B. ovis FAD dependent glutathione-disulphide reductase (EC 1.8.1.7) with the S. meliloti homologue indicates that it must contribute to maintain high levels of reduced glutathione to control redox homeostasis. This agrees with a recent report where disruptions in the gene encoding for it in B. ovis produce a significant disadvantage in bacterial growth (54, 55). The three predicted dihydrolipoyl dehydrogenases (ldpA-1, ldpA-2, ldpA-3, EC 1.8.1.4) are highly conserved in Brucella and alphaproteobacteria, with the exception of ldpA-1 poorly represented in alphaproteobacteria. Homologues in B. suis and B. abortus form part of complexes such as the alpha-ketoacid dehydrogenase, pyruvate dehydrogenase, and glycine cleavage multienzyme, implicated in the biosynthesis of Acetyl-CoA and secondary metabolites, oxidative decarboxylation of pyruvate, and glycine metabolism, which contribute to the bacteria pathogenicity (Table SP7) (56–58). The MsrQ subunit of peptide-methionine (S)-S-oxide reductase (quinone) (EC 1.8.5.B1) uses FMN and haem, and is complemented with molybdopterin and quinone at the MsrP subunit. Msr complex is essential for the maintenance of envelope integrity under bleach stress and protects proteins from oxidative-stress damage during host defense mechanisms (59). MrsQ is common to all Brucella, but not in alphaproteobacteria. This subclass also has the two flavoprotein subunits of the assimilatory sulfite reductase (NADPH): the alpha component cluster that, together with a beta subunit, catalyzes the six-electron reduction of sulfite to sulfide (EC 1.8.1.2). This protein is usually involved in sulfate and sulfur assimilation, and in microbial metabolism in diverse environments. As mentioned above, it is singular in B. ovis, since in other Brucella a single protein contains the four components (Fig. 2B). In addition, this subclass has two FAD-dependent thioredoxin-disulphide reductase like proteins (EC 1.8.1.9) with pyridine nucleotide-disulphide oxidoreductase activity potentially involved in the oxidation-reduction cycle of thioredoxin.

Most flavoenzymes of subclass EC 1.14 use NAD(P)H as donor and incorporate oxygen into the second substrate (Fig. 7B). Two of them are related to salicylate hydroxylase activity (EC 1.14.13.1) contributing to the degradation of aromatic compounds, and two are members of the UbiH/COQ6 monooxygenase and UbiH/UbiF hydroxylase families (EC 1.14.13.-) (Table SP6). These latter two enzymes are involved in the ubiquinone biosynthesis pathway (ARBA annotation: ARBA00004749, “ubiquinone biosynthesis”) and share moderate sequence similarity with well-characterized flavoprotein monooxygenases, but close homologues have not been characterized yet (60–62). This subclass also has the 4-hydroxybenzoate 3-monooxygenase (EC 1.14.13.2) that participates in the benzoate degradation and favors microbial metabolism in diverse environments (63, 64). Another member is the bacterial luciferase (EC 1.14.14.3) that incorporates oxygen into reduced FMN to form a peroxyflavin-adduct that upon interaction with aliphatic long-chain aldehydes produces highly fluorescent species. This luciferase is particularly common in symbiotic nitrogen-fixing alphaproteobacteria (A. tumefaciens, R. leguminosarum, S. meliloti, except R. pomeroyi).

We have identified a single member of the subclass EC 1.16. This protein is assigned in UniProtKB as FMN-dependent 4-hydroxyphenylacetate 3-monooxygenase (EC 1.14.14.9, ABQ60249.1). However, it clearly diverges from subclass EC 1.14 (Fig. 6B) and is identical to the Cob(II)alamin reductase (EC 1.16.1.4) of B. melitensis with structure and activity experimentally proven (65). Therefore, ABQ60249.1 must participate in the cobalamin (vitamin B_12_) biosynthetic pathway. The single member of subclass EC 1.17 is the small subunit of xanthine dehydrogenase (*xdhA*) (EC 1.17.1.4) that is present in most Brucella evaluated. This enzyme participates in the metabolism of purines and is made by several subunits.

The subclass EC 1.18. has two flavoenzymes that exchange electrons between pyridine nucleotides and iron-sulfur proteins. One is the bacterial type ferredoxin-NADP^+^ reductase (EC 1.18.1.2), widely distributed in Brucella but not in alphaproteobacteria, and for which 3D structure and mechanism of action as NADPH oxidoreductase are reported (39). It probably delivers electrons from NADPH to the redox-based metabolism. But considering that a superoxide dismutase is expressed downstream, it might also oxidize NADPH to activate regulons that protect against oxidative damage (66) (Table SP4). The other member is the ferredoxin/rubredoxin/putidaredoxin NAD^+^ reductase (EC 1.18.1.3-5), with also a potential role in cellular oxidative stress response or lipid metabolism.

Finally, four FMN dependent flavoreductases are predicted to act on nitrogenous compounds. The nitroreductase family protein (EC 1.-.-.-) has unknown precise function. The nitronate monooxygenase (EC 1.13.11.32) is predicted to use molecular oxygen to oxidize alkyl nitronates, and to produce enzyme-bound nitronate radicals and peroxynitroethane species. The aerobic dimethylbenzimidazole synthase (BluB, EC 1.13.11.79) putatively catalyzes the oxygen-dependent oxidative fragmentation of the reduced isoalloxazine of FMN to yield 5,6-dimethylbenzimidazole in the biosynthesis of cobalamin (67, 68). Finally, it is the NAD(P)H nitroreductase like protein (EC 1.7.-.-) that might oxidize diverse nitrogen-containing compounds (69).

### Flavoenzymes of the transferase class show varied activities in B. ovis.

Flavotransferases (EC 2.) in B. ovis use different structural scaffolds and catalyze quite different reactions. Their distribution varies among Brucella and alphaproteobacteria species (Table SP6). Two of them fold in GIDA domains and act in the posttranslational modification of tRNAs (2.1) (Table 3). Three others participate in consecutive steps of the biosynthesis of flavin cofactors: the riboflavin synthase (EC 2.5.1.9); the bifunctional FADS with two independent transferase sites (EC 2.7.7.2, EC 2.7.1.26) that has recently been characterized showing species-specific traits in both of its activities (70); and a FAD:protein FMN transferase (EC 2.7.1.180) potentially involved in the transfer of the FMN moiety from FAD to a target flavoprotein. This class includes also the acetolactate synthase 3 (EC 2.2.) that transfers acetaldehyde from one pyruvate to either another pyruvate or 2-oxobutanoate (EC 2.2.1.6) in the respective valine and isoleucine biosynthetic pathways (71); and the blue-light-activated histidine kinase (EC 2.7.13.3). This latter enzyme undergoes photochemistry through its FMN chromophore by formation of a cysteinyl-flavin adduct that allosterically controls the enzymatic activity at its kinase protein domain (Fig. 3C) (72).

### Lyases and translocases have a minor representation in the flavoproteome of B. ovis.

Flavoproteins acting as lyases use the less common folding. One is chorismate synthase (EC. 4.2.3.5) that catalyzes the formation of chorismate, a starting substrate in the biosynthesis of aromatic amino acids (73). The other is a bifunctional enzyme that catalyzes two sequential steps in coenzyme A biosynthesis: the CTP dependent conjugation of cysteine and 4′-phosphopantothenate to form 4-phosphopantothenoylcysteine (EC 6.3.2.5), followed by the FMN-dependent decarboxylation of this product to 4′-phosphopantotheine (EC 4.1.1.36) (74).

Only one flavoprotein of the core proteome of Brucella (Table SP6) is predicted as a translocase: the nuoF subunit of the complex I-like NADH quinone oxidoreductase that catalyzes the translocation of protons across membrane linked to a FMN dependent NADH dehydrogenase activity (EC 7.1.1.2) and that contributes therefore to aerobic respiration and OXPHOS (75).

### The B. ovis flavoproteome in virulence and infectivity.

In general, Brucella spp. do not show aggressive virulence mechanisms such as exotoxins, anti-phagocytic capsules, plasmids, fimbria, flagella, or antigenic variation. Nonetheless, they are highly pathogenic for their preferred or accidental hosts and their silent capacity to adapt to the intracellular environment. They are considered an evolutive virulence factor by themselves (76). In particular, B. ovis shows some peculiarities: (i) it does not produce H_2_S, does not hydrolyze urea, and does not reduce nitrate, contrary to most Brucella *spp*., (ii) its lipopolysaccharide protective envelope is naturally rough, and (iii) it is the unique Brucella able to oxidize ribitol with the exception of B. neotomae (18, 77). The flavoproteome partially contributes to these evolutionary abilities. An example is EryB, particularly present in species that cause abortions, as B. ovis, B. melitensis and B. abortus (78–81).

Thus, the B. ovis flavoproteome can be a source of virulence, infectivity, and survival factors, whose distribution varies among the close analyzed species (Tables 3 and Table SP7). Two potential virulent candidates belong to the core proteome of alphaproteobacteria: MurB and SdhA. Nonetheless, despite predictors indicating MurB as a potential virulent/infectivity factor, there is no experimental evidence beyond its essential housekeeping role to maintain the peptidoglycan cell wall (82). On the contrary, SdhA is a requirement for pathogenicity in E. coli (48), to stabilize the vacuole integrity during replication in the intracellular pathogen (like B. ovis) Legionella pneumophila (83), and its reduction is detected early during infection in B. abortus (84). Moreover, the *SdhB* gene is virulent in L. pneumophila (85), and the SdhB subunit plays a role in filamentation and virulence in Candida albicans (86). The integrity of Sdh subunits is also related to antibiotic resistance in Salmonella enterica and Xanthomonas oryzae *pv*. *Oryzae* (87, 88). Up to 17 of the flavoproteins predicted as potentially virulent factors in B. ovis belong to the core proteome of Brucella (or lack in a single species), and are already noticed as involved in the infection process of different pathogens (Table 3; Tables SP6 and SP7). Among them we can highlight the following: (i) Msr that maintains bacterial membrane integrity and contributes to adhesion with eukaryotic cells (59, 89); (ii) two D-amino acid dehydrogenases that could play a pleiotropic role in the production of important virulence factors and support pathobiological exclusive functions for different isoforms within an organism (90, 91); (iii) one isovaleryl-CoA dehydrogenase involved in vegetative growth, conidiation, and virulence of plant fungal pathogens (92); (iv) one glutamate synthase involved in chronic persistence of B. abortus infection in mice (51); (v) one cob(II)alamin reductase conserved in most alphaproteobacteria and whose deletion in B. abortus affects the pathogen survival (93); (vi) two tRNA methyltransferases with role in virulence, stress response, growth, and antibiotic susceptibility pathways (94); and (vii) NrdI, essential for the assembly of several subunits of class Ib ribonucleotide reductases expressed under oxidative stress and iron-limited growth conditions (95).

Other predicted virulent factors common in nearly all Brucella are the membrane bound sarcosine oxidase, the blue-light-activated histidine kinase, and the pyridoxamine 5′-phosphate oxidase family protein. The blue-light-activated histidine kinase increases its own autophosphorylation to modulate the microorganism virulence in B. abortus (72, 96, 97). The pyridoxamine 5′-phosphate oxidase family protein is very rare in other bacteria, but its conservation within Brucella suggests a particular still unclear function. In addition, B. ovis contains an important number of flavoproteins (some highly conserved in Brucella, but not all) whose homologues are required for the survival of different pathogens upon infection by acting in key metabolic pathways and suppressing host defenses (Table SP7).

It is also interesting to note that the B. ovis genome contains a large amount of transposable recombinogenic elements and pseudogenes (up to 119 in CI and 125 in CII) that can contribute to its variability and adaptive and evolutionary capacities (98). Many of them sit next to or in flavoenzyme encoding genes (Tables SP4 and SP5). For example, one IS5 transposase is located between the gene encoding the pyridoxamine 5′-phosphate oxidase family protein and the gene encoding for the PhzF family phenazine biosynthesis protein. Other IS5 transposase interrupts the two *SoxB* genes (Fig. 2A, Table SP4), similarly to that reported in the Pseudomonas aeruginosa PAO1 and related to a reduction of pathogenicity (99). Regarding pseudogenes, the B. ovis xanthine dehydrogenase operon contains a pseudogene instead of a regular *xdhB* encoding region for the corresponding protein subunit (Table SP4). This XdhB subunit is not expected to bind flavin, but its lack will make xanthine dehydrogenase not functional (18). The B. ovis BOV_RS11620 gene encoding for the NosR transcriptional regulator of the expression of the nitrous oxide reductase NosZ also has a deletion in its FMN_bind domain (PF04205). The *nosX* gene, necessary for NosR covalent flavinylation, is in addition a pseudogene (BOV_RS11650) (100). These features introduce defects in the nitrogen metabolism of B. ovis, and contrary to other Brucella make it particularly unable for full denitrification and nitrous oxide respiration (101). Thus, degradation of the B. ovis flavoproteome surely contributes to narrow its host range and to make it nonzoonotic (18).

### The B. ovis flavoproteome as a source of antimicrobial targets and biocatalyst.

Up to 35 B. ovis flavoproteins, most of them belonging to the Brucella core proteome, lack homologues in *O. aries* and other mammals (Table 4). This list could be potential targets for the search for antimicrobials. Some of them are already being explored as targets of inhibitors in other bacteria, as for example, UDP-N-acetylmuramate dehydrogenase (102, 103), riboflavin synthase (104), bifunctional riboflavin kinase/FAD synthase (25, 26), or chorismate synthase (105). In agreement, a comparative metabolomics study in B. melitensis also pointed to synthase as an attractive target (106). Others with certain homology to the here-identified thioredoxin-disulphide reductase (107) or FAD:protein FMN transferase are also considered antimicrobial targets (108). Noticeably, Table 4 includes an important number of the B. ovis flavoproteins for which the exact physiological function is difficult to envisage. Among them are predicted alkene reductase and KsdD-like steroid dehydrogenase, the pyridoxamine 5′-phosphate oxidase family protein, two potential aminoacetone oxidase family FAD-binding dehydrogenases, two potential D-amino acid dehydrogenases, the predicted nitroreductase family protein, and the NADPH dehydrogenase from the old yellow enzyme family. Of interest, the latter enzyme has been reported to participate in the oxidative stress response and detoxification in B. subtilis (109), which points to it as an interesting target to control pathogen survival. Moreover, seven of these B. ovis flavoproteins (Table 4) are underrepresented in alphaproteobacteria, suggesting that they might be explored also as potential selective antimicrobial targets. Among them are the pyridoxamine 5′-phosphate oxidase family protein, the NADPH dehydrogenase old yellow enzyme, and the predicted nitroreductase family protein, all of them of still unclear function. This group is completed with the predicted salicylate hydroxylase, the FAD:protein FMN transferase, the blue-light-activated histidine kinase, and the protein NrdI. Considering their above-mentioned envisaged roles for virulence upon infection in different bacteria (Table SP7), these four proteins might be also of particular relevance as drug targets (95, 96).

**TABLE 4:** Brucella ovis ATCC 25840 flavoproteins lacking homologues in *O. aries* and Mammalia^*a*^

Flavoprotein	Protein code	Flavoprotein	Protein code
Potential L-gulonolactone oxidase FAD-binding oxygen oxidoreductase	ABQ62001.1	Peptide-methionine (S)-S-oxide reductase (quinone). MsrP catalytic subunit.	ABQ62365.1
UDP-N-acetylmuramate dehydrogenase	ABQ61769.1	Nitronate monooxygenase (formerly 2-nitropropane dioxygenase NPD)	ABQ62537.1
Predicted alkene reductase: N-ethylmaleimide reductase, glycerol trinitrate reductase or xenobiotic reductase B	ABQ62490.1	Predicted aerobic 5,6-dimethylbenzimidazole synthase (BluB)	ABQ62404.1
Predicted KsdD-like steroid dehydrogenase	ABQ62061.1	Predicted Salicylate hydroxylase	ABQ60137.1
Pyridoxamine 5′-phosphate oxidase family protein^*b*^^,^^*c*^	ABQ61684.1	Predicted Salicylate hydroxylase^*b*^	ABQ60978.1
Glycine oxidase ThiO	ABQ60316.1	4-hydroxybenzoate 3-monooxygenase	ABQ62030.1
Potential Aminoacetone oxidase family FAD-binding enzyme/ NAD(P)/FAD-dependent dehydrogenase	ABQ60616.1	Bacterial luciferase	ABQ60348.1
Potential Aminoacetone oxidase family FAD-binding enzyme/ NAD(P)/FAD-dependent dehydrogenase	ABQ60524.1	Cob(II)alamin reductase	ABQ60249.1
Predicted D-amino acid dehydrogenase small subunit	ABQ61937.1	Ferredoxin-NADP^+^ reductase	ABQ61707.1
Predicted D-amino acid dehydrogenase	ABQ62519.1	Predicted nitroreductase family protein^*b*^	ABQ62091.1
Flavin reductase domain containing protein	ABQ60228.1	Methylenetetrahydrofolate-tRNA-(uracil54-C5-)-methyltransferase NAD(P)H oxidase	ABQ61275.1
Predicted monomeric Sarcosine oxidase	ABQ62932.1	Acetolactate synthase 3 catalytic subunit	ABQ60081.1
WrpA-type FMN-dependent NADH:quinone oxidoreductase	ABQ60884.1	Riboflavin synthase alpha subunit	ABQ605180.1
NADPH dehydrogenase (Old yellow enzyme)^*b*^	ABQ62422.1	Bifunctional riboflavin kinase/FAD synthase	ABQ62831.1
Predicted NAD(P)H nitroreductase^*c*^	ABQ60834.1	FAD:protein FMN transferase^*b*^	ABQ62066.1
Assimilatory sulphite reductase (NADPH) alpha component cluster^*c*^	WP_006015252.1	Blue-light-activated histidine kinase^*b*^	ABQ62113.1
	WP_006015255.1	Chorismate synthase	ABQ60200.1
	WP_006015257.1	Protein NrdI^*a*^	ABQ62891.1
Predicted thioredoxin-disulphide reductase	ABQ61134.1		

a Threshold set in at least 30% sequence identity over 50% of the query cover.

b Proteins underrepresented in alpha-proteobacteria.

c Proteins without homologues in any Eukarya.

In addition, Table 4 might also contain flavoenzymes with particular properties for their use in organic synthesis, biocatalysis, and/or bioremediation. Some of them might be predicted nitroreductase family protein or nitronate monooxygenase. If, as envisaged, they contribute to the catabolism of nitroalkanes, widely used in chemical industry and as fuels, their low homology to other family members might provide them with particular stability or mechanistic features that would enlarge their applicative perspectives (110). In any case, before used as either antimicrobial targets or biocatalyst, these flavoproteins should be exhaustively characterized at the structural and functional levels to confirm their relevance for bacteria survival, and investigated for their species-specific features and/or the applicability of the chemical process they catalyze.

## DISCUSSION

The predicted flavoproteins of B. ovis are envisaged to catalyze an important number of reactions in a large number of metabolic pathways, being particularly involved in the shuttle of electrons to the bacterial metabolism, the primary and energy metabolism, the metabolism of fats, carbohydrates, proteins, and nucleotides, the oxidative stress response, and the tRNAs methylation (Fig. 7B, Table 3), according to previous reports (9, 12, 15, 111–116). Moreover, the B. ovis flavoproteome also contains enzymes that are candidates to favor the microbial metabolism in diverse environments, the xenobiotic metabolism for detoxification of aromatic compounds, the bacterial virulence, or the activation of metabolites (pyruvate, folate, pyridoxal 5′-phosphate, vitamin B2, vitamin B12, etc.). Therefore, flavoproteins and flavoenzymes are implicated in the transformation of a vast variety of metabolic bioactive compounds or are directly involved in suppressing the stress induced by the host cells upon infection, which can make some of these proteins potential targets in the treatment of brucellosis. Noticeably, in B. ovis, 55% of 78 predicted flavoproteins belong to the core proteome of Brucella, whereas only 18% lack in 25% of the Brucella species here evaluated (Table SP6). This indicates a heavy dependence of the Brucella metabolism on flavoproteins. Moreover, many of these core flavoproteins are particular to Brucella, since very few are found in all alphaproteobacteria evaluated: namely, Sdh, dihydrolipoyl dehydrogenases 2 and 3, thioredoxin-disulphide reductase, and MurB. Nonetheless, the study of the B. ovis flavoproteome also shows some of its members are degraded, and probably unable to be functional, introducing variability in the capacities of this bacteria regarding other members of the genus.

### Concluding remarks.

In the last decades many efforts have been done in sequencing different genomes. Many proteins with undetermined or putative functions have been identified, but so far little has been done to elucidate or corroborate their biological activity. In this context, it is of relevance to predict and evaluate the functionality of candidates for flavoproteins in particular organisms. Here we provide the list of proteins making the flavoproteome in B. ovis, as well as data of their potential activities and prevalence in different Brucella and alphaproteobacteria species. Several predicted flavoproteins are highly divergent in this genus from revised proteins, and for them is difficult to envisage a clear function. This will probably relate to modified activities or divergent processes and mechanisms still not identified. Based on the compiled information here, we also identify some flavoproteins that might become potential antimicrobial and envisage that others might become new biocatalysts.

## MATERIALS AND METHODS

### Flavoprotein sequence searching.

Sequences for potential flavin-dependent proteins in Brucella ovis ATCC 25840 were retrieved from the National Center for Biotechnology Information (NCBI) and UniProtKB databases, and the genome and proteome ensembles of the bacteria. We searched for proteins binding RF, FMN, and FAD as ligands (117, 118). Sequences annotated in other species as consensus motifs for FMN- and FAD-binding were also used as queries to retrieve putative B. ovis flavoproteins using BLASTp online tools (7, 119, 120). The identified putative B. ovis flavoproteins were analyzed in the context of homologous flavin-dependent proteins reported in bacteria, archaea, eukaryotes, fungi, plants, and mammals.

B. ovis flavoprotein orthologues were retrieved in 19 Brucella species with complete genome sequenced and in 26 alphaproteobacteria representatives of this bacteria family with complete genome available (Table SP1).

### Flavoprotein classification.

The Pfam database was used to classify the retrieved flavoproteins in families and clans (121). Enzyme Commission numbers (EC numbers), protein names, or metabolic functions were assigned after examining each protein for their information in homologues from different organisms using different databases. These included the BRENDA database (122), the Enzyme Structures database (http://www.ebi.ac.uk/thornton-srv/databases/enzymes/), the KEGG PATHWAY database (https://www.genome.jp/kegg/pathway.html), the MetaCyc database collection (https://metacyc.org/), and the Pathogen-Host Interaction Data Integration Analysis System (Phidias), particularly the Brucella Bioinformatics Portal (BBP) containing 17 Brucella genomes (123, 124).

Potential virulent protein sequences were predicted using the pipelines of the servers VirulentPred (http://203.92.44.117/virulent/), a bi-layer cascade Support Vector Machine (SVM) methods developed for bacterial pathogens, (125) and VICMpred (https://bio.tools/vicmpred), specifically designed for Gram-negative bacterial proteins and also predicting general functional class (126).

### Structural modeling.

The BLASTp server was used to obtain identities and to search for the sequences of the most similar proteins with structures in the Protein Data Bank (PDB) (https://www.rcsb.org/), and for available 3D structures of flavin-dependent proteins of the Brucella genus. 3D structural homology models were built based on templates having at least 35% sequence identity using the Swiss-Model (127) and/or RaptorX (128) servers. Confidence scores calculated to indicate the quality of predicted 3D models were *P* value for the relative global quality, global distance test (GDT), and un-normalized GDT (uGDT) for the absolute global quality.

### Sequence alignment and phylogeny tree.

Sequence alignment and phylogenetic analysis were performed as described (129, 130). For phylogeny analysis the sequence profiles were globally aligned with Clustal Omega (https://www.ebi.ac.uk/Tools/msa/clustalo/) (131) and trimmed following the protocol of the TRIMAL software (132). A maximum likelihood phylogenetic tree using the Subtree Pruning and Regrafting (SPR) method was constructed with PHYML (https://ngphylogeny.fr) (133). The tree and cladogram were midpoint-rooted and plotted with FigTree (http://tree.bio.ed.ac.uk/software/figtree/). The approximate Likelihood-Ratio Test (aLRT) with a seed value of 123456 and bootstrap analyses with a value of 100 were performed. aLRT statistics: 0.022 proportion of invariant.
