# Supplementary material for: Mining the Flavoproteome of Brucella ovis, the Brucellosis Causing Agent in Ovis aries
Source: Microbiol Spectr. 2022 Mar 22;10(2):e02294-21. doi: 10.1128/spectrum.02294-21 (PMC9045290; doi:10.1128/spectrum.02294-21)
Supplement: SUPPLEMENTAL FILE 1 — Supplemental material. Download SPECTRUM02294-21_Supp_1_seq11.pdf, PDF file, 1.2 MB [file spectrum02294-21_supp_1_seq11.pdf]

SUPPLEMENTARY MATERIAL

Mining the flavoproteome of *Brucella ovis*, the brucellosis causing agent in *Ovis aries*

Martha Minjárez-Sáenz[1,2], Marta Martínez-Júlvez[1,2,4*], Inmaculada Yruela[3,4] and Milagros Medina[1,2,4*]

[1]Departamento de Bioquímica y Biología Molecular y Celular, Facultad de Ciencias, Universidad de Zaragoza, Spain.

[2]Instituto de Biocomputación y Física de Sistemas Complejos (BIFI), Universidad de Zaragoza, Spain.

[3]Estación Experimental de Aula Dei de Zaragoza, CSIC, Zaragoza, Spain.

[4]Group of Biochemistry, Biophysics and Computational Biology 'GBsC' (BIFI, Unizar) Joint Unit to CSIC.

SUPLEMENTARY TABLES

Table SP1. List of *Brucella* and alpha-proteobacteria evaluated in the context of *B. ovis* flavoproteins.

Brucellae species/strains	Pathogenic and Disease (host)	Alpha-proteobacteria [1]	Function / Disease
***Brucella abortus* 2308**	Yes / ovine brucellosis (zoonosis, veal, camel, buffalo)	***Agrobacterium tumefaciens* now *Rhizobium radiobacter***	biotechnological applications
***Brucella canis* ATCC 23365**	Yes / brucellosis (zoonosis, dog)	***Anaplasma phagocytophilum***	causative agent of granulocytic anaplasmosis
Brucella ceti	Yes /brucellosis (zoonosis, cetaceans, seals)	***Azorhizobium caulinodans***	nitrogen-fixing symbiotic bacteria in plants (*Sesbania* species)
Brucella inopinata	Yes / brucellosis	***Azotobacter vinelandii***	nitrogen-fixing symbiotic bacteria -biotechnology application: produces alginate)
Brucella intermedia or Ochrobactrum intermedium* [2] *or Brucella ciceri	Yes / brucellosis	***Bartonella quintana***	causative agent of louse-born trench fever during World War I, causing deer ked dermatitis in humans
***Brucella melitensis* bv. 1 str. 16M**	Yes / ovine brucellosis (zoonosis, sheep, goats)	***Bartonella schoenbuchensis***	causative agent of bacteraemia in ruminants
***Brucella microti* CCM 4915**	Yes / brucellosis (red fox)	***Bradyrhizobium diazoefficiens***	nitrogen-fixing symbiotic bacteria -biotechnology application: produces alginate)
Brucella neotomae	Yes / brucellosis (rodents)	***Candidatus pelagibacter***	marine bacterium dominant in ocean surface waters (possibly the most numerous bacteria in the world)
***Brucella ovis* ATCC 25840**	Yes / ovine brucellosis (sheep)	***Caulobacter crescentus***	model for regulation of cell cycle and cellular differentiation [3]
Brucella_pinnipedialis		***Ehrlichia chaffeensis***	Human Monocytic Ehrlichiosis
***Brucella* sp. NVSL 07-0026**		***Gluconobacter oxydans***	biotechnological applications
***Brucella* sp. 10RB9215**		***Mesorhizobium loti***	nitrogen-fixing symbiotic bacteria in plants (*Lotus* species)
***Brucella* sp. BO3**		***Paracoccus yeei***	peritonitis - opportunistic bacterium in ambulatory peritoneal dialysis
***Brucella* sp. 6810**		***Phenylobacterium zucineum***	associated to human leukaemia
***Brucella* sp. 2280**		***Rhizobium etli***	nitrogen-fixing symbiotic bacteria in plants (*Phaseolus vulgaris*)
***Brucella* sp. 09RB8471**		***Rhizobium leguminosarum***	nitrogen-fixing symbiotic bacteria in plants (*Fabae* family)
***Brucella* sp. 09RB8910**		***Rhodobacter capsulatus***	model purple photosynthetic bacteria, biotechnological applications
***Brucella* sp. 2002734562**		***Rhodobacter sphaeroides* now *Cereibacter sphaeroides***	model purple photosynthetic bacteria, biotechnological applications
***Brucella suis* bv. 4 str. 40**	Yes / brucellosis (zoonosis, hare, pork, reindeer)	***Rhodopseudomonas palustris***	purple non-sulphur bacteria - switch between four different modes of metabolism
Brucella vulpis	Yes / brucellosis (red fox)	***Rickettsia rickettsii***	causative agent of Rocky Mountain Spotted Fever
		Rickettsia typhi	causative agent of murine typhus
		Ruegeria pomeroyi	model marine bacteria
		Sinorhizobium meliloti	nitrogen-fixing symbiotic bacteria in plants (*Medicago sativa, Medicago truncatula*)
		Sphingomonas paucimobilis	human infections
		Wolbachia pipientis	causative agent of Dengue
		Xanthobacter autotrophicus	biotreatment in wastewater (dichloroethane and toluene biodegradation)

Table SP2. PDB entries available for *Brucella ovis* ATCC 25840 proteins. Equally coloured PDB lines correspond to different entries of the same protein.

PDB ID	Protein Name (potential function)	Gene	Flavin	NAD(P)/H-binding /ligand in structure	Functional Classification
6RR3	Ferredoxin-$NADP^+$reductase	BOV_RS01770	FAD	Yes/-	oxidoreductase
6RRA	Ferredoxin-$NADP^+$reductase:$NADP^+$ complex	BOV_RS01770	FAD	Yes/$NADP^+$	oxidoreductase
4X54	Putative short chain dehydrogenase/reductase	BOV_RS00560	-	Yes/-	oxidoreductase
5EL0	Putative short chain dehydrogenase/reductase:NAD^+ complex	BOV_RS00560	-	Yes/NAD^+	oxidoreductase
5ER6	Putative short chain dehydrogenase/reductase	BOV_RS10490	-	Yes/-	oxidoreductase
4Z0T	Putative oxoacyl-(acyl carrier protein) reductase	BOV_RS02275	-	Yes/-	oxidoreductase
5HA5[a]	Putative NAD-dependent oxidoreductase	ENT92748.1	-	Yes/NAD^+	oxidoreductase
5DWM	Phosphinothricin N-acetyltransferase	BOV_RS00430	-	No/-	Transferase
5DWN	Phosphinothricin N-acetyltransferase:AcetylCoA complex	BOV_RS00430	-	No/AcetylCoA	Transferase
5VM1	Xyloylose kinase	xylB	-	No/-	Transferase
4WOK	UDP-glucose 4-epimerase:NAD^+ complex	galE	-	Yes/NAD^+	Isomerase
7JFN	Leucine-, isoleucine-, valine-, threonine-, and alanine- ABC transporter/periplasmic (closed conformation)	BOV_RS10455	-	No/-	Transport protein
4XFK	Leucine-, isoleucine-, valine-, threonine-, and alanine- ABC transporter/periplasmic	BOV_RS10455	-	No/-	Transport protein
4Z9N	ABC transporter/periplasmic binding protein:Glutathione complex	BOV_RS03670	-	No/glutathione	Transport protein

[a]This PDB corresponds to a protein from *B. ovis* IntaBari-2002-82-58 that shares 100% identity with the reference strain here used.

Table SP3. Summary of structures found in the PDB of proteins of the genus *Brucella* that putatively bind flavin cofactors.

PDB ID (Uniprot ID)	Protein	Flavin	$NAD(P)^+$/H-binding /ligands in structure	Species Strain	*B. ovis* ATCC 25840 gene	% Identity[a]	Functional Classification
5F4B (Q2YQ23)	$NAD(P)^+$/H (quinone) dehydrogenase	FMN	$NAD(P)^+$/-	*B. abortus* 2308	BOV_RS05025	98.49	oxidoreductase
4W9U (Q2YPZ4)	Acyl-CoA dehydrogenase	fad [b]	-	*B. abortus* 2308	ENT94890.1 4W9U[c]	100	oxidoreductase
4U83 (WP_076773354.1)	Putative Acyl-CoA dehydrogenase	fad[b]	-	*B. abortus* A13334	BOV_RS12330	99.20	oxidoreductase
4JNQ (Q8YID2)	Thioredoxin reductase	FADH2[d]	$NADP^+$/-	*B. melitensis* bv. 1 str. 16M	BOV_RS07155 (trxB)	99.69	oxidoreductase
5EZ3 (Q8YC61)	Acyl-CoA dehydrogenase	FAD	-	*B. melitensis* bv. 1 str. 16M	BOV_RS13205	99.64	oxidoreductase
4IRA/3CB0 (Q8YHT7)	Co(II)rrin reductase activity (CobR)	FAD/FMN	-	*B. melitensis* bv. 1 str. 16M	BOV_RS06210	98.84	oxidoreductase
4O5M (A0A0H3G544)	Isovaleryl-CoA dehydrogenase	FAD	-	*B. suis* bv. 1, 1330	BOV_RS00090 (ivd)	99.74	oxidoreductase
4E0F (Q2YN92)	Riboflavin synthase	RF	-/RoSF, PyrM[e]	*B. abortus* 2308	BOV_RS03790 (ribE)	98.51	transferase
3T50 (Q8YC53)	LOV-PAS construct of sensory protein Blue-light-activated histidine kinase	FMN	-	*B. melitensis* bv. 1 str. 16M	BOV_RS13160	99.80	signalling protein (transferase)
6PPS (Q2YKK7)	LOV domain of sensory protein Blue-light-activated histidine kinase	FMN	-	*B. abortus* 2308	BOV_RS13160	99.80	signalling protein (transferase)
3U0I (Q2YQN9)	FAD-binding protein	fad[b]	-/NHE[f]	*B. abortus* 2308	BOV_RS06575	99.31	flavoprotein

[a] Identity versus *B. ovis* ATCC 25840 (reference strain). [b] Lower cases indicate flavin cofactor binding is predicted, but no flavin is observed in the PDB structure.
[c] Not found in *B. ovis* ATCC 25840, but one of the domains is equivalent in *B. ovis* IntaBari-2002-82-58. [d] FDH2, Dihydroflavine-adenine dinucleotide. [e] RoSF, roseoflavine. [f] NHE, 2-[N-cychlohexylamino]ethane sulfonic acid.

Table SP4. Gene context for the genes encoding selected flavoproteins in the *Brucella ovis* ATCC 25840 at chromosome I. The table summarizes for each flavoprotein gene (highlighted in orange), the gene reading direction, the gene name and the name of expected produced protein. Similar data are also presented for upstream and downstream genes. Genes identified as transposases or pseudogenes are highlighted in grey and green respectively, and those codifying for tRNAs are shown in violet.

Up Stream		Flavoenzyme Gene Code		Down Stream	
5' ← 3'<br>BOV_RS00080<br>Biotin carboxylase	5' ← 3'<br>BOV_RS00085<br>Carboxyl transferase family protein	5' ← 3'<br>BOV_RS00090<br>ivd: Isovaleryl-CoA dehydrogenase	5' ← 3'<br>BOV_RS00095<br>Acetoacetate-CoA ligase	5' ← 3'<br>BOV_RS00100<br>CHRD domain-containing protein	
5' → 3'<br>BOV_RS00940<br>Response regulator transcription factor	5' → 3'<br>BOV_RS00945<br>DeoR/GlpR transcriptional regulator	5' → 3'<br>BOV_RS00950<br>glpD: Glycerol-3-phosphate dehydrogenase	5' → 3'<br>BOV_RS00955<br>Sigma-54-dependent Fis family transcriptional regulator	5' → 3'<br>BOV_RS00960<br>Aldehyde dehydrogenase family protein	
5' ← 3'<br>BOV_RS01010<br>thiG: Thiazole synthase	5' ← 3'<br>BOV_RS01015<br>thiS: Thiamine biosynthesis protein	5' ← 3'<br>BOV_RS01020<br>thiO: Glycine oxidase	5' ← 3'<br>BOV_RS01025<br>thiD: Phosphomethylpyrimidine kinase	5' → 3'<br>BOV_RS01030<br>Sensory box/GGDEF domain/EAL domain protein	
5' → 3'<br>BOV_RS01075<br>SoxB_1: Sarcosine oxidase beta subunit	5' ← 3'<br>BOV_RS17650<br>IS5 family transposase	5' → 3'<br>BOV_RS01090<br>SoxB_2: Sarcosine oxidase, beta subunit	5' → 3'<br>BOV_RS01095<br>SoxD: Sarcosine oxidase, delta subunit	5' → 3'<br>BOV_RS01100<br>SoxA: Sarcosine oxidase alpha subunit	
5' → 3'<br>BOV_RS01505<br>MutT/nudix family protein	5' ← 3'<br>BOV_RS01510<br>preA: NAD-dependent dihydropyrimidine dehydrogenase	5' ← 3'<br>BOV_RS01515<br>preT: Putative pyridine nucleotide-disulphide oxidoreductase/ Dihydropyrimidine dehydrogenase	5' ← 3'<br>BOV_RS01520<br>Uncharacterized protein	5' → 3'<br>BOV_RS01525<br>pgi: Glucose-6-phosphate isomerase	
5' → 3'<br>BOV_RS01645<br>SCP domain-containing protein	5' → 3'<br>BOV_RS01650<br>Probable multidrug resistance protein NorM	5' ← 3'<br>BOV_RS01655<br>PyrD: dihydroorotate dehydrogenase 2 (quinone)	5' ← 3'<br>BOV_RS01660<br>DUF952 domain-containing protein	5' ← 3'<br>BOV_RS17660<br>IS5-like element IS711 family transposase	
5' → 3'<br>BOV_RS01760<br>panC: Pantothenate synthetase	5' → 3'<br>BOV_RS01765<br>panB: 3-methyl-2-oxobutanoate hydroxymethyltransferase	5' ← 3'<br>BOV_RS01770<br>fpr: Ferredoxin-NADP+ reductase	5' ← 3'<br>BOV_RS01775<br>Fe-superoxide dismutase	5' → 3'<br>BOV_RS01780<br>nspC: Carboxynorspermidine/ carboxyspermidine decarboxylase	
5' ← 3'<br>BOV_RS01835<br>Chitooligosaccharide deacetylase	5' → 3'<br>BOV_RS01840<br>Uncharacterized protein	5' → 3'<br>BOV_RS01845<br>xdhA: Xanthine dehydrogenase, small subunit	5' → 3'<br>BOV_RS01850<br>PSEUDOGEN xdhB	5' → 3'<br>BOV_RS01855<br>xdhC: Xanthine dehydrogenase accessory protein	
5' → 3'<br>BOV_RS02085<br>glyS: Glycine--tRNA ligase beta subunit	5' → 3'<br>BOV_RS02090<br>Threonylcarbamoyl-AMP synthase	5' → 3'<br>BOV_RS02095<br>Potential FAD-binding oxygen oxidoreductase (glcE?)	5' ← 3'<br>BOV_RS02100<br>Uncharacterized protein	5' ← 3'<br>BOV_RS02105<br>RHH_1 domain-containing protein	
5' ← 3'<br>BOV_RS02110<br>Uncharacterized protein	5' ← 3'<br>BOV_RS02115<br>Enoyl-CoA hydratase/isomerase family protein	5' ← 3'<br>BOV_RS02120<br>Short Chain Acyl-CoA dehydrogenase	5' → 3'<br>BOV_RS02125<br>Putative glyoxalase	5' ← 3'<br>BOV_RS02130<br>purD: Phosphoribosylamine-glycine ligase	
5' ← 3'<br>BOV_RS02130<br>purD: Phosphoribosylamine-glycine ligase	5' → 3'<br>BOV_RS02135<br>ubiA: 4-hydroxybenzoate octaprenyltransferase	5' ← 3'<br>BOV_RS02140<br>pdxH: Pyridoxamine 5'-phosphate oxidase	5' → 3'<br>BOV_RS02145<br>17kDa_Anti_2 domain-containing protein	5' → 3'<br>BOV_RS02150<br>Putative chaperone protein DnaJ	
5' → 3'<br>BOV_RS02180<br>DUF374 domain-containing protein	5' → 3'<br>BOV_RS02185<br>Uncharacterized protein	5' → 3'<br>BOV_RS02190<br>aroC: Chorismate synthase	5' → 3'<br>BOV_RS02195<br>ribAB: 3,4-dihydroxy-2-butanone 4-phosphate synthase	5' → 3'<br>BOV_RS02200<br>Histone deacetylase family protein	
5' ← 3'	5' ← 3'	5' ← 3'	5' ← 3'	5' → 3'	

BOV_RS02755<br>Transcriptional regulator, AraC family	BOV_RS02760<br>betB: Betaine aldehyde dehydrogenase	BOV_RS02765<br>betA: Choline dehydrogenase (Glucose-methanol-choline GMC family)	BOV_RS02770<br>betI: Transcriptional regulator	BOV_RS02775<br>Protein coding asparaginase	
5' ← 3'<br>BOV_RS03090<br>Major facilitator family transporter	5' ← 3'<br>BOV_RS03095<br>Type-4 uracil-DNA glycosylase	5' → 3'<br>BOV_RS03100<br>Electron transferring flavoprotein-ubiquinone oxidoreductase (ETF-QO)	5' ← 3'<br>BOV_RS03105<br>AP endonuclease family 1 domain protein	5' ← 3'<br>BOV_RS03110<br>AMP nucleosidase	
5' → 3'<br>BOV_RS03680<br>Amino acid ABC transporter, permease protein	5' → 3'<br>BOV_RS03685<br>Amino acid ABC transporter, ATP-binding protein	5' ← 3'<br>BOV_RS03690<br>Predicted Salicylate 1-monooxygenase	5' ← 3'<br>BOV_RS03695<br>zf-CHCC domain-containing protein	5' → 3'<br>BOV_RS03700<br>ppk: Polyphosphate kinase	
5' → 3'<br>BOV_RS03780<br>Transcriptional repressor NrdR	5' → 3'<br>BOV_RS03785<br>ribD: Riboflavin biosynthesis protein	5' → 3'<br>BOV_RS03790<br>ribE: Riboflavin synthase alpha subunit	5' → 3'<br>BOV_RS03795<br>ribH: 6,7-dimethyl-8-ribityllumazine synthase	5' → 3'<br>BOV_RS03800<br>Transcription antitermination protein NusB	
5' → 3'<br>BOV_RS03990<br>nuoD	5' → 3'<br>BOV_RS03995<br>nuoE	5' → 3'<br>BOV_RS04000<br>nuoF	5' → 3'<br>BOV_RS04005<br>nuoG	5' → 3'<br>BOV_RS04010<br>nuoH	
5' ← 3'<br>BOV_RS04245<br>Amino acid ABC transporter, periplasmic amino acid-binding protein	5' → 3'<br>BOV_RS04250<br>Uncharacterized protein	5' → 3'<br>BOV_RS04255<br>dusA: tRNA dihydrouridine20/20a synthase	5' → 3'<br>BOV_RS04260<br>Uncharacterized protein	5' ← 3'<br>BOV_RS04265<br>cosB: Adenosylcobinamide-GDP ribazoletransferase	
5' → 3'<br>BOV_RS04415<br>Uncharacterized protein	5' → 3'<br>BOV_RS04420<br>Uncharacterized protein	5' ← 3'<br>BOV_RS04425<br>trmFO: Predicted Flavin-containing monooxygenase /Salicylate hydroxylase	5' ← 3'<br>BOV_RS04430<br>Uncharacterized protein	5' → 3'<br>BOV_RS04435<br>PSEUDOGEN	
5' ← 3'<br>BOV_RS04713<br>moaA: GTP 3',8-cyclase	5' → 3'<br>BOV_RS04714<br>GBBH-like_N domain-containing protein	5' ← 3'<br>BOV_RS04715<br>Predicted Salicylate hydroxylase	5' ← 3'<br>BOV_RS04720<br>fumB: Fumarate hydratase class I	5' → 3'<br>BOV_RS04725<br>Cell wall degradation protein	
5' → 3'<br>BOV_RS04840<br>ripA: Ribose-5-phosphate isomerase A	5' → 3'<br>BOV_RS04845<br>DUF2059 domain-containing protein	5' → 3'<br>BOV_RS04850<br>gor: Glutathione-disulphide reductase	5' → 3'<br>BOV_RS04855<br>Phospho-2-dehydro-3-deoxyheptonate aldolase	5' → 3'<br>BOV_RS04860<br>dgkA: Diacylglycerol kinase	
5' → 3'<br>BOV_RS04915<br>BOV_0991<br>PSEUDOGENE	5' → 3'<br>BOV_RS04920<br>BOV_0992<br>Ferredoxin, 2Fe-2S	5' → 3'<br>BOV_RS04925<br>BOV_0993<br>Predicted thioredoxin-disulphide reductase	5' ← 3'<br>BOV_RS04930<br>BOV_0994<br>Uncharacterized protein	5' → 3'<br>BOV_RS04935<br>BOV_0995<br>folP: Dihydropteroate synthase	
5' ← 3'<br>BOV_RS04975<br>Proline-rich region:Proline-rich extensin. FliO/MopB family protein	5' → 3'<br>BOV_RS04980<br>PAS domain-containing hybrid sensor histidine kinase/response regulator	5' → 3'<br>BOV_RS04985<br>Potential aminoacetone oxidase family FAD-binding enzyme / NAD(P)/FAD-dependent dehydrogenase	5' → 3'<br>BOV_RS04990<br>Serine hydrolase	5' ← 3'<br>BOV_RS17720<br>PSEUDOGENE	5' ← 3'<br>BOV_RS05000<br>vitamin B12-dependent ribonucleotide reductase
5' ← 3'<br>BOV_RS05015<br>Usp domain-containing protein	5' ← 3'<br>BOV_RS05020<br>CinA-related protein	5' → 3'<br>BOV_RS05025<br>wrpA: NAD(P)H dehydrogenase (quinone)	5' → 3'<br>BOV_RS05030<br>tRNA-Leu	5' → 3'<br>BOV_RS05040<br>PSEUDOGENE ilvA	
5' ← 3'<br>BOV_RS05115<br>galE-2: UDP-glucose 4-epimerase	5' → 3'<br>BOV_RS05120<br>MazG family protein	5' ← 3'<br>BOV_RS05125<br>Flavin reductase domain containing protein	5' ← 3'<br>BOV_RS05130<br>Putative NAD(P)H nitroreductase	5' → 3'<br>BOV_RS05135<br>Uncharacterized protein	
5' ← 3'<br>BOV_RS05345	5' ← 3'<br>BOV_RS05350	5' ← 3'<br>BOV_RS05355	5' → 3'<br>BOV_RS05360	5' → 3'<br>BOV_RS05365	

ntrC: DNA-binding transcriptional regulator	ntrB: Histidine kinase	nifR3 or DusB: tRNA dihydrouridine synthase B	Bifunctional enzyme IspD/IspF	Competence/damage-inducible protein CinA	
5' ← 3'<br>BOV_RS05380<br>lipA: Lipoyl synthase	5' ← 3'<br>BOV_RS05385<br>Uncharacterized protein	5' ← 3'<br>BOV_RS05390<br>lpdA-2: Dihydrolipoyl dehydrogenase	5' ← 3'<br>BOV_RS05395<br>Acetyltransferase component of pyruvate dehydrogenase complex	5' ← 3'<br>BOV_RS05400<br>pdhB: Pyruvate dehydrogenase E1 component subunit beta	
5' ← 3'<br>BOV_RS06200<br>Putative lipoprotein	5' ← 3'<br>BOV_RS06205<br>PSEUDOGEN	5' ← 3'<br>BOV_RS06210<br>cobR: Cob(II)alamin reductase	5' → 3'<br>BOV_RS06215<br>cobD-1: Cobalamin biosynthesis protein	5' ← 3'<br>BOV_RS06220<br>Putative branched-chain amino acid ABC transporter, periplasmic amino acid-binding protein	
5' ← 3'<br>BOV_RS06305<br>Uncharacterized protein	5' ← 3'<br>BOV_RS16680<br>cobQ: Cobyric acid synthase	5' → 3'<br>BOV_RS06310<br>Acyl-CoA dehydrogenase	5' → 3'<br>BOV_RS06315<br>mmsB: 3-hydroxyisobutyrate dehydrogenase	5' ← 3'<br>BOV_RS16685<br>Uncharacterized protein	
5' ← 3'<br>BOV_RS06565<br>Fluoride efflux transporter CrcB	5' → 3'<br>BOV_RS06570<br>Hypothetical protein	5' ← 3'<br>BOV_RS06575<br>Pyridoxamine 5'-phosphate oxidase family protein	5' → 3'<br>BOV_RS17790<br>IS5 family transposase	5' ← 3'<br>BOV_RS06590<br>PhzF family phenazine biosynthesis protein	
5' ← 3'<br>BOV_RS06645<br>Uncharacterized protein	5' ← 3'<br>BOV_RS06650<br>ilvN: Acetolactate synthase	5' ← 3'<br>BOV_RS06655<br>ilvB: Acetolactate synthase 3 catalytic subunit	5' ← 3'<br>BOV_RS06660<br>miaA: tRNA dimethylallyltransferase	5' → 3'<br>BOV_RS06665<br>serB: Phosphoserine phosphatase	
5' ← 3'<br>BOV_RS06660<br>tRNA (adenosine(37)-N6)-dimethylallyltransferase MiaA	5' → 3'<br>BOV_RS06665<br>Phosphoserine phosphatase SerB	5' ← 3'<br>BOV_RS06670<br>Potential aminoacetone oxidase family FAD-binding enzyme / NAD(P)/FAD-dependent dehydrogenase	5' ← 3'<br>BOV_RS06675<br>DegQ family serine endoprotease	5' ← 3'<br>BOV_RS06680<br>DUF2065 domain-containing protein	
5' ← 3'<br>BOV_RS06740<br>AsmA family protein	5' ← 3'<br>BOV_RS17795<br>Hypothetical protein	5' ← 3'<br>BOV_RS06750<br>Potential FAD-binding oxygen oxidoreductase (glcE?)	5' ← 3'<br>BOV_RS06755<br>Heme-degrading domain-containing protein	5' → 3'<br>BOV_RS06760<br>Serine/threonine protein kinase	
5' ← 3'<br>BOV_RS06840<br>ftsQ: Cell division protein	5' ← 3'<br>BOV_RS06845<br>ddlB: D-alanine-D-alanine ligase	5' ← 3'<br>BOV_RS06850<br>murB: UDP-N-acetylmuramate dehydrogenase	5' ← 3'<br>BOV_RS06855<br>murC: UDP-N-acetylmuramate-L-alanine ligase	5' ← 3'<br>BOV_RS06860<br>murG: UDP-N-acetylglucosamine-N-acetylmuramyl-(pentapeptide) pyrophosphoryl-undecaprenol N-acetylglucosamine transferase	
5' → 3'<br>BOV_RS06935<br>Putative peptidoglycan-binding protein	5' → 3'<br>BOV_RS06940<br>Putative transporter	5' ← 3'<br>BOV_RS06945<br>metF: Methylenetetrahydrofolate reductase	5' ← 3'<br>BOV_RS06950<br>Transcriptional regulator, ArsR family	5' ← 3'<br>BOV_RS06955<br>DUF2293 domain-containing protein	
5' → 3'<br>BOV_RS16805<br>Uncharacterized protein	5' ← 3'<br>BOV_RS07150<br>Transcriptional regulator, LysR family	5' ← 3'<br>BOV_RS07155<br>trxB: Thioredoxin reductase	5' → 3'<br>BOV_RS07160<br>Trk system potassium uptake protein	5' → 3'<br>BOV_RS07165<br>lrp-1	
5' ← 3'<br>BOV_RS07765<br>Sugar ABC transporter ATP-binding protein	5' ← 3'<br>BOV_RS07770<br>Hypothetical PepSY domain-containing protein	5' → 3'<br>BOV_RS07775<br>Oxidoreductase GMC family Choline dehydrogenase	5' ← 3'<br>BOV_RS07780<br>tRNA-Met	5' ← 3'<br>BOV_RS07785<br>rRNA-5S ribosomal RNA	
5' ← 3'<br>BOV_RS08470<br>Branched-chain amino acid ABC transporter, permease protein	5' ← 3'<br>BOV_RS08475<br>4-hydroxyproline epimerase	5' ← 3'<br>BOV_RS08480<br>D-amino acid dehydrogenase small subunit	5' → 3'<br>BOV_RS08485<br>Transcriptional regulator, GntR family	5' ← 3'<br>BOV_RS08490<br>Transcriptional regulator, MarR family	

**5' ← 3'**<br>BOV_RS08960<br>Ammonium transporter	**5' ← 3'**<br>BOV_RS08965<br>tesB	**5' → 3'**<br>BOV_RS08970<br>Predicted UbiH/COQ6 monooxygenase family	**5' ← 3'**<br>BOV_RS08975<br>Glyoxalase family protein	**5' ← 3'**<br>BOV_RS08980<br>sdhB	
**5' ← 3'**<br>BOV_RS08975<br>Glyoxalase family protein	**5' ← 3'**<br>BOV_RS08980<br>sdhB: Succinate dehydrogenase iron-sulfur subunit	**5' ← 3'**<br>BOV_RS08985<br>sdhA: Succinate dehydrogenase flavoprotein subunit	**5' ← 3'**<br>BOV_RS08990<br>sdhD: Succinate dehydrogenase hydrophobic membrane anchor subunit	**5' ← 3'**<br>BOV_RS08995<br>Succinate dehydrogenase cytochrome b556 subunit	
**5' → 3'**<br>BOV_RS09055<br>xerC: Tyrosine recombinase	**5' → 3'**<br>BOV_RS09060<br>Uncharacterized protein	**5' ← 3'**<br>BOV_RS09065<br>lpdA-3: Dihydrolipoyl dehydrogenase	**5' ← 3'**<br>BOV_RS09070<br>Uncharacterized protein	**5' ← 3'**<br>BOV_RS09075<br>Transporter, LysE family	
**5' → 3'**<br>BOV_RS09285<br>cysK: Cysteine synthase	**5' ← 3'**<br>BOV_RS09290<br>3-hydroxybutyryl-CoA dehydrogenase	**5' ← 3'**<br>BOV_RS09295<br>etfA: Electron transfer flavoprotein, alpha subunit	**5' ← 3'**<br>BOV_RS09300<br>etfB: Electron transfer flavoprotein, beta subunit	**5' → 3'**<br>BOV_RS09305<br>queC: 7-cyano-7-deazaguanine synthase	
**5' ← 3'**<br>BOV_RS09685<br>Transcriptional regulator, LacI family	**5' → 3'**<br>BOV_RS09690<br>Uncharacterized protein	**5' ← 3'**<br>BOV_RS09695<br>Bacterial luciferase	**5' ← 3'**<br>BOV_RS17075<br>Putative ABC transporter, periplasmic substrate-binding protein	**5' → 3'**<br>BOV_RS09700<br>Branched-chain amino acid ABC transporter, permease protein	
**5' ← 3'**<br>BOV_RS09725<br>parA: Chromosome partitioning protein	**5' ← 3'**<br>BOV_RS09730<br>rsmG: Ribosomal RNA small subunit methyltransferase G	**5' ← 3'**<br>BOV_RS09735<br>mnmG: tRNA uridine 5-carboxymethylaminomethyl modification enzyme	**5' → 3'**<br>BOV_RS09736<br>mnmE: tRNA modification GTPase	**5' ← 3'**<br>BOV_RS09737<br>Thioredox_DsbH domain-containing protein	

Table SP5. Gene context for the genes encoding selected flavoproteins in the *Brucella ovis* ATCC 25840 at chromosome II. The table summarizes for each flavoprotein gene (highlighted in orange) the gene reading direction, the gene name and the name of expected produced protein. Similar data are also presented for upstream and downstream genes. Genes identified as transposases or pseudogenes are highlighted in grey and green respectively and those codifying for tRNAs are shown in violet.

Upstream		Flavoenzyme Gene Code		Downstream	
5' → 3'<br>BOV_RS10575<br>PSEUDOGENE	5' ← 3'<br>BOV_RS10580<br>PSEUDOGENE	5' → 3'<br>BOV_RS10585<br>gltB: Glutamate synthase large subunit	5' → 3'<br>BOV_RS10590<br>gltD: Glutamate synthase, small subunit	5' ← 3'<br>BOV_RS10595<br>Amino acid permease family protein	
5' ← 3'<br>BOV_RS11145<br>Transcriptional regulator, LysR family	5' ← 3'<br>BOV_RS17905<br>IS5-like element IS711 family transposase	5' → 3'<br>BOV_RS11160<br>glcD: Glycolate oxidase, subunit	5' → 3'<br>BOV_RS11165<br>PSEUDOGENE glcE	5' → 3'<br>BOV_RS17195<br>PSEUDOGENE	
5' ← 3'<br>BOV_RS11245<br>groES: Co-chaperone	5' → 3'<br>BOV_RS11250<br>PSEUDOGENE	5' → 3'<br>BOV_RS11255<br>ribF: Riboflavin biosynthesis protein	5' → 3'<br>BOV_RS11260<br>ileS: Isoleucine--tRNA ligase	5' → 3'<br>BOV_RS11265<br>Putative lipoprotein	
5' ← 3'<br>BOV_RS11380<br>PSEUDOGENE	5' → 3'<br>BOV_RS11385<br>CAIB/BAIF family protein	5' ← 3'<br>BOV_RS11390<br>NADPH dehydrogenase (Old yellow enzyme)	5' → 3'<br>BOV_RS11395<br>Bacterial regulatory protein, MerR family	5' ← 3'<br>BOV_RS11400<br>Sensor protein QseC	
5' → 3'<br>BOV_RS11420<br>PepSY domain-containing protein	5' → 3'<br>BOV_RS11425<br>hypothetical protein	5' → 3'<br>BOV_RS11430<br>flavodoxin domain-containing protein	5' → 3'<br>BOV_RS11435<br>hypothetical protein	5' → 3'<br>BOV_RS11440<br>FAD:protein FMN transferase	5' ← 3'<br>BOV_RS11445<br>Sensor domain-containing diguanylate cyclase
5' → 3'<br>BOV_RS11800<br>Putative transporter	5' → 3'<br>BOV_RS11805<br>Protein NrdH	5' → 3'<br>BOV_RS11810<br>Protein NrdI	5' → 3'<br>BOV_RS11815<br>Protein NrdE	5' → 3'<br>BOV_RS11820<br>Protein NrdF	
5' → 3'<br>BOV_RS12320<br>PSEUDOGENE	5' → 3'<br>BOV_RS12325<br>msrB: Peptide methionine sulfoxide reductase	5' ← 3'<br>BOV_RS12330<br>Acyl-CoA dehydrogenase	5' ← 3'<br>BOV_RS12335<br>Acetyl-CoA acetyltransferase	5' ← 3'<br>BOV_RS12340<br>3-hydroxyacyl-CoA dehydrogenase type II	
5' → 3'<br>BOV_RS12450<br>Uncharacterized protein	5' → 3'<br>BOV_RS12455<br>Uncharacterized protein	5' ← 3'<br>BOV_RS12460<br>NADH dehydrogenase	5' ← 3'<br>BOV_RS12465<br>Bacterial regulatory protein, MarR family	5' ← 3'<br>BOV_RS17935<br>Putative transposase for insertion sequence element IS6501	
5' ← 3'<br>BOV_RS12535<br>Uncharacterized protein	5' → 3'<br>BOV_RS12540<br>mogA: Molybdenum cofactor biosynthesis protein	5' ← 3'<br>BOV_RS12545<br>Predicted nitroreductase family protein	5' → 3'<br>BOV_RS17940<br>PSEUDOGENE	5' → 3'<br>BOV_RS12555<br>Monovalent cation/proton antiporter, MnhA/PhaA subunit	
5' → 3'<br>BOV_RS12660<br>Putative D-aminopeptidase	5' → 3'<br>BOV_RS12665<br>Putative cell division protein FtsK	5' → 3'<br>BOV_RS12670<br>lpdA-1: Dihydrolipoyl dehydrogenase	5' ← 3'<br>BOV_RS12675<br>SURF1-like protein	5' ← 3'<br>BOV_RS17325<br>Uncharacterized protein	
5' ← 3'<br>BOV_RS13070<br>PSEUDOGENE	5' ← 3'<br>BOV_RS13075<br>Phosphatidylcholine synthase	5' → 3'<br>BOV_RS13080<br>UbiH/UbiF family hydroxylase	5' ← 3'<br>BOV_RS13085<br>Hemimethylated DNA-binding region	5' ← 3'<br>BOV_RS13090<br>Invasion associated locus B (IalB) protein	
5' ← 3'<br>BOV_RS13150<br>tRNA	5' ← 3'<br>BOV_RS13155<br>Lectin-like protein BA14k	5' → 3'<br>BOV_RS13160<br>Blue-light-activated histidine kinase	5' → 3'<br>BOV_RS13165<br>lipB: Octanoyltransferase	5' ← 3'<br>BOV_RS13170<br>Putative membrane protein	
5' ← 3'<br>BOV_RS13195<br>gatC: Aspartyl/glutamyl-tRNA(Asn/Gln) amidotransferase subunit C	5' → 3'<br>BOV_RS13200<br>UPF0173 metal-dependent hydrolase	5' ← 3'<br>BOV_RS13205<br>Acyl-CoA dehydrogenase	5' → 3'<br>BOV_RS13210<br>Putative pre-16S rRNA nuclease	5' → 3'<br>BOV_RS13215<br>pyrB: Aspartate carbamoyltransferase	
5' → 3'<br>BOV_RS13335<br>Uncharacterized protein	5' ← 3'<br>BOV_RS13340<br>Transcriptional regulator, AraC family	5' → 3'<br>BOV_RS13345<br>Predicted D-amino acid dehydrogenase	5' ← 3'<br>BOV_RS13350<br>Amino acid ABC transporter, periplasmic amino acid-binding protein	5' ← 3'<br>BOV_RS13355<br>Amino acid ABC transporter, periplasmic amino acid-binding protein	
5' → 3'<br>BOV_RS13390<br>Putative transcriptional regulator PcaR	5' ← 3'<br>BOV_RS13395<br>PSEUDOGENE	5' → 3'<br>BOV_RS13400<br>pobA: 4-hydroxybenzoate 3-monooxygenase	5' ← 3'<br>BOV_RS13405<br>Pca operon transcription factor PcaQ	5' → 3'<br>BOV_RS13410<br>PSEUDOGENE	
5' → 3'<br>BOV_RS13520<br>Probable branched-chain-amino-acid aminotransferase	5' → 3'<br>BOV_RS13525<br>sulfite exporter TauE/SafE family protein. Transmembrane	5' → 3'<br>BOV_RS13530<br>Predicted KsdD-like steroid dehydrogenase	5' → 3'<br>BOV_RS13535<br>DUF3775 domain-containing protein	5' → 3'<br>BOV_RS13540<br>aldehyde dehydrogenase family protein	
5' ← 3'<br>BOV_RS13785<br>gcvT: Glycine cleavage system T protein	5' → 3'<br>BOV_RS13790<br>Uncharacterized protein	5' ← 3'<br>BOV_RS13795<br>Predicted Ferredoxin/rubedroxin/ putidaredoxin NAD+ Reductase	5' ← 3'<br>BOV_RS13800<br>MurR/RpiR family transcriptional regulator	5' → 3'<br>BOV_RS13805<br>N-formylglutamate amidohydrolase	
5' → 3'<br>BOV_RS13960	5' → 3'<br>BOV_RS13965	5' → 3'<br>BOV_RS13970	5' ← 3'<br>BOV_RS18005	5' → 3'<br>BOV_RS18010	

Glutamine synthetase family protein	Uncharacterized protein	Predicted D-amino acid dehydrogenase	Transcriptional regulator, MarR family	IS711 transposase orfA	
5' ← 3'<br>BOV_RS14110<br>Putative fatty oxidation complex, beta subunit	5' → 3'<br>BOV_RS14115<br>Acyl-CoA dehydrogenase	5' ← 3'<br>BOV_RS14120<br>Uncharacterized protein	5' → 3'<br>BOV_RS14130<br>Putative transcriptional regulator, LysR family	5' → 3'<br>BOV_RS14135<br>Acyl-CoA dehydrogenase	5' ← 3'<br>BOV_RS14140<br>nikE: Nickel import ATP-binding protein
5' ← 3'<br>BOV_RS1475<br>PSEUDOGENE	5' ← 3'<br>BOV_RS14280<br>Uncharacterized protein	5' ← 3'<br>BOV_RS14290<br>Nitronate monooxygenase (formerly 2-nitropropane dioxygenase NPD)	5' → 3'<br>BOV_RS14295<br>tRNA	5' → 3'<br>BOV_RS14300<br>SocA family protein	
5' ← 3'<br>BOV_RS14395<br>PSEUDOGENE	5' ← 3'<br>BOV_RS14400<br>EamA family transporter Transporter, DME family, Lipid Biosynthesis	5' ← 3'<br>BOV_RS14405<br>Potential L-gulonolactone oxidase	5' ← 3'<br>BOV_RS14415<br>UbiA prenyltransferase family. Transfer of alkyls different from methyl	5' ← 3'<br>BOV_RS14415<br>DeoR/GlpR transcriptional regulator	
5' → 3'<br>BOV_RS18245<br>Hypothetical protein	5' → 3'<br>BOV_RS14445<br>Carbohydrate kinase/eryA	5' → 3'<br>BOV_RS14450<br>eryB	5' → 3'<br>BOV_RS14455<br>eryC Aminotransferase	5' → 3'<br>BOV_RS14460<br>PSEUDOGENE	
5' → 3'<br>BOV_RS14620<br>Transcriptional regulator, Lrp/AsnC family	5' ← 3'<br>BOV_RS14625<br>Alkene reductase: N-ethylmaleimide reductase (Glycerol trinitrate reductase or xenobiotic reductase B)	5' ← 3'<br>BOV_RS14630<br>Transcriptional regulator, ArsR family. Helix-turn-helix	5' → 3'<br>BOV_RS14635<br>PSEUDOGENE	5' ← 3'<br>BOV_RS14640<br>Predicted monomeric Sarcosine oxidase	5' ← 3'<br>BOV_RS14645<br>PSEUDOGENE
5' ← 3'<br>BOV_RS14705<br>Uncharacterized protein	5' ← 3'<br>BOV_RS14710<br>PSEUDOGENE	5' → 3'<br>BOV_RS14715<br>lldD: L-lactate dehydrogenase (cytochrome c o b2)	5' → 3'<br>BOV_RS14720<br>Outer surface protein	5' ← 3'<br>BOV_RS14725<br>Putative alanine catabolic operon transcriptional regulator	
5' ← 3'<br>BOV_RS14725<br>Putative alanine catabolic operon transcriptional regulator	5' → 3'<br>BOV_RS14730<br>alr: Alanine racemase	5' → 3'<br>BOV_RS14735<br>dadA: D-amino acid dehydrogenase	5' ← 3'<br>BOV_RS14740<br>Putative transcriptional regulator	5' → 3'<br>BOV_RS14745<br>Putative omega-amino acid--pyruvate aminotransferase	
5' → 3'<br>BOV_RS14900<br>Branched-chain amino acid ABC transporter, permease protein	5' → 3'<br>BOV_RS14900<br>PSEUDOGENE	5' → 3'<br>BOV_RS14905<br>Choline dehydrogenase (GMC family, membrane bound)	5' → 3'<br>BOV_RS14910<br>MetQ/NlpA family ABC transporter substrate-binding protein	5' → 3'<br>BOV_RS17565<br>PSEUDOGENE	
5' → 3'<br>BOV_RS15060<br>G-protein beta WD-40 repeat:ATP/GTP-binding site motif A	5' ← 3'<br>BOV_RS15065<br>Esterase/lipase/thioesterase, active site	5' → 3'<br>BOV_RS15070<br>msrP: Protein-methionine-sulfoxide reductase catalytic subunit	5' → 3'<br>BOV_RS15075<br>msrQ: Protein-methionine-sulfoxide reductase heme-binding subunit	5' ← 3'<br>BOV_RS15080<br>Endoribonuclease L-PSP	
5' ← 3'<br>BOV_RS15154<br>Uncharacterized protein	5' ← 3'<br>BOV_RS15150<br>GGDEF domain protein	5' ← 3'<br>BOV_RS15155<br>lhgO: L-2-hydroxyglutarate oxidase	5' → 3'<br>BOV_RS15160<br>Putative exonuclease	5' ← 3'<br>BOV_RS18050<br>Amidase	
5' → 3'<br>BOV_RS15380<br>tRNA-Leu	5' → 3'<br>BOV_RS15385<br>Amidohydro_3 domain-containing protein	5' → 3'<br>BOV_RS15390<br>bluB: Predicted aerobic 5,6-dimethylbenzimidazole synthase	5' → 3'<br>BOV_RS15395<br>Universal stress protein family	5' → 3'<br>BOV_RS15400<br>Putative membrane protein	
5' → 3'<br>BOV_RS15420<br>Uncharacterized protein	5' → 3'<br>BOV_RS15425<br>prfC: Peptide chain release factor 3	5' ← 3'<br>BOV_RS15430<br>coaBC: Coenzyme A biosynthesis bifunctional protein: Phosphopantothenoylcysteine decarboxylase / Phosphopantothenate-cysteine ligase (CTP)	5' ← 3'<br>BOV_RS15435<br>ubiB: 2-polyprenylphenol 6-hydroxylase	5' ← 3'<br>BOV_RS15440<br>ubiE: Ubiquinone/menaquinone biosynthesis C-methyltransferase	

Table SP6. *Brucella ovis* ATCC 25840 flavoproteins and flavoenzymes found within the 20 *Brucella* and 26 alpha-proteobacteria of Table SP1.

EC	Protein	Protein Code	*Brucella* content / 20 species	Alpha-proteobacteria content / 26 species [b]	Core protein in *Brucella* [c]
1.1.5.3	Glycerol-3-phosphate dehydrogenase	ABQ60174.1	20	12, 1-2x	Y
1.1.1.402	D-erythritol 1-phosphate dehydrogenase	ABQ62056.1	17	10, 2-2x, 1-3x	N
1.1.2.3	L-lactate dehydrogenase (cytochrome c o b2)	ABQ62635.1	20	21	Y
1.1.3.8	Potential L-gulonolactone oxidase FAD-binding oxygen oxidoreductase	ABQ62001.1	15	15	N
1.1.99.1	Choline dehydrogenase (Glucose-methanol-choline GMC family)	ABQ61350.1	18	18	N
1.1.99.1	Choline dehydrogenase (GMC family)	ABQ60630.1	14	10, 1-2x	N
1.1.99.1	Choline dehydrogenase (GMC family, membrane bound)	ABQ62100.1	14	16	N
1.1.99.2	Predicted L-2-hydroxyglutarate dehydrogenase	ABQ62911.1	16	14, 3-2x	N
1.1.99.14	Glycolate dehydrogenase GlcD subunit	ABQ62237.1	17	16, 4-2x	N
1.1.-.-[a]	Potential FAD-binding oxygen oxidoreductase (glcE?)	ABQ60928.1	19	17	N
1.1-.-[a]	Potential FAD-binding oxygen oxidoreductase (glcE?)	ABQ61939.1	20	16	Y
1.3.1.1/2	NADH dependent Dihydropyrimidine dehydrogenase subunit PreA	ABQ60560.1	20	10 , 1-2x	Y
	NADH dependent Dihydropyrimidine dehydrogenase subunit PreT	ABQ61103.1	20	11	Y
1.3.5.2	PyrD dihydroorotate dehydrogenase 2 (quinone)	ABQ61413.1	20	24	Y
1.3.1.88	tRNA dihydrouridine synthase B	ABQ61416.1	20	22 , 1-2x	Y
1.3.1.91	tRNA dihydrouridine20/20a synthase	ABQ61966.1	20	17, 2-2x	Y
1.3.1.98	UDP-N-acetylmuramate dehydrogenase	ABQ61769.1	20	25	Y
1.3.1.-[a] /1.7.1.B1	Predicted alkene reductase: N-ethylmaleimide reductase, glycerol trinitrate reductase or xenobiotic reductase B	ABQ62490.1	15	18	N
1.3.5.1	Succinate dehydrogenase flavoprotein subunit	ABQ61077.1	20	**26**	Y
1.3.8.4	Isovaleryl-CoA dehydrogenase	ABQ60382.1	20	15, 2-2x, 1-3x	Y
1.3.8.-[a]	Acyl-CoA dehydrogenase	ABQ62576.1	19	13	N
1.3.8.1	Short Chain Acyl-CoA dehydrogenase	ABQ60180.1	19	14, 1-2x	N
1.3.8.-[a]	Acyl-CoA dehydrogenase	ABQ61585.1	18	14, 1-2x	N
1.3.8.-[a]	Acyl-CoA dehydrogenase	ABQ62784.1	19	9, 2-2x	N
1.3.8.-[a]	Acyl-CoA dehydrogenase	ABQ62889.1	18	12	N
1.3.8.-[a]	Acyl-CoA dehydrogenase	ABQ62082.1	18	17, 1-2x	N
1.3.99.-[a]	Predicted KsdD-like steroid dehydrogenase	ABQ62061.1	17	11	N

1.4.1.13	Glutamate synthase large subunit (alpha subunit)	ABQ61996.1	20	15, 1-2x	Y
	Glutamate synthase small subunit (beta subunit)	ABQ62546.1	11	11	N
1.4.3.5	Pyridoxamine 5'-phosphate oxidase	ABQ60142.1	20	20	Y
1.4.-.-[a]	Pyridoxamine 5'-phosphate oxidase family protein	ABQ61684.1	19	5	N
1.4.3.19	Glycine oxidase ThiO	ABQ60316.1	20	19	Y
1.4.3.-[a]	Potential Aminoacetone oxidase family FAD-binding enzyme/ NAD(P)/FAD-dependent dehydrogenase	ABQ60616.1	20	15, 1-2x	Y
1.4.3.-[a]	Potential Aminoacetone oxidase family FAD-binding enzyme/ NAD(P)/FAD-dependent dehydrogenase	ABQ60524.1	20	15, 1-2x	Y
1.4.99.-[a]	Predicted D-amino acid dehydrogenase small subunit	ABQ61937.1	20	11, 1-2x	Y
1.4.99.-[a]	D-amino acid dehydrogenase	ABQ62278.1	20	11, 3-2x	Y
1.4.-.-[a]	Predicted D-amino acid dehydrogenase	ABQ62519.1	19	8, 1-2x	N
1.4.-.-[a]	Predicted D-amino acid dehydrogenase	ABQ62405.1	20	20, 1-2x	Y
1.5.1.20	Methylenetetrahydrofolate reductase	ABQ60279.1	20	19	Y
1.5.1.-[a]	Flavin reductase domain containing protein	ABQ60228.1	20	7, 5-2x, 4-3x	Y
1.5.3.1	Sarcosine oxidase beta subunit	ABQ60177.1	0	0	N
		ABQ61310.1	20	14, 1-2x	Y
	Sarcosine oxidase alpha subunit	ABQ61036.1	20	15	Y
1.5.3.1	Predicted monomeric Sarcosine oxidase	ABQ62932.1	20	13	Y
1.5.5.1	Electron transferring flavoprotein-ubiquinone oxidoreductase (ETF-QO)	ABQ61337.1	20	21	Y
1.6.5.2	WrpA-type FMN-dependent NADH:quinone oxidoreductase	ABQ60884.1	20	13	Y
1.6.99.1	NADPH dehydrogenase (Old yellow enzyme)	ABQ62422.1	16	4	N
1.6.-.-	NADH dehydrogenase	ABQ62704.1	17	13	N
1.7.-.-[a]	Predicted NAD(P)H nitroreductase	ABQ60834.1	20	19	Y
1.8.1.2	Assimilatory sulphite reductase (NADPH) alpha component cluster	WP_006015252.1	This subunit does not bind flavin		
		WP_006015255.1	2	0	N
		WP_006015257.1	13	10	N
		WP_006015259.1	This subunit does not bind flavin		
1.8.1.4	Dihydrolipoyl dehydrogenase (lpdA-1)	ABQ62466.1	20	6	Y
1.8.1.4	Dihydrolipoyl dehydrogenase (IpdA-2)	ABQ60398.1	20	24, 2-2x	Y
1.8.1.4	Dihydrolipoyl dehydrogenase (IpdA-3)	ABQ61458.1	20	17, 8-2x	Y
1.8.1.7	Glutathione-disulphide reductase	ABQ61016.1	20	20	Y
1.8.1.9	Thioredoxin-disulphide reductase	ABQ60123.1	20	24, 2-2x	Y
1.8.1.9	Predicted thioredoxin-disulphide reductase	ABQ61134.1	19	11	N
1.8.5.B1	Peptide-methionine (S)-S-oxide reductase (quinone).	ABQ62365.1	20	10, 1-2x	Y

	MsrP catalytic subunit. MsrQ heme-binding subunit	ABQ62343.1	This subunit does not bind flavin		
1.13.11.32	Nitronate monooxygenase (formerly 2-nitropropane dioxygenase NPD)	ABQ62537.1	15	21	N
1.13.11.79	Predicted aerobic 5,6-dimethylbenzimidazole synthase (BluB)	ABQ62404.1	18	15	N
1.14.13.1	Predicted Salicylate hydroxylase	ABQ60137.1	19	11, 8-2x	N
1.14.13.1	Predicted Salicylate hydroxylase	ABQ60978.1	17	4	N
1.14.13.-[a]	Predicted UbiH/COQ6 monooxygenase family	ABQ60166.1	20	20, 4-2x, 1-3x, 1-4x	Y
1.14.13. -[a]	UbiH/UbiF family hydroxylase	ABQ62553.1	19	15, 2-2x	N
1.14.13.2	4-hydroxybenzoate 3-monooxygenase	ABQ62030.1	18	15	N
1.14.14.3	Bacterial luciferase	ABQ60348.1	19	18	N
1.16.1.4	Cob(II)alamin reductase	ABQ60249.1	20	24	Y
1.17.1.4	Xanthine dehydrogenase, small subunit	ABQ61298.1	19	17	N
1.18.1.3-5	Predicted Ferredoxin/rubredoxin/ putidaredoxin NAD^+ Reductase	ABQ62051.1	17	16	N
1.18.1.2	Ferredoxin-$NADP^+$ reductase	ABQ61707.1	20	20	Y
1.-.-.-[a]	Predicted nitroreductase family protein	ABQ62091.1	20	7	Y
2.1.1.74	Methylenetetrahydrofolate-tRNA-(uracil54-C5-)-methyltransferase NAD(P)H oxidase	ABQ61275.1	18	18	N
2.1.1.229	tRNA (carboxymethyluridine34-5-O)-methyltransferase	ABQ60378.1	20	25	Y
2.2.1.6	Acetolactate synthase 3 catalytic subunit	ABQ60081.1	20	21	Y
2.5.1.9	Riboflavin synthase alpha subunit	ABQ60518.1	20	20	Y
2.7.7.2<br>2.7.1.26	Bifunctional riboflavin kinase/FAD synthase	ABQ62831.1	19	22	N
2.7.1.180	FAD:protein FMN transferase	ABQ62066.1	16	4	N
2.7.13.3	Blue-light-activated histidine kinase	ABQ62113.1	19	2	N
4.1.1.36<br>6.3.2.5	Coenzyme A biosynthesis bifunctional protein: Phosphopantothenoylcysteine decarboxylase and Phosphopantothenate-cysteine ligase (CTP)	ABQ62036.1	17	23	N
4.2.3.5	Chorismate synthase	ABQ60200.1	19	21	N
7.1.1.2	NADH-quinone oxidoreductase subunit F (H^+ translocating)	ABQ60521.1	20	23	Y
--	Electron transferring flavoprotein (ETF) alpha subunit	ABQ61011.1	20	21	Y
	Electron transferring flavoprotein (ETF) beta subunit	ABQ60428.1	20	21	Y
--	Protein NrdI	ABQ62891.1	20	7	Y

[a] Identified as flavoenzyme, but available information does not allow to fully predict its activity.

[b] "a-bx", indicates that in a number "a" of species there is a number "b" of isoforms of the protein. In red are highlighted those proteins found in all evaluated alpha-proteobacteria.
[c] Y, yes, conserved in the 20 *Brucella* species evaluated. N, no, not conserved in the 20 Brucella species evaluated.

Table SP7. Experimental data for flavoproteins found in *B. ovis* and reported in different pathogens as contributors to virulence, survival, biofilm formation and/or infection.

Protein	Brucella core	Protein Code	Representative Species	REFERENCE
Glycerol-3-phosphate dehydrogenase	Y	ABQ60174.1	*Pseudomonas aeruginosa, Mycoplasma spp*	[4, 5]
D-erythritol 1-phosphate dehydrogenase	N	ABQ62056.1	*Brucella spp.*	[6]
Choline dehydrogenase (Glucose-methanol-choline GMC family)	N	ABQ61350.1	*E. coli, Acinetobacter baylyi, P. aeruginosa*	[7-9]
Choline dehydrogenase (GMC family)	N	ABQ60630.1		
Choline dehydrogenase (GMC family, membrane bound)	N	ABQ62100.1		
PyrD dihydroorotate dehydrogenase 2 (quinone)	Y	ABQ61413.1	*Pseudomonas aeruginosa*	[10]
Succinate dehydrogenase flavoprotein subunit	Y	ABQ61077.1	*E. coli, Salmonella enterica, Legionella pneumphila, Candida albicans, Xanthomonas oryzae pv. Oryzae*	[11-16]
Isovaleryl-CoA dehydrogenase	Y	ABQ60382.1	*Magnaporthe oryzae*	[17]
Acyl-CoA dehydrogenase	N	ABQ62576.1	*Burkholderia cenocepacia, Mycobacterium spp*	[18-20]
Acyl-CoA dehydrogenase	N	ABQ62784.1		
Acyl-CoA dehydrogenase	N	ABQ62889.1		
Glutamate synthase	Y	ABQ61996.1	*Brucella abortus, Xanthomonas oryzae pv. Oryzae*	[21, 22]
	N	ABQ62546.1		
Pyridoxamine 5'-phosphate oxidase	Y	ABQ60142.1	*Mycobacterium tuberculosis, Streptococcus pneumoniae*	[23, 24]
Predicted D-amino acid dehydrogenase small subunit	Y	ABQ61937.1	*Pseudomonas aeruginosa, Cryptococcus neoformans, Cryptococcus gattii*	[25, 26]
D-amino acid dehydrogenase	Y	ABQ62278.1		
Predicted D-amino acid dehydrogenase	N	ABQ62519.1		
Flavin reductase domain containing protein	Y	ABQ60228.1	*Streptococcus pneumoniae*	[27]
Sarcosine oxidase	Y	ABQ60177.1	*Pseudomonas aeruginosa PAO1*	[28]
		ABQ61310.1		
		ABQ61036.1		
WrpA type FMN-dependent NADH:quinone oxidoreductase	Y	ABQ60884.1	*Brucella abortus*	[29]
NADH dehydrogenase	N	ABQ62704.1	*Pseudomonas aeruginosa*	[30]
Assimilatory sulphite reductase (NADPH) alpha component cluster	N	WP_006015252.1	*Paracoccidioides brasiliensis, Salmonella enterica serovar Enteritidis*	[31, 32]
		WP_006015255.1		
		WP_006015257.1		
		WP_006015259.1		

Dihydrolipoyl dehydrogenase (IpdA-1)	Y	ABQ62466.1	*Mycoplasma gallisepticum, Vibrio parahaemolyticu*	[33, 34]
Dihydrolipoyl dehydrogenase (IpdA-2)	Y	ABQ60398.1		
Dihydrolipoyl dehydrogenase (IpdA-3)	Y	ABQ61458.1		
Peptide-methionine (S)-S-oxide reductase (quinone) (Msr). MsrP catalytic subunit. MsrQ heme-binding subunit	Y	ABQ62365.1	*Xanthomonas translucens pv. Undulosa, Staphylococcus Aureus, Helicobacter pylory*	[35-37]
		ABQ62343.1		
Predicted aerobic 5,6-dimethylbenzimidazole synthase (BluB)	N	ABQ62404.1	*Listeria monocytogenes, Mycobacterium spp, Brucella spp, Vibrio spp, Sinorhizobium meliloti*	[38-40]
Predicted Salicylate hydroxylase	N	ABQ60978.1	*Verticillium dahliae, Peronospora parasítica, Ustilago maydis, Candidatus Liberibacter asiaticus*	[41-44]
Cob(II)alamin reductase	Y	ABQ60249.1	*Listeria monocytogenes, Brucella ovis, Sinorhizobium meliloti*	[38, 45, 46]
Methylenetetrahydrofolate-tRNA-(uracil54-C5-)-methyltransferase NAD(P)H oxidase	N	ABQ61275.1	*Mycoplasma bovis, Pseudomonas aeruginosa, Salmonella enterica Serovar typhimurium, Streptococcus pyogenes*	[47-50]
tRNA (carboxymethyluridine34-5-O)-methyltransferase	Y	ABQ60378.1	*Mycoplasma bovis, Pseudomonas aeruginosa, Salmonella enterica Serovar typhimurium, Streptococcus pyogenes,*	[47-50]
Blue-light-activated histidine kinase	Only lacks in one	ABQ62113.1	*Brucella spp., Brucella abortus*	[51-53]
Chorismate synthase	N	ABQ60200.1	*Shigella flexneri, Burkholderia pseudomallei, Xanthomonas oryzae pathovar oryzae*	[54-56]
Protein NrdI	Y	ABQ62891.1	*Streptococcus sanguinis, Mycobacterium tuberculosis*	[57, 58]

SUPLEMENTARY FIGURES

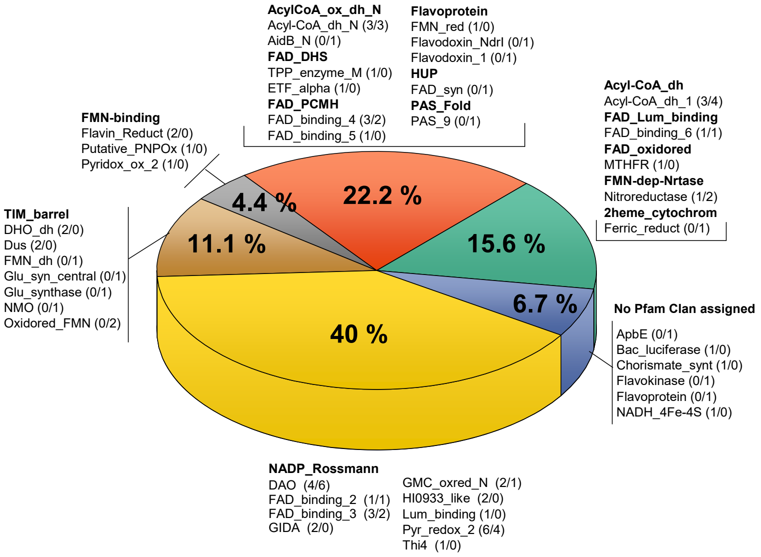

Figure SP1: Pie chart of the clans and domains contributing to bind flavins in the *B. ovis* flavoproteome. Clan names are highlighted in bold. Overall percentages are calculated based on the number of domains directly contributing to bind flavins in each clan. NADP_Rossman, TIM_barrel and FMN-binding clans are shown individually, while the rest are grouped attending to the number of families found in each: two (orange) or one (green). The blue portion includes domain families with no clan assigned. The number for a particular domain presented in each chromosome is denoted in brackets, as *N* in CI/*N* in CII.

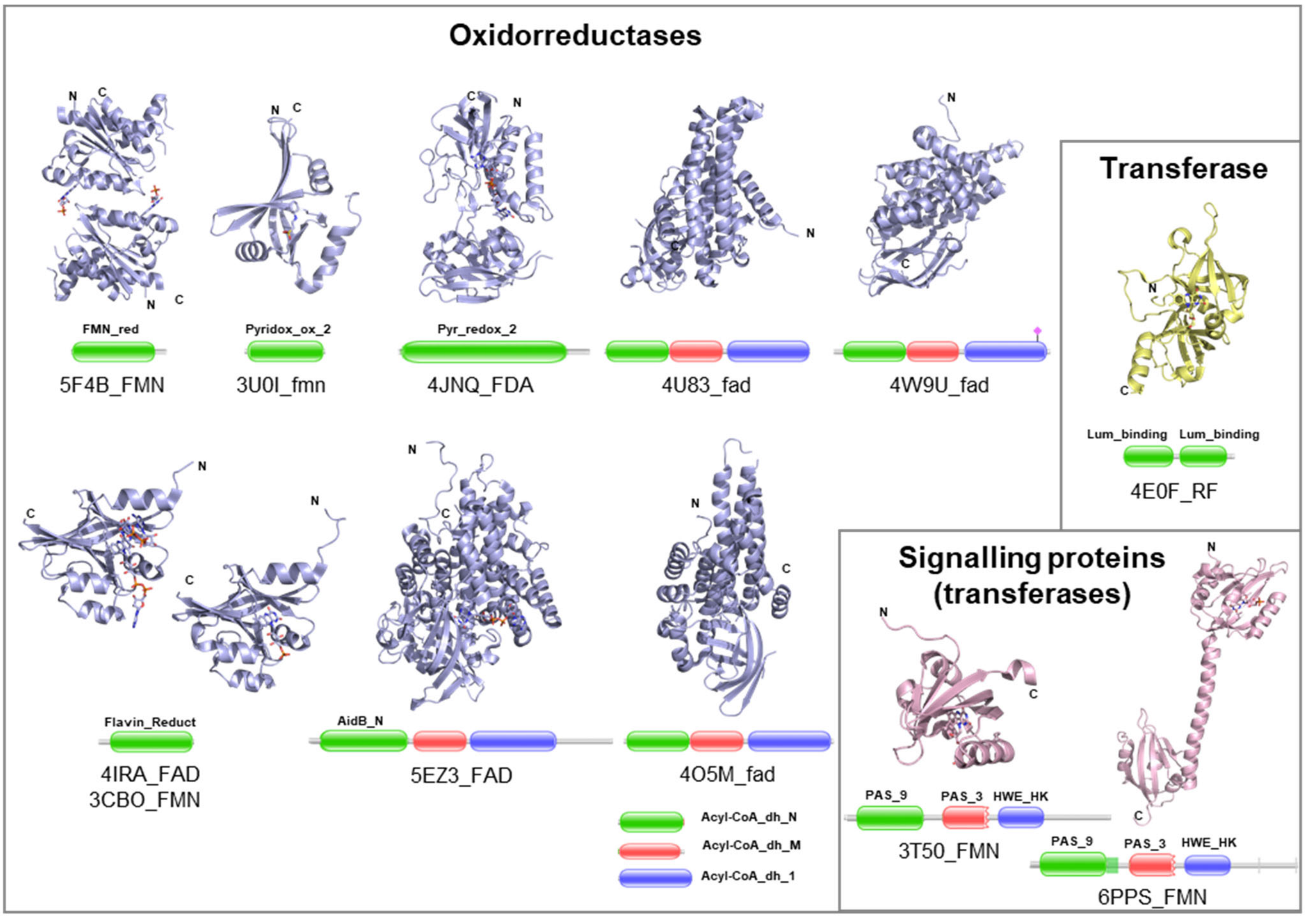

Figure SP2: Cartoon representation of 3D structures of proteins of *Brucella* that putatively bind flavin cofactors. Cofactors, when present, are in sticks and written after the PDB code (see Table SP2 for nomenclature). Below each structure its custom Pfam-style domain graphic is shown. Four oxidoreductases share the same legend for the assembly Acyl-CoA_dh_N, Acyl-CoA_dh_M and Acyl-CoA_dh_1 and it is placed under the main chart.

A

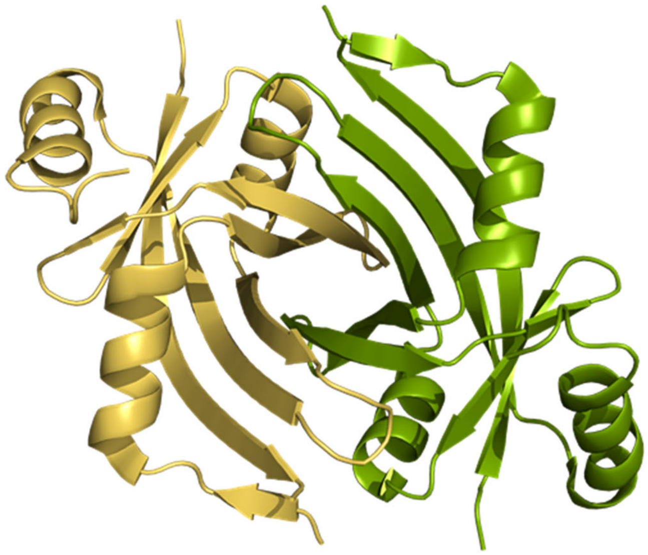

B

Brucella ovis	5' ← 3'	5' → 3'	5' ← 3'	5' → 3'	5' ← 3'
	crcB	BOV_RS06570	BOV_RS06575	BOV_RS17790	BOV_RS06590
	fluoride efflux transporter CrcB	hypothetical protein	Pyridoxamine 5'-phosphate oxidase family protein	IS5 family transposase	PhzF family phenazine biosynthesis protein
Brucella abortus	5' ← 3'	5' → 3'	5' ← 3'	5' ← 3'	5' ← 3'
	crcB	BAB_RS22570	BAB_RS22575	BAB_RS22580	BAB_RS22585
	fluoride efflux transporter CrcB	hypothetical protein	Pyridoxamine 5'-phosphate oxidase family protein	PhzF family phenazine biosynthesis protein	hypothetical protein
Brucella suis	5' ← 3'	5' → 3'	5' ← 3'	5' ← 3'	5' ← 3'
	crcB	BR_RS06340	BR_RS06345	BR_RS06350	BR_RS06355
	fluoride efflux transporter CrcB	hypothetical protein	Pyridoxamine 5'-phosphate oxidase family protein	PhzF family phenazine biosynthesis protein	hypothetical protein
Brucella melitensis	5' → 3'	5' → 3'	5' → 3'	5' → 3'	5' → 3'
	BME_RS03135	BME_RS03140	BME_RS03145	BME_RS03150	crcB
	NUDIX hydrolase (pseudogen)	PhzF family phenazine biosynthesis protein	Pyridoxamine 5'-phosphate oxidase family protein	hypothetical protein	fluoride efflux transporter CrcB
Brucella canis	5' ← 3'	5' → 3'	5' ← 3'	5' ← 3'	5' ← 3'
	crcB	BCAN_RS06360	BCAN_RS06365	BCAN_RS06370	BCAN_RS06375
	fluoride efflux transporter CrcB	hypothetical protein	Pyridoxamine 5'-phosphate oxidase family protein	PhzF family phenazine biosynthesis protein	NUDIX hydrolase
Brucella microti	5' ← 3'	5' → 3'	5' ← 3'	5' ← 3'	5' ← 3'
	crcB	BMI_RS06395	BMI_RS06400	BMI_RS06405	BMI_RS06410
	fluoride efflux transporter CrcB	hypothetical protein	Pyridoxamine 5'-phosphate oxidase family protein	PhzF family phenazine biosynthesis protein	NUDIX hydrolase
Ochrobactrum anthropi	5' → 3'	5' → 3'	5' → 3'	5' ← 3'	5' → 3'
	DR92_RS16560	DR92_RS16565	DR92_RS16570	DR92_RS16575	DR92_RS16580
	NUDIX hydrolase	PhzF family phenazine biosynthesis protein	Pyridoxamine 5'-phosphate oxidase family protein	universal stress protein	glycoside hydrolase family 10 protein

Figure SP3: Structure and conservation of the pyridoxamine 5'-phosphate oxidase family protein in *Brucella*. (A) Crystal structure of the protein from *B. melitensis* (PDB 3U0I, unpublished), predicted as a dimer (monomers in yellow and green). (B) Gene context for the genes encoding this protein in different *Brucella*.

REFERENCES

[1] K.P. Williams, B.W. Sobral, A.W. Dickerman, A robust species tree for the alphaproteobacteria, J Bacteriol 189(13) (2007) 4578-86.
[2] J. Velasco, C. Romero, I. López-Goñi, J. Leiva, R. Díaz, I. Moriyón, Evaluation of the relatedness of Brucella spp. and Ochrobactrum anthropi and description of Ochrobactrum intermedium sp. nov., a new species with a closer relationship to Brucella spp, Int J Syst Bacteriol 48 Pt 3 (1998) 759-68.
[3] C. Farr Zuend, J.F. Nomellini, J. Smit, M.S. Horwitz, A Caulobacter crescentus Microbicide Protects from Vaginal Infection with HIV-1, J Virol 93(18) (2019).
[4] J.B. Daniels, J. Scoffield, J.L. Woolnough, L. Silo-Suh, Impact of glycerol-3-phosphate dehydrogenase on virulence factor production by Pseudomonas aeruginosa, Can J Microbiol 60(12) (2014) 857-63.
[5] C. Blötz, J. Stülke, Glycerol metabolism and its implication in virulence in Mycoplasma, FEMS Microbiol Rev 41(5) (2017) 640-652.
[6] T. Barbier, F. Collard, A. Zúñiga-Ripa, I. Moriyón, T. Godard, J. Becker, C. Wittmann, E. Van Schaftingen, J.J. Letesson, Erythritol feeds the pentose phosphate pathway via three new isomerases leading to D-erythrose-4-phosphate in Brucella, Proc Natl Acad Sci U S A 111(50) (2014) 17815-20.
[7] A. Scholz, J. Stahl, V. de Berardinis, V. Müller, B. Averhoff, Osmotic stress response in Acinetobacter baylyi: identification of a glycine-betaine biosynthesis pathway and regulation of osmoadaptive choline uptake and glycine-betaine synthesis through a choline-responsive BetI repressor, Environ Microbiol Rep 8(2) (2016) 316-22.
[8] Y. Wang, C. Tang, X. Yu, M. Xia, H. Yue, Distribution of serotypes and virulence-associated genes in pathogenic Escherichia coli isolated from ducks, Avian Pathol 39(4) (2010) 297-302.
[9] M.J. Wargo, Choline catabolism to glycine betaine contributes to Pseudomonas aeruginosa survival during murine lung infection, PLoS One 8(2) (2013) e56850.
[10] Q. Guo, Y. Wei, B. Xia, Y. Jin, C. Liu, X. Pan, J. Shi, F. Zhu, J. Li, L. Qian, X. Liu, Z. Cheng, S. Jin, J. Lin, W. Wu, Identification of a small molecule that simultaneously suppresses virulence and antibiotic resistance of Pseudomonas aeruginosa, Sci Rep 6 (2016) 19141.
[11] C.J. Kuo, S.T. Wang, C.M. Lin, H.C. Chiu, C.R. Huang, D.Y. Lee, G.D. Chang, T.C. Chou, J.W. Chen, C.S. Chen, A multi-omic analysis reveals the role of fumarate in regulating the virulence of enterohemorrhagic Escherichia coli, Cell Death Dis 9(3) (2018) 381.
[12] A. Karsi, N. Gülsoy, E. Corb, P.R. Dumpala, M.L. Lawrence, High-throughput bioluminescence-based mutant screening strategy for identification of bacterial virulence genes, Appl Environ Microbiol 75(7) (2009) 2166-75.
[13] Y.J. Zhang, J. Li, W. Zhao, M.G. Zhou, A single amino acid substitution in the SdhB protein of succinate dehydrogenase determines resistance to amicarthiazol in Xanthomonas oryzae pv. oryzae, Pest Manag Sci 66(6) (2010) 627-33.
[14] E.A. Creasey, R.R. Isberg, The protein SdhA maintains the integrity of the Legionella-containing vacuole, Proc Natl Acad Sci U S A 109(9) (2012) 3481-6.
[15] H. Brüggemann, A. Hagman, M. Jules, O. Sismeiro, M.A. Dillies, C. Gouyette, F. Kunst, M. Steinert, K. Heuner, J.Y. Coppée, C. Buchrieser, Virulence strategies for infecting phagocytes deduced from the in vivo transcriptional program of Legionella pneumophila, Cell Microbiol 8(8) (2006) 1228-40.
[16] S. Bi, Q.Z. Lv, T.T. Wang, B.B. Fuchs, D.D. Hu, C.G. Anastassopoulou, A. Desalermos, M. Muhammed, C.L. Wu, Y.Y. Jiang, E. Mylonakis, Y. Wang, SDH2 is involved in proper hypha formation and virulence in Candida albicans, Future Microbiol 13 (2018) 1141-1156.
[17] Y. Li, X. Zheng, M. Zhu, M. Chen, S. Zhang, F. He, X. Chen, J. Lv, M. Pei, Y. Zhang, W. Wang, J. Zhang, M. Wang, Z. Wang, G. Li, G. Lu, Mediated Leucine Catabolism Is Required for Vegetative Growth, Conidiation and Full Virulence of the Rice Blast Fungus, Front Microbiol 10 (2019) 444.
[18] S. Subramoni, D.T. Nguyen, P.A. Sokol, Burkholderia cenocepacia ShvR-regulated genes that influence colony morphology, biofilm formation, and virulence, Infect Immun 79(8) (2011) 2984-97.
[19] V. Hughes, S. Smith, A. Garcia-Sanchez, J. Sales, K. Stevenson, Proteomic comparison of Mycobacterium avium subspecies paratuberculosis grown in vitro and isolated from clinical cases of ovine paratuberculosis, Microbiology (Reading) 153(Pt 1) (2007) 196-205.
[20] M. Yang, R. Lu, K.E. Guja, M.F. Wipperman, J.R. St Clair, A.C. Bonds, M. Garcia-Diaz, N.S. Sampson, Unraveling Cholesterol Catabolism in *Mycobacterium tuberculosis*: ChsE4-ChsE5 $\alpha_2\beta_2$ Acyl-CoA Dehydrogenase Initiates β-Oxidation of 3-Oxo-cholest-4-en-26-oyl CoA, ACS Infect Dis 1(2) (2015) 110-125.

[21] P.C. Hong, R.M. Tsolis, T.A. Ficht, Identification of genes required for chronic persistence of Brucella abortus in mice, Infect Immun 68(7) (2000) 4102-7.
[22] A. Pandey, S.K. Ray, R.V. Sonti, R. Rajeshwari, gltB/D mutants of Xanthomonas oryzae pv. oryzae are virulence deficient, Curr Microbiol 68(1) (2014) 105-12.
[23] K. Ankisettypalli, J.J. Cheng, E.N. Baker, G. Bashiri, PdxH proteins of mycobacteria are typical members of the classical pyridoxine/pyridoxamine 5'-phosphate oxidase family, FEBS Lett 590(4) (2016) 453-60.
[24] S. El Qaidi, J. Yang, J.R. Zhang, D.W. Metzger, G. Bai, The vitamin B_6 biosynthesis pathway in Streptococcus pneumoniae is controlled by pyridoxal 5'-phosphate and the transcription factor PdxR and has an impact on ear infection, J Bacteriol 195(10) (2013) 2187-96.
[25] K.E. Oliver, L. Silo-Suh, Impact of D-amino acid dehydrogenase on virulence factor production by a Pseudomonas aeruginosa, Can J Microbiol 59(9) (2013) 598-603.
[26] Y.C. Chang, A. Khanal Lamichhane, J. Bradley, L. Rodgers, P. Ngamskulrungroj, K.J. Kwon-Chung, Differences between Cryptococcus neoformans and Cryptococcus gattii in the Molecular Mechanisms Governing Utilization of D-Amino Acids as the Sole Nitrogen Source, PLoS One 10(7) (2015) e0131865.
[27] G.I. Morozov, N. Porat, T. Kushnir, H. Najmuldeen, A. Adawi, V. Chalifa-Caspi, R. Benisty, A. Ohayon, O. Liron, S. Azriel, I. Malka, S. Dotan, M. Portnoi, A.A. Piotrowski, D. Kafka, B. Hajaj, T. Fishilevich, M. Shagan, M. Tal, R. Ellis, D.A. Morrison, A.M. Mitchell, T.J. Mitchell, R. Dagan, H. Yesilkaya, Y.M. Nebenzahl, Flavin Reductase Contributes to Pneumococcal Virulence by Protecting from Oxidative Stress and Mediating Adhesion and Elicits Protection Against Pneumococcal Challenge, Sci Rep 8(1) (2018) 314.
[28] L.A. Gallagher, C. Manoil, Pseudomonas aeruginosa PAO1 kills Caenorhabditis elegans by cyanide poisoning, J Bacteriol 183(21) (2001) 6207-14.
[29] J. Herrou, D.M. Czyż, J.W. Willett, H.S. Kim, G. Chhor, G. Babnigg, Y. Kim, S. Crosson, WrpA Is an Atypical Flavodoxin Family Protein under Regulatory Control of the Brucella abortus General Stress Response System, J Bacteriol 198(8) (2016) 1281-93.
[30] A. Torres, N. Kasturiarachi, M. DuPont, V.S. Cooper, J. Bomberger, A. Zemke, NADH Dehydrogenases in *Pseudomonas aeruginosa* Growth and Virulence, Front Microbiol 10 (2019) 75.
[31] J.F. Menino, M. Saraiva, J. Gomes-Rezende, M. Sturme, J. Pedrosa, A.G. Castro, P. Ludovico, G.H. Goldman, F. Rodrigues, P. brasiliensis virulence is affected by SconC, the negative regulator of inorganic sulfur assimilation, PLoS One 8(9) (2013) e74725.
[32] B. Liu, W. Hou, K. Li, Q. Chen, Y. Liu, T. Yue, Specific gene SEN1393 contributes to higher survivability of Salmonella Enteritidis in egg white by regulating sulfate assimilation pathway, Int J Food Microbiol 337 (2021) 108927.
[33] P. Hudson, T.S. Gorton, L. Papazisi, K. Cecchini, S. Frasca, S.J. Geary, Identification of a virulence-associated determinant, dihydrolipoamide dehydrogenase (lpd), in Mycoplasma gallisepticum through in vivo screening of transposon mutants, Infect Immun 74(2) (2006) 931-9.
[34] Y. He, H. Wang, L. Chen, Comparative secretomics reveals novel virulence-associated factors of Vibrio parahaemolyticus, Front Microbiol 6 (2015) 707.
[35] A. Sharma, D. Sharma, S.K. Verma, Proteome wide identification of iron binding proteins of Xanthomonas translucens pv. undulosa: focus on secretory virulent proteins, Biometals 30(1) (2017) 127-141.
[36] S.J. Sasindran, S. Saikolappan, S. Dhandayuthapani, Methionine sulfoxide reductases and virulence of bacterial pathogens, Future Microbiol 2(6) (2007) 619-30.
[37] A. Gennaris, B. Ezraty, C. Henry, R. Agrebi, A. Vergnes, E. Oheix, J. Bos, P. Leverrier, L. Espinosa, J. Szewczyk, D. Vertommen, O. Iranzo, J.F. Collet, F. Barras, Repairing oxidized proteins in the bacterial envelope using respiratory chain electrons, Nature 528(7582) (2015) 409-412.
[38] J.M. Anast, T.A. Bobik, S. Schmitz-Esser, The Cobalamin-Dependent Gene Cluster of *Listeria monocytogenes*: Implications for Virulence, Stress Response, and Food Safety, Front Microbiol 11 (2020) 601816.
[39] M.E. Taga, N.A. Larsen, A.R. Howard-Jones, C.T. Walsh, G.C. Walker, BluB cannibalizes flavin to form the lower ligand of vitamin B12, Nature 446(7134) (2007) 449-53.
[40] G.R. Campbell, M.E. Taga, K. Mistry, J. Lloret, P.J. Anderson, J.R. Roth, G.C. Walker, Sinorhizobium meliloti bluB is necessary for production of 5,6-dimethylbenzimidazole, the lower ligand of B12, Proc Natl Acad Sci U S A 103(12) (2006) 4634-9.
[41] X. Luo, T. Tian, X. Tan, Y. Zheng, C. Xie, Y. Xu, X. Yang, VdNPS, a Nonribosomal Peptide Synthetase, Is Involved in Regulating Virulence in *Verticillium dahliae*, Phytopathology 110(8) (2020) 1398-1409.

[42] N.M. Donofrio, T.P. Delaney, Abnormal callose response phenotype and hypersusceptibility to Peronospoara parasitica in defence-compromised arabidopsis nim1-1 and salicylate hydroxylase-expressing plants, Mol Plant Microbe Interact 14(4) (2001) 439-50.
[43] F. Rabe, Z. Ajami-Rashidi, G. Doehlemann, R. Kahmann, A. Djamei, Degradation of the plant defence hormone salicylic acid by the biotrophic fungus Ustilago maydis, Mol Microbiol 89(1) (2013) 179-88.
[44] J. Li, Z. Pang, P. Trivedi, X. Zhou, X. Ying, H. Jia, N. Wang, 'Candidatus Liberibacter asiaticus' Encodes a Functional Salicylic Acid (SA) Hydroxylase That Degrades SA to Suppress Plant Defenses, Mol Plant Microbe Interact 30(8) (2017) 620-630.
[45] S. Shim, Y.B. Im, M. Jung, W.B. Park, H.S. Yoo, Genes Related to Intracellular Survival of *Brucella abortus* in THP-1 Macrophage Cells, J Microbiol Biotechnol 28(10) (2018) 1736-1748.
[46] G. Lu, Y. Lindqvist, G. Schneider, U. Dwivedi, W. Campbell, Structural studies on corn nitrate reductase: refined structure of the cytochrome b reductase fragment at 2.5 Å, its ADP complex and an active-site mutant and modeling of the cytochrome b domain, J Mol Biol 248(5) (1995) 931-48.
[47] Y. Guo, H. Zhu, J. Wang, J. Huang, F.A. Khan, J. Zhang, A. Guo, X. Chen, TrmFO, a Fibronectin-Binding Adhesin of Mycoplasma bovis, Int J Mol Sci 18(8) (2017).
[48] R. Gupta, T.R. Gobble, M. Schuster, GidA posttranscriptionally regulates rhl quorum sensing in Pseudomonas aeruginosa, J Bacteriol 191(18) (2009) 5785-92.
[49] D.C. Shippy, J.A. Heintz, R.M. Albrecht, N.M. Eakley, A.K. Chopra, A.A. Fadl, Deletion of glucose-inhibited division (gidA) gene alters the morphological and replication characteristics of Salmonella enterica Serovar typhimurium, Arch Microbiol 194(6) (2012) 405-12.
[50] K.H. Cho, M.G. Caparon, tRNA modification by GidA/MnmE is necessary for Streptococcus pyogenes virulence: a new strategy to make live attenuated strains, Infect Immun 76(7) (2008) 3176-86.
[51] T.E. Swartz, T.S. Tseng, M.A. Frederickson, G. Paris, D.J. Comerci, G. Rajashekara, J.G. Kim, M.B. Mudgett, G.A. Splitter, R.A. Ugalde, F.A. Goldbaum, W.R. Briggs, R.A. Bogomolni, Blue-light-activated histidine kinases: two-component sensors in bacteria, Science 317(5841) (2007) 1090-3.
[52] J. Rinaldi, I. Fernández, H. Shin, G. Sycz, S. Gunawardana, I. Kumarapperuma, J.M. Paz, L.H. Otero, M.L. Cerutti, Á. Zorreguieta, Z. Ren, S. Klinke, X. Yang, F.A. Goldbaum, Dimer Asymmetry and Light Activation Mechanism in *Brucella* Blue-Light Sensor Histidine Kinase, mBio 12(2) (2021).
[53] J. Rinaldi, M. Arrar, G. Sycz, M.L. Cerutti, P.M. Berguer, G. Paris, D.A. Estrín, M.A. Martí, S. Klinke, F.A. Goldbaum, Structural Insights into the HWE Histidine Kinase Family: The Brucella Blue Light-Activated Histidine Kinase Domain, J Mol Biol 428(6) (2016) 1165-1179.
[54] A. Cersini, A.M. Salvia, M.L. Bernardini, Intracellular multiplication and virulence of Shigella flexneri auxotrophic mutants, Infect Immun 66(2) (1998) 549-57.
[55] T. Srilunchang, T. Proungvitaya, S. Wongratanacheewin, R. Strugnell, P. Homchampa, Construction and characterization of an unmarked aroC deletion mutant of Burkholderia pseudomallei strain A2, Southeast Asian J Trop Med Public Health 40(1) (2009) 123-30.
[56] E.S. Song, Y.J. Park, T.H. Noh, Y.T. Kim, J.G. Kim, H. Cho, B.M. Lee, Functional analysis of the aroC gene encoding chorismate synthase from Xanthomonas oryzae pathovar oryzae, Microbiol Res 167(6) (2012) 326-31.
[57] H. Zheng, L. Lu, B. Wang, S. Pu, X. Zhang, G. Zhu, W. Shi, L. Zhang, H. Wang, S. Wang, G. Zhao, Y. Zhang, Genetic basis of virulence attenuation revealed by comparative genomic analysis of Mycobacterium tuberculosis strain H37Ra versus H37Rv, PLoS One 3(6) (2008) e2375.
[58] D.V. Rhodes, K.E. Crump, O. Makhlynets, M. Snyder, X. Ge, P. Xu, J. Stubbe, T. Kitten, Genetic characterization and role in virulence of the ribonucleotide reductases of Streptococcus sanguinis, J Biol Chem 289(9) (2014) 6273-87.
